# Identification of inhibitors from a functional food-based plant Perillae Folium against hyperuricemia via metabolomics profiling, network pharmacology and all-atom molecular dynamics simulations

**DOI:** 10.3389/fendo.2024.1320092

**Published:** 2024-02-16

**Authors:** Chuanghai Wu, Ann Rann Wong, Qinghong Chen, Shuxuan Yang, Meilin Chen, Xiaomin Sun, Lin Zhou, Yanyan Liu, Angela Wei Hong Yang, Jianlu Bi, Andrew Hung, Hong Li, Xiaoshan Zhao

**Affiliations:** ^1^ School of Traditional Chinese Medicine, Southern Medical University, Guangzhou, China; ^2^ School of Health and Biomedical Sciences, STEM College, RMIT University, Bundoora, VIC, Australia; ^3^ Endocrinology Department, Nanfang Hospital, Southern Medical University, Guangzhou, China; ^4^ Endocrinology Department, Guangdong Second Traditional Chinese Medicine Hospital, Guangzhou, China; ^5^ School of Science, STEM College, RMIT University, Melbourne, VIC, Australia

**Keywords:** functional food, hyperuricemia, metabolomics, molecular docking, molecular dynamics simulation, *in silico* analysis, chemical structure analysis

## Abstract

**Introduction:**

Hyperuricemia (HUA) is a metabolic disorder caused by purine metabolism dysfunction in which the increasing purine levels can be partially attributed to seafood consumption. Perillae Folium (PF), a widely used plant in functional food, has been historically used to mitigate seafood-induced diseases. However, its efficacy against HUA and the underlying mechanism remain unclear.

**Methods:**

A network pharmacology analysis was performed to identify candidate targets and potential mechanisms involved in PF treating HUA. The candidate targets were determined based on TCMSP, SwissTargetPrediction, Open Targets Platform, GeneCards, Comparative Toxicogenomics Database, and DrugBank. The potential mechanisms were predicted via Gene Ontology (GO) and Kyoto Gene and Genome Encyclopedia (KEGG) analyses. Molecular docking in AutoDock Vina and PyRx were performed to predict the binding affinity and pose between herbal compounds and HUA-related targets. A chemical structure analysis of PF compounds was performed using OSIRIS DataWarrior and ClassyFire. We then conducted virtual pharmacokinetic and toxicity screening to filter potential inhibitors. We further performed verifications of these inhibitors’ roles in HUA through molecular dynamics (MD) simulations, text-mining, and untargeted metabolomics analysis.

**Results:**

We obtained 8200 predicted binding results between 328 herbal compounds and 25 potential targets, and xanthine dehydrogenase (XDH) exhibited the highest average binding affinity. We screened out five promising ligands (scutellarein, benzyl alpha-D-mannopyranoside, elemol, diisobutyl phthalate, and (3R)-hydroxy-beta-ionone) and performed MD simulations up to 50 ns for XDH complexed to them. The scutellarein-XDH complex exhibited the most satisfactory stability. Furthermore, the text-mining study provided laboratory evidence of scutellarein’s function. The metabolomics approach identified 543 compounds and confirmed the presence of scutellarein. Extending MD simulations to 200 ns further indicated the sustained impact of scutellarein on XDH structure.

**Conclusion:**

Our study provides a computational and biomedical basis for PF treating HUA and fully elucidates scutellarein's great potential as an XDH inhibitor at the molecular level, holding promise for future drug design and development.

## 1 Introduction

Hyperuricemia (HUA) is defined as elevated urate in the circulation due to overproduction or underexcretion of uric acid (1, 2). In clinical practice, HUA is typically reported when serum urate exceeds 420 μmol/L (7 mg/dL) in males or 360 μmol/L (6 mg/dL) in females (3). In recent years, some epidemiological research indicated that an upward trend of prevalence of HUA in China was observed (4, 5). The incidence and prevalence rate of HUA in Chinese adults is 11.1 per 100 person-years and 15.1%, respectively (6, 7). In general, the widely observed consequences of HUA are gout and nephrolithiasis (8). Typically, HUA is deemed as the most decisive risk factor for the development of gout while the serum urate contributes to gout as well as nephrolithiasis in a concentration-dependent manner (9, 10). In addition to the above-mentioned consequences, asymptomatic HUA is also accompanied by a series of complications. Accumulative clinical studies suggested that elevated levels of uric acid are associated with increased risk of renal diseases, whether the participants are children, adolescents, or adults (11, 12). Furthermore, HUA is also recognized as a risk factor for kidney failure and all-cause mortality in patients with chronic kidney disease (13, 14). Mounting clinical evidence also showed that high serum uric acid levels were associated with hypertension (15, 16), stroke (17), type 2 diabetes (18), and metabolic syndrome (19).

To prevent the potential related comorbidities of HUA, urate-lowering agents are needed, which aim at reducing the production or increasing the excretion of uric acid. To inhibit the process of uric acid production, the first-line clinical drugs, including allopurinol and febuxostat, were designed mainly based on the xanthine oxidoreductase enzyme, a limiting enzyme in purine metabolism that is essential for converting hypoxanthine and xanthine into uric acid. This enzyme typically exists in two forms, xanthine dehydrogenase (XDH) and xanthine oxidase (XO) (20). In addition to lowering serum urate (21), the above XDH inhibitors were proven clinically to slow the progression of chronic kidney disease and reduce cardiovascular risks (22, 23). However, the clinical application of XDH inhibitors has been limited due to the reported adverse events. For instance, severe hypersensitivity reactions were reported when using allopurinol. Therefore, genotype testing of HLA-B*5801 is necessary for clinical applications and the medical burden is increasing. Additionally, febuxostat also has potential cardiovascular toxicity and hepatotoxicity (24). Therefore, it is urgent to identify new effective and non-toxic urate-lowering agents to meet the demand of an increasing proportion of the population with HUA.

In recent years, plant-based functional food has attracted considerable attention for its beneficial effects on health promotion, well-being, and diseases. Numerous laboratory evidence indicated that plant-based functional foods play a non-negligible role in the prevention and management of HUA (25). Perillae Folium (PF), namely the leaf of *Perilla frutescens* (L.) Britton or Perilla Leaf, also known as Zi su ye in Chinese Pin Yin, which can be seen as a representative plant-based functional food, has a long history of serving as a widely used, edible medicinal herb. PF is mainly cultivated in Hubei, Henan, and Sichuan provinces in China, and is harvested in summer. In traditional Chinese medicine theory, PF is characterized as having warm properties and spicy flavor, entering the Spleen and Lung meridians, and possesses the efficacy of relieving the exterior syndrome, dispelling cold, activating Qi, regulating the stomach, as well as detoxifying fish and crab poison (26). Pharmacological research reveals that PF may have bioactive properties including anti-allergy, anti-inflammatory, anti-oxidation, anti-cancer, anti-bacterial, and anti-depression effects (27). Several laboratory studies indicated that some bioactive constituents of PF showed impressive inhibitory effects on XDH (28–30). However, the compounds identified from PF are still limited and a more comprehensive and more in-depth study is greatly needed. Here, with the help of supercomputer-aided technology, we explored the mechanism of PF treatment for HUA, fully screened potential active ingredients, and verified them using an integrated multi-method approach, including untargeted metabolomics profiling, bioinformatics analysis, high-throughput virtual screening, cheminformatics analysis, and molecular dynamics (MD) simulations.

## 2 Materials and methods

**Figure 1:**
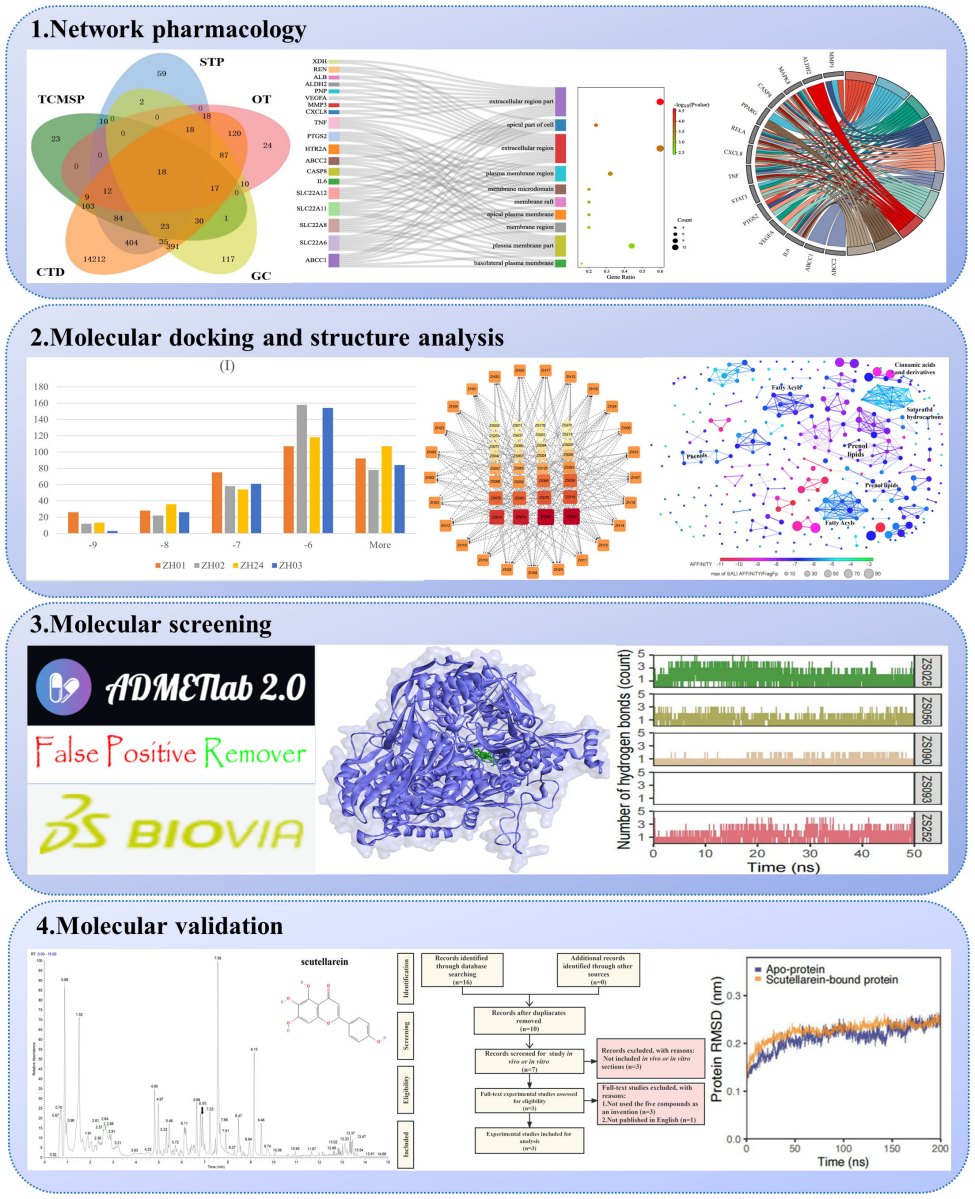
The workflow of the computational prediction.

The computational calculations in our work were conducted using the Sunway TaihuLight supercomputer (12-cores Chinese-designed SW26010 manycore 64-bit RISC processors) at the National Supercomputing Center in Wuxi (Wuxi, China), and the SiBioLead online MD simulation platform (https://sibiolead.com/) (GPU-based high-performance cluster system, running on Ubuntu OS, NVIDIA GeForce RTX3050 GPUs). **Figure 1** presents the holistic view of our research process.

### 2.1 Identification of Perillae Folium compounds

Compounds of PF were retrieved from the Traditional Chinese Medicine Systems Pharmacology Database and Analysis Platform (TCMSP), a systems pharmacology platform for Chinese herbal medicines (https://tcmsp-e.com/). The widely used database consists of 499 Chinese herbs, 29384 ingredients, 3311 targets, and 837 associated diseases. In addition, 12 key pharmacokinetic properties were included for each compound (31). Zi su ye, the pinyin name of PF, was used as a search term to gather information for each ingredient, including the molecular name, PubChem CID, MOL2 files, and the pharmacokinetic parameters.

Since all the identified compounds were regarded as potential ligands in our next stage of analysis, we collected the structure files in SDF format and the SMILES sequence of each ligand with the assistance of the PubChem database. MOL2 files downloaded from the TCMSP served as a substitute when the SDF files of a certain ligand were not available. The structures obtained above were then transformed into PDB format via Discovery Studio Visualizer 2019.

### 2.2 Analysis of metabolomics profiling

The Zi su ye dispensing granule (batch number: A2121691; Guangdong Yifang Pharmaceutical Co., Ltd.) was prescribed and supplied by Dr Xiaojuan Deng from the Liwan District People’s Hospital of Guangzhou. Untargeted metabolomics was carried out by the Shanghai Bioprofile Biotechnology Co., Ltd (Shanghai, China), which included sample preparation, extract analysis, and identification of metabolites. For the sample preparation, 50 mg of Zi su ye granule was mixed with 300 μL of pre-cooled methanol-aqueous solution (4:1, v/v) and then the sample was homogenized and broken. Subsequently, 700 μL of pre-cooled methanol-water solution (4:1, v/v) was added and the sample was treated with ultrasound in an ice bath for 20 minutes (ultrasound, Bioruptor, Diagenode). After incubating at -20°C for one hour, the sample was centrifuged at 16000 g at 4°C for 20 minutes and then the collected supernatant was vacuum-dried (Concentrator plus, Eppendorf). Finally, for further metabolic analysis, the supernatant was re-dissolved in 200 μL of methanol-aqueous solution (1:1, v/v) and was centrifuged again at 20000 g at 4°C for 15 minutes. In terms of extract analysis, a UHPLC (Shimadzu Nexera X2 LC-30AD, Shimadzu, Japan) coupled with Q-Exactive Plus (Thermo Scientific, San Jose, USA) was used. The conditions of UPLC were presented as follows: an ACQUITY UPLC® HSS T3 column (2.1×100 mm, 1.8 µm) (Waters, Milford, MA, USA) was selected. The mobile phase included solvent A (0.1% formic acid in water) and solvent B (100% acetonitrile). The gradient elution was set as follows: 0% B (0-2 minutes), linear gradient from 0% to 48% B (2-6 minutes), 48% to 100% B (6-10 minutes), maintained at 100% B (10-12 minutes), 100% to 0% B (12-12.1 minutes), kept at 0% B (12.1-15 minutes). The flow rate, temperature of column oven, and volume of injection were 0.3 mL/min, 40°C, and 6 μL, respectively. Mass spectrometry data acquisition was conducted in both positive and negative ion modes under parameters as follows, spray voltage: 3.8 kv (positive) and 3.2 kv (negative); capillary temperature: 320°C; sheath gas (nitrogen) flow: 30 arb (arbitrary units); Aux Gas flow: 5 arb; probe heater temperature: 350°C; S-Lens RF Level: 50; the range of precursor ion scan: 70-1050 m/z; full MS scan: 70000 at m/z 200 (resolution), 3×10^6^ [automatic gain control (AGC)], 100 ms [injection time (IT)]; MS/MS scan: 17500 at m/z 200 (resolution), 1×10^5^ (AGC), 50 ms (IT); isolation window for MS2: 2 m/z; normalized collision energy (Stepped): 20, 30, and 40. Furthermore, at the stage of metabolite identification, MS-DIAL was used for peak alignment, retention time correction, and peak area extraction for preprocessing the acquired raw MS data. Finally, metabolite identification was based on accuracy mass (mass tolerance < 10 ppm) and MS/MS data (mass tolerance < 0.02 Da), and the obtained metabolites were then matched with the metabolite standard library built by Bioprofile.

### 2.3 Acquisition of Perillae Folium’s potential targets for hyperuricemia

Typically, both ligand-related and disease-related targets are required for the identification of potential targets of a specific herb for a certain disease when conducting network pharmacology research. Concerning ligand-related targets, the TCMSP database was employed to obtain “related targets” by searching the herbal name, and all results were subsequently included. Additionally, the SwissTargetPrediction database (http://swisstargetprediction.ch/), a web tool commonly used for predicting the most probable protein targets of a specific small molecule, was applied by putting the SMILES sequences of compounds into the search box (32). Only those with a probability score greater than or equal to 0.1 are considered, consistent with the criterion set out in a previous paper (33). On the other hand, the identification of HUA-related targets was implemented using the following three disease databases: Open Targets Platform (https://platform.opentargets.org/) (34), GeneCards (https://www.genecards.org/) (35), and Comparative Toxicogenomics Database (CTD) (http://ctdbase.org/) (36). Next, each target name was converted into an official name corresponding to Homo sapiens via the UniProt database (http://www.uniprot.org) (37).

Subsequently, the two kinds of targets mentioned above were intersected and a Venn diagram was plotted to recapitulate the profile of these targets using jvenn (http://bioinfo.genotoul.fr/jvenn) (38). In addition, to comprehensively investigate the interactions between PF’s ingredients and HUA, some drug targets were also included in our analysis, whose identification processes are presented as follows. “Hyperuricemia”, as an indication, was entered into the search box in the DrugBank database (https://go.drugbank.com/) (39), and five clinical agents were found, including Allopurinol, Lesinurad, Probenecid, Rasburicase, and Sulfinpyrazone. Then their corresponding targets that act as inhibitors were identified, including XDH, SLC22A12, SLC22A11, SLC22A8, SLC22A6, ABCC1, and ABCC2. In addition, given that inhibiting purine nucleoside phosphorylase (PNP) to reduce the production of uric acid is one of the main therapies for urate-lowering drug design (24), PNP was also introduced into our analysis. Finally, the intersected targets and the drug targets together constituted the PF’s candidate targets for HUA.

### 2.4 Establishment of protein-protein interaction network

To systematically clarify the physical interactions and functional associations among candidate targets, the STRING database was implemented to construct a network of protein-protein interaction (PPI) (40). Initially, the candidate targets put into the search box were restricted to “Homo sapiens”. Subsequently, in the basic settings, the “full STRING network” was set as the network type while all active interaction sources were taken into consideration. In addition, the edges refer to “confidence” and the minimum required interaction score was set as “medium confidence (0.400)”. Finally, all candidate targets were divided into three clusters shown in different colors based on the “kmeans clustering” option.

### 2.5 Enrichment analysis of Gene Ontology and Kyoto Encyclopedia of Genes and Genomes

To further elucidate the complex mechanism of PF’s compounds against HUA, Kyoto Encyclopedia of Genes and Genomes (KEGG) pathway enrichment analysis and Gene Ontology (GO) annotation, which consists of three parts [biological processes (BP), cellular components (CC), molecular functions (MF)], were performed based on the candidate targets (41, 42). The Database for Annotation, Visualization and Integrated Discovery (DAVID) (https://david.ncifcrf.gov/) was employed in our analysis (43). On the DAVID website, the candidate target names were imported as “gene list” while “official gene symbol” and “homo sapiens” were selected as identifiers and species, respectively. Finally, all annotated terms obtained following the KEGG and GO enrichment analysis were ranked by *p*-value and then the top 15 KEGG pathways, as well as the top ten of BP, CC, and MF terms were selected for further visualization via an online drawing tool (http://www.bioinformatics.com.cn/).

### 2.6 Molecular docking between Perillae Folium compounds and the candidate targets

Molecular docking, a widely used computational approach that is applied for the prediction of the docking pose and complementarity of a small molecule to the binding sites of macromolecular targets in hit identification as well as lead optimization, was performed (44, 45). As an essential part of docking results, the predicted binding affinity is generally used to evaluate ligand-protein interactions. Typically, lower binding energy values indicate more significant binding modes. In our study, PF compounds’ PDB files were prepared as described above and the candidate proteins’ PDB files were retrieved from the AlphaFold Protein Structure Database (https://alphafold.com/), an artificial intelligence-driven system applied for predicting a protein’s three-dimensional structure (46, 47). The PDB files of both ligands and targets were then converted to PDBQT format via PyRx (v0.8) (48), a software designed for computational drug discovery. Next, molecular docking was performed by an extensively used docking software, AutoDock Vina (v1.1.2) (49). In terms of docking parameter settings, the “maximize” option was selected to ensure the whole protein surface was accessible for ligand docking and hence produced an unbiased prediction of possible interaction sites. Throughout the docking process, “48” was set as the exhaustiveness value. Furthermore, the receptors were treated as fixed structures while full torsional flexibility was allowed for the semi-rigid ligand molecules.

### 2.7 Screening potential compounds for treating hyperuricemia

Given that XDH possessed the highest average binding affinity based on the docking results (**Table 1**), it was chosen for our further analysis. The active sites of XDH were mainly based on the residues identified by a previous study (50) and were modified using the AlphaFold Protein Structure Database, which was applied to predict the structures of the missing segments of the protein. Then Discovery Studio Visualizer 2019, a software widely applied for molecule visualization, was used to display the ligand-residue interactions between the compounds and the predicted binding sites of XDH. The SMILES sequences of the compounds mentioned above were input to ADMETlab 2.0 (https://admetmesh.scbdd.com/) (51) and PAINS-Remover 20 (https://www.cbligand.org/PAINS/) (52) to predict their ADMET properties and check the potential false positives among them, respectively.

**Table 1:** Details of the 25 candidate targets of Perillae Folium treating hyperuricemia.

Target ID	UniProt ID	Target name	Target type	Total binding score (kcal/mol)	Average binding affinity (kcal/mol)
ZH01	P47989	XDH	Intersected/Drug target	-2216.7	-6.76
ZH02	P08254	MMP3	Intersected target	-2151.6	-6.56
ZH03	P21397	MAOA	Intersected target	-2128.7	-6.49
ZH04	P05091	ALDH2	Intersected target	-1975.6	-6.02
ZH05	P45983	MAPK8	Intersected target	-2037.3	-6.21
ZH06	Q14790	CASP8	Intersected target	-1903.8	-5.8
ZH07	P37231	PPARG	Intersected target	-1907.6	-5.82
ZH08	Q04206	RELA	Intersected target	-1752	-5.34
ZH09	P10145	CXCL8	Intersected target	-1634.7	-4.98
ZH10	P01375	TNF	Intersected target	-1709.5	-5.21
ZH11	P40763	STAT3	Intersected target	-1832.7	-5.59
ZH12	P35354	PTGS2	Intersected target	-2061.8	-6.29
ZH13	P00797	REN	Intersected target	-1679.3	-5.12
ZH14	P03372	ESR1	Intersected target	-2029	-6.19
ZH15	P02768	ALB	Intersected target	-2119.5	-6.46
ZH16	P28223	HTR2A	Intersected target	-2062.3	-6.29
ZH17	P15692	VEGFA	Intersected target	-1511.4	-4.61
ZH18	P05231	IL6	Intersected target	-1670.7	-5.09
ZH19	P00491	PNP	Drug target	-1974	-6.02
ZH20	Q96S37	SLC22A12	Drug target	-2020.5	-6.16
ZH21	Q9NSA0	SLC22A11	Drug target	-1998.5	-6.09
ZH22	Q4U2R8	SLC22A6	Drug target	-2064.4	-6.29
ZH23	Q8TCC7	SLC22A8	Drug target	-2110.8	-6.44
ZH24	P33527	ABCC1	Drug target	-2140.9	-6.53
ZH25	Q92887	ABCC2	Drug target	-2031.6	-6.19

ABCC1, ATP-binding cassette sub-family C member 1; ABCC2, ATP-binding cassette sub-family C member 2; ALB, albumin; ALDH2, aldehyde dehydrogenase, mitochondrial; CASP8, caspase-8; CXCL8, chemokine (C-X-C motif) ligand 8; ESR1, estrogen; HTR2A, 5-hydroxytryptamine receptor 2A; IL6, interleukin-6; MAOA, monoamine oxidase type A; MAPK8, mitogen-activated protein kinase 8; MMP3, matrix metalloproteinase-3; PNP, purine nucleoside phosphorylase; PPARG, peroxisome proliferator-activated receptor gamma; PTGS2, prostaglandin G/H synthase 2; RELA, transcription factor p65; REN, renin; SLC22A11, solute carrier family 22 member 11; SLC22A12, solute carrier family 22 member 12; SLC22A6, solute carrier family 22 member 6; SLC22A8, solute carrier family 22 member 8; STAT3, signal transducer and activator of transcription 3; TNF, tumor necrosis factor; VEGFA, vascular endothelial growth factor A; XDH, xanthine dehydrogenase.

### 2.8 Chemical structure analysis of Perillae Folium compounds

To explore the associations between the structures of PF’s compounds and their interactions with XDH, OSIRIS DataWarrior (v5.5.0), a software used for analysis and visualization of chemical data, was implemented for building a cluster network based on the structural similarity and binding energy (53). Subsequently, ClassyFire, an application for chemical classification (http://classyfire.wishartlab.com/), was employed to determine the classifications of compounds in the clusters that contain more than seven components (54), adhering to the method that we used in our previously published work (55). Furthermore, we compared the structure profiles of five compounds (identified in the active sites of XDH) and all compounds with two well-studied inhibitors of XDH: febuxostat and allopurinol.

### 2.9 Construction of a compound-target network for hyperuricemia

To visualize the interactions between active ligands and receptors, the top ten compounds with the highest binding energy to each candidate target, which indicate potential physiological importance, were included to build a compound-target network. Cytoscape (v3.9.1), a software aimed at visualizing complex networks, was implemented for the establishment of the network. In the network, the degree value indicates how many targets a compound was predicted to strongly bind with. The color and size of rectangle nodes were determined by the corresponding degree value of each included compound.

### 2.10 Molecular dynamics simulations for potential inhibitors against hyperuricemia

MD simulations were used as a more rigorous screening tool for identifying and evaluating candidate compounds from PF for HUA. Here, the MD simulation module hosted by SiBioLead (https://sibiolead.com/) (56) was employed. This interactive module can assist users in pre-processing the protein-ligand complexes, energy minimizing the solvated systems, conducting temperature and pressure equilibration runs, performing the final production runs, and generating results to aid analysis. The PDB files of the *apo*-protein and ligand-protein complexes were uploaded onto the SiBioLead server. The ligand-protein complexes include compounds designated ZS025, ZS056, ZS090, ZS093, and ZS252, as identified from molecular docking based on docking scores, binding poses, and the presence of hydrogen bond interactions. MD simulations of the *apo*- and ligand-bound complexes were performed with GROMACS (57) using the OPLS all-atom force field (58). The ligand topology is generated with AMBERTOOLS (59) and ACPYPE (60) based on an automated process within SiBioLead. The simulation systems were prepared by immersing the *apo*- and ligand-protein complexes in a triclinic box and solvated with the simple point charge water model. An equal number of Na^+^ and Cl^-^ counterions at a concentration of 150 mM was added to neutralize the charges within the solvated system. Energy minimization was performed using the steepest descent integrator with a maximum of 5,000 steps. During the equilibration runs, the temperature was maintained at 300 K while the pressure was kept constant at 1 bar for a duration of 100 ps. After equilibration, the system was subjected to a production run using the leapfrog integrator for 50 ns. The trajectory generated from the production runs was analyzed within the Sibiolead module. Analyses applied in this study include the root mean square deviation of backbone Cɑ atoms (RMSD), root mean square fluctuation (RMSF), the radius of gyration (Rg), solvent accessible surface area (SASA), the number of hydrogen bonds (Hbnum), and the number of atom pairs within 0.35 nm (nPairs) between the protein and ligand throughout the 50 ns simulation. To visualize the physical behavior of small molecules in the pocket of the target, Visual Molecular Dynamics software, a tool developed for modeling, visualization, and analysis of biological systems, was implemented (61).

## 3 Results

### 3.1 Herbal compounds and potential targets of Perillae Folium for treating hyperuricemia

**Figure 2:**
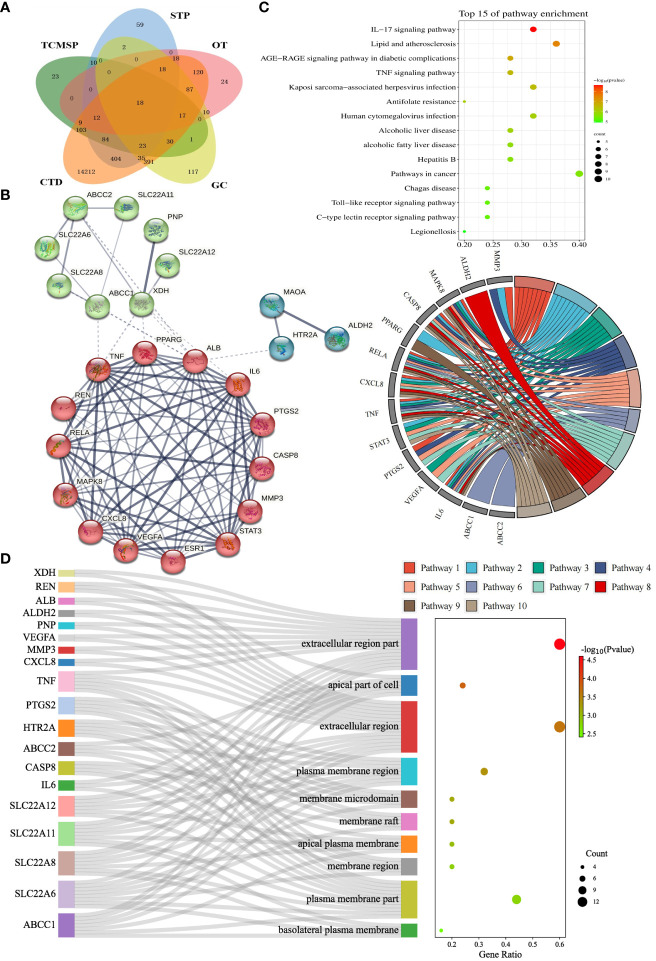
Identification and enrichment analysis of candidate targets for Perillae Folium treating hyperuricemia. **(A)** Venn diagram of Perillae Folium-related targets and hyperuricemia-related targets. TCMSP, the traditional Chinese medicine systems pharmacology database; STP, the SwissTarget Prediction database. CTD, the CTD database; OT, the Open Targets database; GC, the GeneCards database. **(B)** Protein-protein interaction network of the candidate targets of PF treating HUA. Nodes stand for proteins; edges stand for the protein-protein associations, and the thickness of the edges indicates the strength of the evidence supporting protein-protein interactions; **(C)** The KEGG enrichment analysis of the candidate targets of PF treating HUA. The bubble chart on the top right displayed the top 15 KEGG pathways. The chord diagram on the center-right consists of gene names (left semicircle) and pathway names (right semicircle). Pathways 1-10 stand for IL-17 signaling pathway, lipid and atherosclerosis, AGE-RAGE signaling pathway in diabetic complications, TNF signaling pathway, Kaposi sarcoma-associated herpesvirus infection, antifolate resistance, human cytomegalovirus infection, alcoholic liver disease, non-alcoholic fatty liver disease, and hepatitis B, respectively. KEGG, Kyoto Encyclopedia of Genes and Genomes. **(D)** The GO enrichment analysis of the candidate targets of PF treating HUA. The Sankey diagram of CC analysis is composed of gene names, CC terms, and a bubble plot. GO, Gene Ontology; CC, cellular component.

We collected details of a total of 328 compounds of PF from the TCMSP database (**Supplementary Table S1**). As for the candidate targets, we identified 683 targets from SwissTargetPrediction, 330 from TCMSP, 749 from GeneCards, 333 from Open Targets Platform, and 15581 from the CTD database (**Figure 2A**). Next, targets common across all of the above databases were identified, and 18 targets were obtained, including xanthine dehydrogenase (XDH), matrix metalloproteinase-3 (MMP3), monoamine oxidase type A (MAOA), aldehyde dehydrogenase, mitochondrial (ALDH2), mitogen-activated protein kinase 8 (MAPK8), caspase-8 (CASP8), peroxisome proliferator-activated receptor gamma (PPARG), transcription factor p65 (RELA), chemokine (C-X-C motif) ligand 8 (CXCL8), tumor necrosis factor (TNF), signal transducer and activator of transcription 3 (STAT3), prostaglandin G/H synthase 2 (PTGS2), renin (REN), estrogen (ESR1), albumin (ALB), 5-hydroxytryptamine receptor 2A (HTR2A), vascular endothelial growth factor A (VEGFA), and interleukin-6 (IL6) (**Figure 2A**). In addition, to comprehensively investigate the interactions between PF’s ingredients and HUA, we paid special attention to the drug targets for HUA, including purine nucleoside phosphorylase (PNP), solute carrier family 22 member 12 (SLC22A12), solute carrier family 22 member 11 (SLC22A11), solute carrier family 22 member 6 (SLC22A6), solute carrier family 22 member 8 (SLC22A8), ATP-binding cassette sub-family C member 1 (ABCC1), and ATP-binding cassette sub-family C member 2 (ABCC2). Interestingly, each of the selected drug targets was predicted to have a good binding affinity (less than -6 kcal/mol) (62) with PF compounds in the molecular docking results later (**Table 1**), which suggests that a large number of PF compounds possess good binding property with the drug targets. Subsequently, we selected 18 targets that are common across the databases, and seven potential drug targets, which were incorporated into our further study (**Table 1**). Next, a PPI network was built based on the 25 candidate targets in the STRING database (**Figure 2B**). As illustrated in the network, the targets are clustered into three groups colored red, green, and blue. The network has 102 edges between 25 nodes and each node is associated with at least one other node. Additionally, the average node degree is 8.16 while the average local clustering coefficient is 0.664. Furthermore, the PPI enrichment *p*-value is less than 1.0e^-16^.

### 3.2 Perillae Folium’s involvement in multiple signaling pathways

To elucidate the possible mechanism of compound-target interactions, GO and KEGG enrichment analyses were performed. In the KEGG pathway enrichment analysis, the IL-17 signaling pathway showed the lowest *p*-value, which was composed of IL6, MAPK8, CXCL8, CASP8, MMP3, PTGS2, TNF, and RELA. Additionally, out of the 15 pathways, pathways in cancer had the largest gene ratio (**Figure 2C**). In the GO analysis, the 25 potential targets were annotated by BP, CC, and MF analysis, and corresponding terms were ranked from smallest to largest value based on *p*-value (**Figure 2D**, **Supplementary Figure S1**). Based on CC analysis (**Figure 2D**), the extracellular region part, apical part of the cell, extracellular region, plasma membrane region, membrane raft, membrane microdomain, apical plasma membrane, membrane region, plasma membrane part, basolateral, and plasma membrane, were identified as the top ten categories. Among them, the components with the largest enriched protein number were the extracellular region part and extracellular region, while the former was also the most significant one. As shown in **Supplementary Figure S1**, the MF analysis revealed that the dominant functions of the potential targets included organic acid transmembrane transporter activity, identical protein binding, organic anion transmembrane transporter activity, cytokine receptor binding, anion transmembrane transporter activity, carboxylic acid transmembrane transporter activity, prostaglandin transmembrane transporter activity, eicosanoid transmembrane transporter activity, inorganic anion exchanger activity, and anion binding. Under the BP category (**Supplementary Figure S1**), the top ten pathways were shown as follows: response to oxygen-containing compound, response to nitrogen compound, response to chemical, response to organonitrogen compound, response to organic substance, response to drug, response to endogenous stimulus, lipid localization, cellular response to oxygen-containing compound, and fatty acid transport. Furthermore, the GO term, organic acid transmembrane transporter activity, has the most significant result, and the largest number of proteins were annotated in the “identical protein binding”.

### 3.3 Evaluation of Perillae Folium compounds docking with hyperuricemia proteins

To explore whether PF compounds are biologically connected with potential targets of HUA based on their corresponding molecular structures, 328 constituents were docked with 25 proteins, producing 8200 docking results, which range from -11.7 to -2.1 kcal/mol. Typically, lower binding energy indicates higher binding affinity, which implies that a ligand tends to combine with a receptor more tightly. From the perspective of PF compounds, there are 139 ligands (approximately 43.3%) whose binding energy is lower than -6 kcal/mol, which is presently defined as having a strong interaction (**Supplementary Table S2**). Among them, the top ten compounds with the highest average binding scores included ZS059, ZS058, ZS014, ZS070, ZS074, ZS083, ZS118, ZS066, ZS068, and ZS055. A total of 27 ligands were found to possess lower binding energy than febuxostat (-8.7 kcal/mol). Moreover, 16 constituents were found to dock well with each of 25 proteins (binding energy < -6 kcal/mol), which have prospects as potential “pan-acting compounds” against HUA, such as ZS014, ZS070, ZS083, ZS118, and ZS066 (**Supplementary Table S2**). In addition, there exist 39 compound-target pairs with exceptionally high binding affinity (< 10 kcal/mol) (**Supplementary Table S2**).

**Figure 3:**
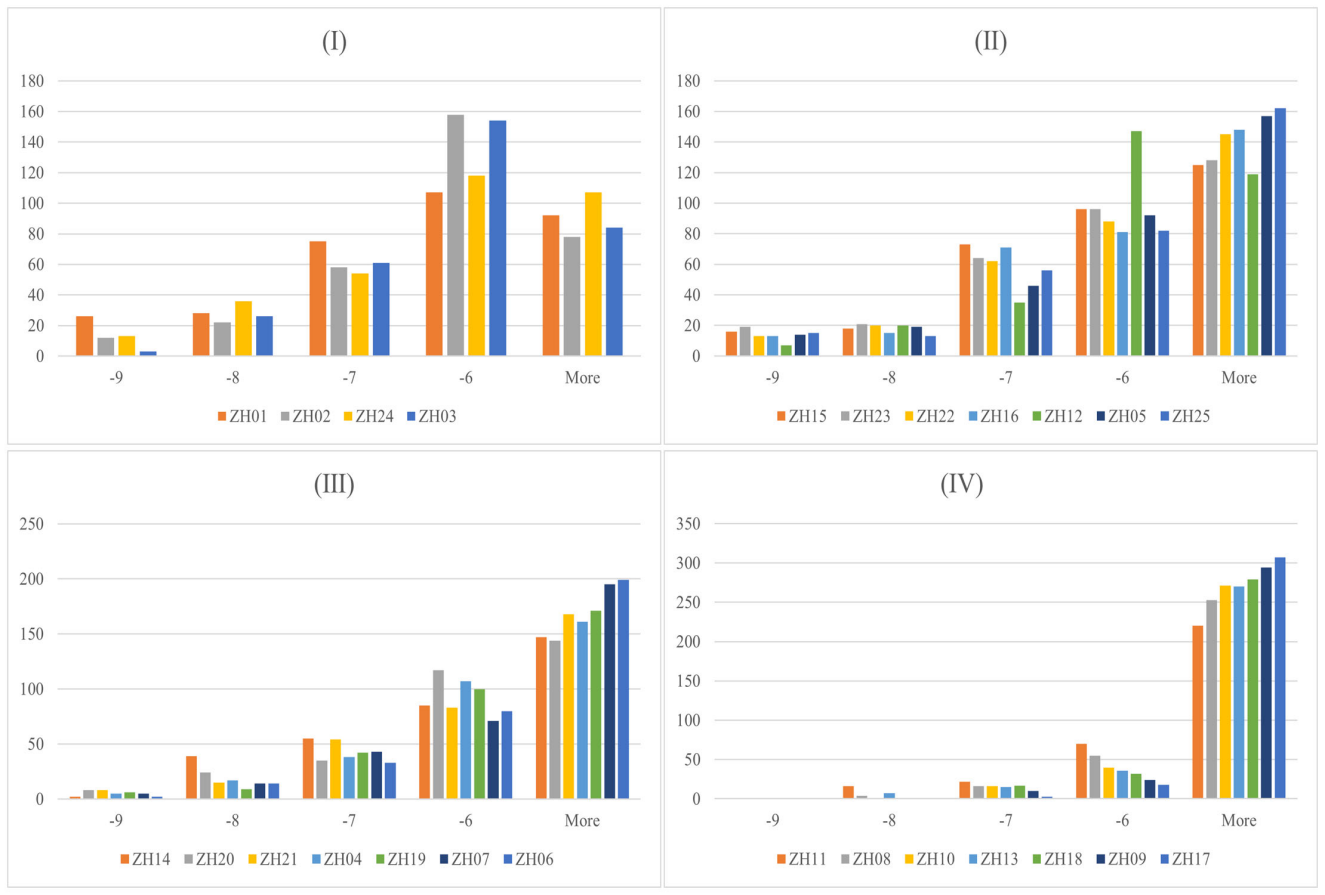
Docking results between 328 Perillae Folium compounds and the 25 candidate targets for treating hyperuricemia. Histograms for 328 compounds against the 25 targets; the *x*-axis stands for the binding energy intervals while the *y*-axis represents the number of compounds. Group (I) Top 1-4 targets; Group (II) Top 5-11 targets; Group (III) Top 12-18 targets; Group (IV) Top 19-25 targets. The proteins belonging to which group are determined by the average binding affinity of the targets. The corresponding protein names of 25 target IDs refer to **Table 1**.

Docking results and features between each target and all ligands are summarized in **Figure 3** and **Table 1**. As shown in **Table 1**, the average binding scores ranged from -6.76 to -4.61 kcal/mol. The top 10 targets with the best binding scores were ZH01 (XDH), ZH02 (MMP3), ZH24 (ABCC1), ZH03 (MAOA), ZH15 (ALB), ZH23 (SLC22A8), ZH22 (SLC22A6), ZH16 (HTR2A), ZH12 (PTGS2), and ZH05 (MAPK8). Among them, XDH had the lowest average binding energy (-6.76 kcal/mol), which indicated that XDH bound well with the 328 compounds of PF. In addition, histograms were also drawn to display the distribution characteristics of the 25 targets’ docking results. In the histograms, the 25 targets were ranked by their average binding affinity and subsequently were divided into four groups (**Figure 3**). Specifically, Group (I) corresponded to the top one to four targets while the remaining 21 targets were evenly separated into three groups, Group (II), Group (III), and Group (IV). As demonstrated in **Figure 3**, there is at least one ligand predicted to bind with high affinity (binding scores ≤ -9 kcal/mol) with targets from Group (I) to (III), and more than 20 ligands had a similar effect on ZH01 (the only one). Furthermore, most compounds (more than 50%) docked with targets from Group (I) and Group (II) with an intermittent binding affinity (-9 to -6 kcal/mol). On the contrary, most compounds had a weak binding affinity (> -6 kcal/mol) with targets of Group (III) and Group (IV), especially for ZH09 and ZH17 whose numbers of bound ligands were both approximately 300 (91.4%).

### 3.4 Compound-target network revealed potential multi-target multi-active compound mechanisms

**Figure 4:**
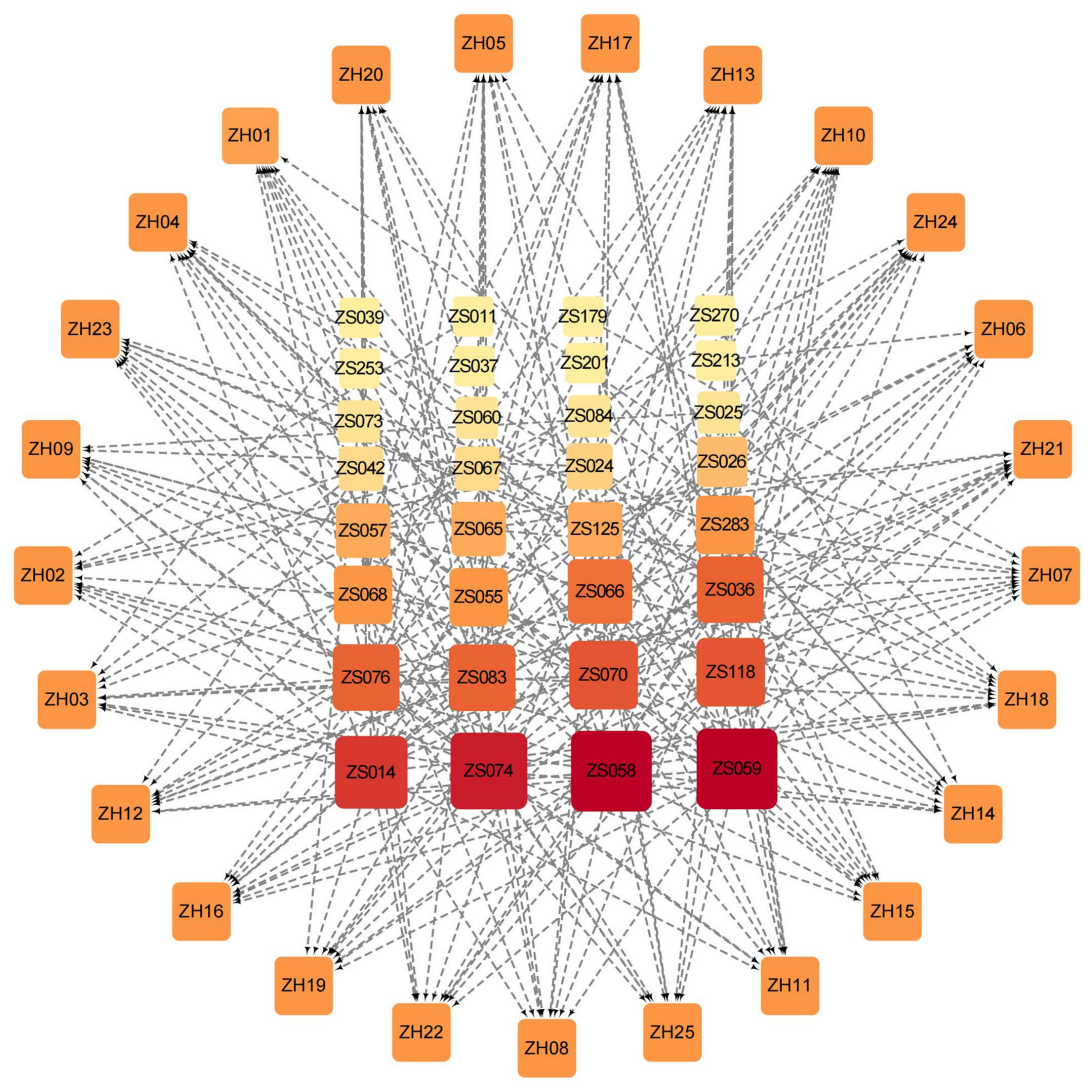
Compound-target network based on the top 10 active Perillae Folium compounds for each target and all 25 targets. Nodes in the outer layer represent the 25 targets while the inner are the active compounds. The darker color and larger size of the inner nodes denote the higher degree value in the network. The gray edges connect each protein and its corresponding top 10 compounds.

To comprehensively demonstrate the interactions between compounds and targets, we established an active compound-target network. The top 10 active compounds for each target were selected based on their binding energy. After removing the duplicate compounds, a total of 32 active compounds were preserved to build the network (**Figure 4**). In the network, the nodes arranged in the outer layer represent the targets while the inner layer represents the compounds. The darker color of the inner nodes denotes the higher degree value, which typically indicates a node in the network with more connections with other nodes. Of the 32 active compounds, 24 of them showed good binding ability to more than one target. Interestingly, 10 compounds bound well with more than half of the 25 targets (**Supplementary Table S3**). Notably, the degree values of both ZS059 and ZS058 are 21. The above findings may highlight the possible synergistic effects of PF against HUA characterized by a multi-target and multi-component mechanism.

### 3.5 Cluster analysis based on the structures of the 328 compounds and their affinity to XDH

**Figure 5:**
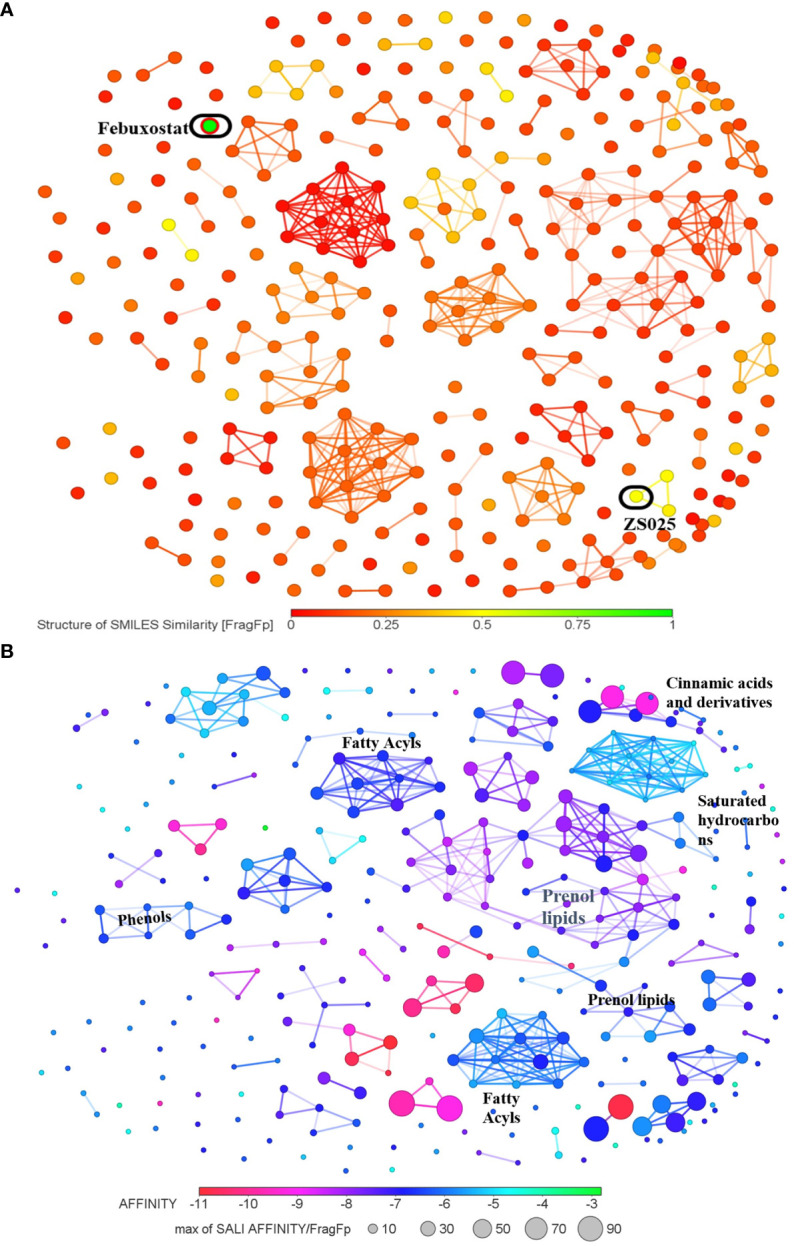
The cluster network of Perillae Folium compounds based on structural similarity. **(A)** The visualization of similarity scores of 328 compounds from Perillae Folium compared with febuxostat. Green nodes represent febuxostat, while other nodes stand for compounds of Perillae Folium. **(B)** The cluster network of 328 Perillae Folium compounds based on binding affinity and structural similarity. Colors of nodes stand for the binding affinity of PF compounds to XDH while the size of nodes is determined by a formula according to binding energy and similarity. A larger node implicates a lower binding affinity and a higher similarity score with the vicinal compounds.

As the structures of small molecules determine their properties and biological function (55), we subsequently conducted a structure-based analysis for the 328 PF compounds. Given the essential role XDH plays in the urate-lowering agents’ development and the observation that XDH possesses the greatest average binding energy in our analysis described above (**Table 1**), we compared the 328 PF compounds with the XDH inhibitors, allopurinol and febuxostat. As illustrated in **Supplementary Figure S2** and **Figure 5A**, most PF compounds show weak similarity with both inhibitors. Among them, compounds with the top five similarity scores with febuxostat were identified, including ZS025, ZS024, ZS031, ZS134, and ZS084 (**Supplementary Table S4**).

Next, we visualized the correlation between the structures of the 328 PF compounds and their potential biological effect on XDH. In **Figure 5B**, 328 compounds are presented as nodes in various colors according to their corresponding binding energy. Compounds with similar structures were automatically gathered and many ensuing clusters were generated. For each cluster, a node with a color closer to red represents higher binding affinity while a node with a larger size indicates a greater similarity with the surrounding compounds. As presented in **Figure 5B**, **Supplementary Figure S3**, and **Supplementary Table S5**, seven clusters containing at least seven nodes were identified, which were classified as prenol lipids, saturated hydrocarbons, fatty acyls, cinnamic acids and derivatives, and phenols. Among them, the largest cluster is composed of 37 compounds, whose classes and subclasses were mainly identified as prenol lipids and sesquiterpenoids, respectively. It is worth noting that nodes in the largest cluster tend to appear in purple-red, which suggests that they commonly possess good binding affinity (**Figure 5B**).

### 3.6 Five ligands that strongly interact with XDH are drug-like and non-toxic molecules

To further explore which components are more likely to be potential inhibitors for HUA, some parameters including pan assay interference compounds (PAINS), hydrogen bonds (H-bonds), binding affinity, and ADMET properties were taken into consideration. Since XDH, a widely recognized drug target for HUA, bound to PF compounds with the highest binding affinity (-6.76 kcal/mol) among the 25 targets, it was considered for further analysis. Next, febuxostat, the known inhibitor of XDH, was selected as a positive control. Four active site residues (50) that febuxostat binds to were modified using AlphaFold, including Asn769, Glu803, Arg881, and Thr1011. Subsequently, 37 compounds that were identified as being close to the four residues mentioned above based on docking results were found and selected for further analysis, which implied that these compounds may bear similar activity against XDH as febuxostat (**Supplementary Table S6**). At the beginning of the compound screening, with the help of PAINS-Remover, we performed PAINS tests for all compounds, which is commonly applied to removing false positives in high-throughput screens. Importantly, all compounds successfully passed the test. Subsequently, we focused on the formation of H-bonds between ligands and targets, which is typically considered to play a critical role in enzyme–inhibitor binding (63). Of the 37 ligands, only ZS136 formed an H-bond with Thr1011, one of the four key residues. However, among the 37 ligands, we identified 22 compounds that formed conventional H-bonds and six of them formed probable H-bonds (unfavorable donor-donor bonds) with other residues near the four key active site residues. Additionally, since understanding the ADMET properties of candidate compounds is essential at the beginning of drug discovery (64), we predicted the properties of 37 compounds via the ADMET lab 2.0 database. Given that the well-known law, Lipinski Rule of Five, was widely applied for evaluating the drug-like properties of small molecules (65) while human hepatotoxicity plays a considerable role in drug development (66, 67), these two key parameters were taken into account and considered as bearing high importance. All compounds passed Lipinski’s rule, while three compounds, ZS018, ZS044, and ZS116, were predicted to have poor performance in terms of hepatoxicity. Based on the previous consideration, the inclusion criteria were established as follows: (1) 0 alerts in the PAINS test; (2) forming at least one H-bond with residues near ASN769, GLU803, ARG881, and THR101; (3) binding energy < -6 kcal/mol; (4) 0 violation in the Lipinski Rule of Five; (5) H-HT value ≤ 0.7. After the screening, five qualified compounds were identified, including ZS025, ZS056, ZS090, ZS093, and ZS252 (**Table 2**).

**Table 2:** Details of the five potential inhibitors meet the demand of the inclusion criteria.

Compound codes	Compound names	Identified by mass spectrometer	Conventional H-bond formed at active sites	Unfavorable donor-donor bonds	Binding affinity (kcal/mol)	H-HT	PAINS	Lipinski rules of five
ZS025	Scutellarein	Yes	2(Ala1080, Lys1046)	0	-7.85	0.086	0 alert	Pass
ZS056	Benzyl alpha-D-mannopyranoside	No	2(Gln768, Gln1195)	0	-6.86	0.044	0 alert	Pass
ZS090	Elemol	No	1(Ser1081)	0	-6.30	0.082	0 alert	Pass
ZS093	Diisobutyl phthalate	No	2(Ser1081, Gln1041)	1(Ser1081)	-6.40	0.008	0 alert	Pass
ZS252	(3R)-Hydroxy-beta-ionone	No	2(Arg913, Gln1195)	1(Ser1081)	-6.42	0.311	0 alert	Pass

H-bond, hydrogen bond; H-HT, human hepatotoxicity; PAINS, pan assay interference compounds.

**Figure 6:**
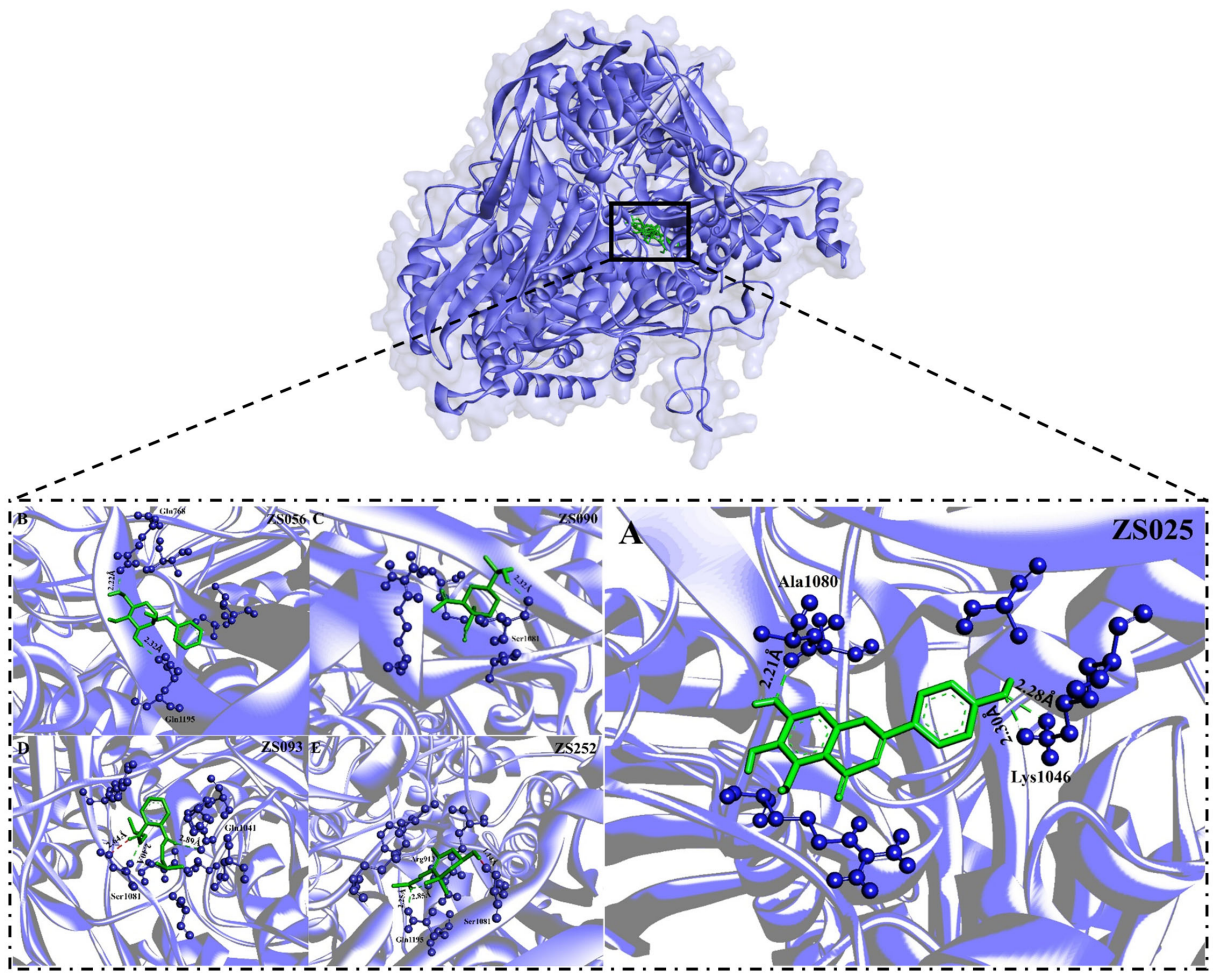
The binding modes of five potential inhibitors identified from Perillae Folium with XDH. The purple substance represents XDH while the green one stands for a potential inhibitor. Each inhibitor interacts with the amino acid of XDH via H-bonds (green) or unfavorable donor-donor bonds (red). The number in Å above the bond indicates the distance of the bond. The upper layer of the diagram indicates all of the five potential inhibitors bind to the active sites of the XDH, including **(A)** ZS025 (scutellarein); **(B)** ZS056 (Benzyl alpha-D-mannopyranoside); **(C)** ZS090 (Elemol); **(D)** ZS093 (Diisobutyl phthalate); **(E)** ZS252 [(3R)-hydroxy-beta-ionone] in the lower layer.

Subsequently, we visualized the binding modes between the five identified compounds and XDH. As per **Figure 6**, ZS090 formed one conventional H-bond with the active site of XDH, while ZS025, ZS056, and ZS093 all formed two in the same region. Among them, ZS090 was found to bind with Ser1081 at a distance of 2.32 Å while ZS025 bound with Ala1080 and Lys1046 with a distance of 2.21 Å, 2.28 Å, and 2.30 Å, respectively. Similarly, ZS056 bound to Gln768 and Gln1195 at a distance of 2.22 Å and 2.32 Å, separately, while ZS093 interacted with active site residues, Ser1081 and Gln1041 at a distance of 1.64 Å, 2.40 Å, and 2.89 Å, respectively. In addition to forming H-bonds at the two active site residues (Gln1195 and Arg913), ZS252 also interacted with Ser1081 via unfavorable donor-donor bonds with a distance of 2.25 Å, 2.85 Å, and 1.14 Å, separately.

### 3.7 Molecular dynamics simulations of the five potential inhibitors

**Figure 7:**
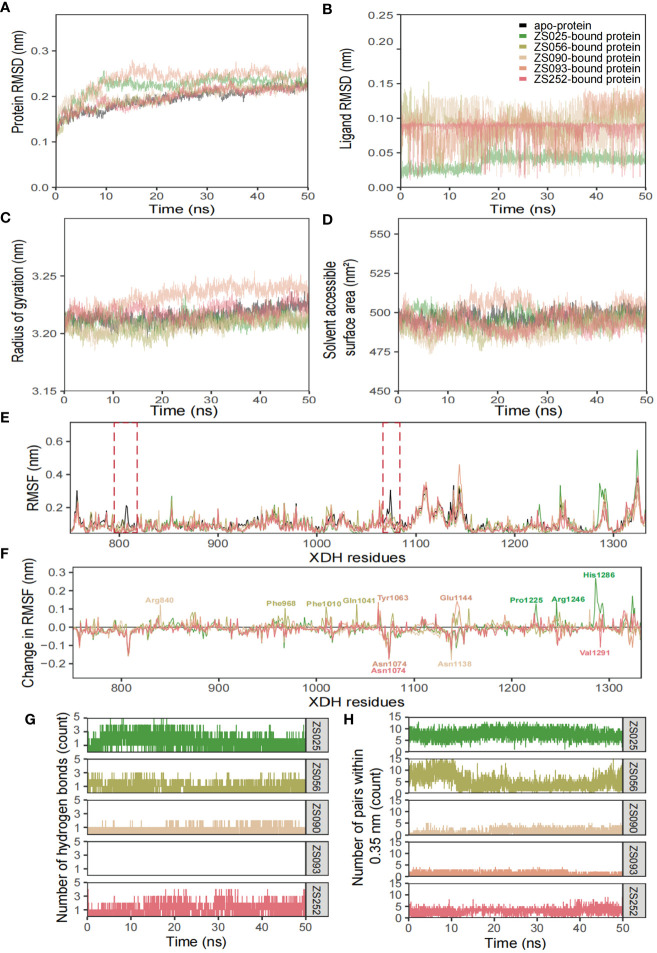
Structural analyses of the *apo*-, ZS025-, ZS056-, ZS090-, ZS093-, and ZS252-bound XDH based on molecular dynamics simulation. The five inhibitors’ values of some parameters are illustrated as follows, including protein RMSD **(A)**, ligand RMSD **(B)**, Rg **(C)**, SASA **(D)**, RMSF **(E)**, change in RMSF **(F)**, the number of hydrogen bonds **(G)**, and the number of pairs within 0.35 mm **(H)**.

The conformational stability and flexibility of the six simulated systems were analyzed using structural and dynamical quantities, including RMSD, RMSF, Rg, SASA, and the number of hydrogen bonds as described in **Figure 7**. As observed in **Figure 7A**, the protein-ligand complexes achieved equilibration at approximately 10 ns following system relaxation, evidenced by the formation of a plateau in the curve after this time. **Table 3** provides a statistical summary of averaged trajectory outcomes from 10-50 ns after the systems have equilibrated. RMSD calculations for the ligand-free and ligand-bound proteins are visualized in **Figure 7A**. It is observed that all systems were relatively stable throughout the 50 ns with no major displacements above 0.25 nm. Small fluctuations after approximately 10 ns suggest that all systems have attained equilibration. The average RMSD of backbone Cα atoms for the *apo*-protein was 0.203 nm, followed closely were the ZS252-bound protein at 0.204 nm. Although the trends and magnitude of RMSD in the ZS252-bound protein closely resemble that of the *apo*-protein, the ligand itself (ZS252) had a relatively higher RMSD. The RMSD of ZS252 averaged at 0.086 nm, a value comparable to other ligands ZS056, ZS090, and ZS093. Interestingly, the average RMSD of ZS025 appeared the lowest among all five ligands, averaging at 0.04 nm. This suggests a greater stability of ZS025, which largely retains its initial structure, compared to the rest of the ligands at the XDH active site (**Figure 7B**).

**Table 3:** Structural analyses of stabilized trajectories following equilibration at 10 ns.

Analysis	XDH-*apo*	XDH -ZS025	XDH -ZS056	XDH -ZS090	XDH -ZS093	XDH -ZS252
Protein RMSD (nm)	0.203 ± 0.014	0.231 ± 0.009	0.207 ± 0.015	0.208 ± 0.013	0.247 ± 0.01	0.204 ± 0.016
Ligand RMSD (nm)	N/A	0.04 ± 0.008	0.087 ± 0.02	0.088 ± 0.025	0.092 ± 0.025	0.086 ± 0.012
Radius of gyration (nm)	3.216 ± 0.006	3.212 ± 0.005	3.207 ± 0.007	3.21 ± 0.005	3.236 ± 0.006	3.219 ± 0.006
Solvent accessible surface area (nm^2^)	497.8 ± 3.969	495.465 ± 4.137	490.625 ± 4.593	490.129 ± 4.775	503.408 ± 4.947	492.797 ± 4.726
RMSF (nm)	0.113 ± 0.056	0.11 ± 0.067	0.112 ± 0.064	0.108 ± 0.052	0.111 ± 0.065	0.114 ± 0.061
Number of hydrogen bonds	N/A	5687 ± 1.044	3155 ± 0.708	2104 ± 0.659	0 ± 0	4864 ± 0.788
Number of pairs within 0.35 nm	N/A	31011 ± 1.957	15082 ± 2.467	4854 ± 1.161	3791 ± 0.943	12550 ± 1.243

N/A, not applicable; RMSD, root mean square deviation of backbone Cα atoms; RMSF, root mean square fluctuation; XDH, xanthine dehydrogenase; ZS025, scutellarein; ZS056, benzyl alpha-D-mannopyranoside; ZS090, elemol; ZS093, diisobutyl phthalate; ZS252, (3R)-hydroxy-beta-ionone.

For the Rg and SASA, the changes in protein conformation over time measured by the Rg are shown in **Figure 7C** while the changes in SASA are available in **Figure 7D**. The absence of major protein conformational changes among all except for one of the systems was reflected by the relatively narrow standard deviation of less than 0.01 nm, suggesting high conformational stability and compactness of the protein. The ZS093-bound protein had the highest average Rg value of 3.236 nm, with more remarkable unfolding appearing after 20 ns of simulation. Consistent with the findings from Rg calculations, the increase in SASA values for the ZS093-bound protein was also noticeable. This increase began at an earlier timeframe, at approximately 10 ns, and fell just before the 30 ns mark and may indicate an expansion or destabilization of the closed protein conformation upon binding to ZS093. Intriguingly, there was a small difference in average SASA values between two ligands (ZS056, ZS090) and the *apo*-protein of up to 0.09 nm^2^. This reduction in SASA upon binding with ZS056 and ZS090 may suggest that the ligand-bound structure induced a more compact closed-conformation XDH structure, supporting the results from the Rg calculation shown above.

RMSF and changes in RMSF calculations were focused on binding-site residues between Thr750 to Val1333, as shown in **Figures 7E, F**. The overall inspection of site-specific residues of the ligand-bound and ligand-free structures suggests that they exhibit similar flexibility. Most of the peaks were located around loop regions and highly overlapped with each other. Additionally, the troughs also follow a similar trend, particularly for the ligand-bound structures. Minor differences around the two regions were observed in the average RMSF values between the *apo*- and ligand-bound structures, surrounded by the peaks of Gly801 and Asn1074 residues, as highlighted in red dotted boxes in **Figure 7E**. The significant suppression of residue fluctuation in these regions observed in all ligand-bound structures suggests that this area could be important for ligand binding and the subsequent stability of the XDH protein structure. Consistently, the changes in average RMSF values among ligand-bound structures followed a trend, except for some regions bound to ZS025 (Pro1225, Arg1246, His1286), ZS056 (Phe968, Phe1010, Gln1041), ZS252 (Asn1074, Val1291), ZS090 (Arg840, Asn1138) and ZS093 (Tyr1063, Asn1074, Glu1144). Mutagenic studies may provide valuable insights into the biological significance of these residues and confirm their roles in the binding of the currently proposed candidate ligands.

**Figures 7G, H** depict the number of hydrogen bonds and pairs within 0.35 nm, respectively. It is observed that ZS025 maintained a high number of hydrogen bonds throughout the 50 ns simulation, followed by ZS252 and ZS056. As for the ZS025 ligand, this molecule gained polar contacts with XDH residues as the system approached equilibration and maintained a high number of contacts until approximately 20 ns before losing a few contacts towards the second half of the simulation. This observation could be attributed to the change in ligand conformation, consistent with the ligand RMSD illustrated in **Figure 7B**. Although polar contacts were undetected between the ZS093 and the XDH structure, other predominant hydrophobic interactions may be responsible for the binding stability of ZS093, albeit resulting in a significantly weaker connection.

### 3.8 Scutellarein is proven to be a potential inhibitor against XDH based on literature-mining and untargeted metabolomics

**Figure 8:**
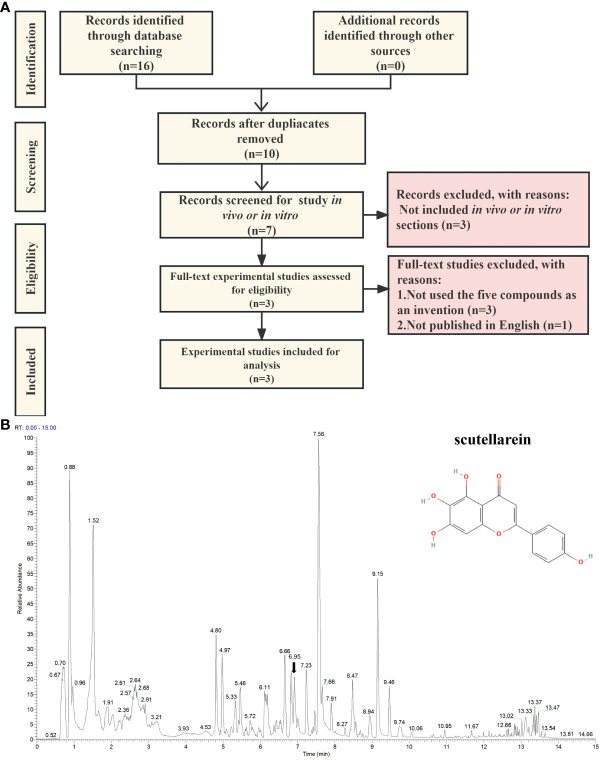
The validation of scutellarein through text-mining and untargeted metabolomics. **(A)** The flowchart of text-mining. **(B)** The mass spectrum of the Perillae Folium granule mixture in positive ion mode. The *x*-axis stands for retention time (min), which ranges from 0 to 15 minutes. The *y*-axis represents the signal intensity. Each peak indicates a unique compound detected in the Perillae Folium. The black arrow indicates the inhibitor we focus on, whose schematic diagram is on the top right.

To find laboratory evidence about the biological effects that the five compounds exert on uric acid *in vitro* and *in vivo*, we carried out a text-mining analysis based on PubMed, Scopus, and CINAHL. The records we searched for were from the earliest available records to 28^th^ June 2023. As per the search strategy shown in **Supplementary Table S7**, the keywords we added to the query box included scutellarein, benzyl alpha-D-mannopyranoside, diisobutyl phthalate, Elemol, Diisobutyl phthalate, (3R)-hydroxy-beta-ionone, hyperuricemia, uric acid, xanthine oxidase, and xanthine dehydrogenase. We considered all available English language *in vivo* and *in vitro* experimental studies of the five potential compounds. We included the records of the five inhibitors that were used in the invention. The exclusion criteria were met in these conditions: (1) the papers were published in review type; (2) the papers were not involved in the selected compounds targeting XO and XDH or treating HUA and uric acid; (3) the paper was published in language other than English. As shown in **Figure 8A**, through an extensive search, we identified two records in PubMed, ten records in Scopus, and four in CINAHL. After removing the duplicate results, ten of them remained for further analysis. Subsequently, three papers published in the review, one in a language other than English, and three without any of the five compounds as interventions, were excluded (**Supplementary Table S8**). After exclusions based on the above-mentioned criteria, three eligible studies were included (**Supplementary Table S9**). Interestingly, scutellarein was reported in all four published articles. The results of the papers were mainly based on *in vitro* studies and the conducted studies were primarily xanthine oxidase inhibitory activity assays. As per **Supplementary Table S9**, compared with allopurinol, which was chosen as a positive control drug, scutellarein showed good or modest inhibitory effects which varied from study to study. In addition, previous research has also determined scutellarein’s content in PF (2.25 ± 0.49 mg/g) (68). However, except for scutellarein, the remaining four compounds were not reported in our identified study. In addition, we also compared the structures of the five inhibitors with febuxostat and the results showed that ZS025 possesses the top similarity score, which is 0.50877 and appears in light yellow (**Supplementary Table S4**, **Supplementary Figure S4**). This finding is also consistent with the result described above (**Figure 5A**).

Based on the above findings, we focused on ZS025 in our further study. Subsequently, to confirm the presence of ZS025 in the mixture of PF granules, we performed an untargeted metabolomics analysis with UPLC-ESI-Q-Orbitrap-MS technology. As a result, there were 543 molecules identified in the mass spectrometry results. Of the 543 molecules, 22 were coincident with those retrieved from the TCMSP database, such as rosmarinic acid, scutellarein, esculetin, luteolin 7-*O*-beta-D-glucoside, and luteolin. Among them, scutellarein, one of the five potential inhibitors that passed our screening protocol, was acquired in positive ion mode, whose mass-to-charge ratio (m/z) is 287.06 and retention time (RT) is 6.95 min, has a peak area of 5686912512, which was ranked second in the 22 overlapping molecules. However, except for scutellarein, the remaining four compounds were not detected in the mixture (**Supplementary Table S10**, **Figures 8B** and **Supplementary Figure S5**).

### 3.9 Molecular dynamics simulation of scutellarein-XDH complex for 200 ns

**Figure 9:**
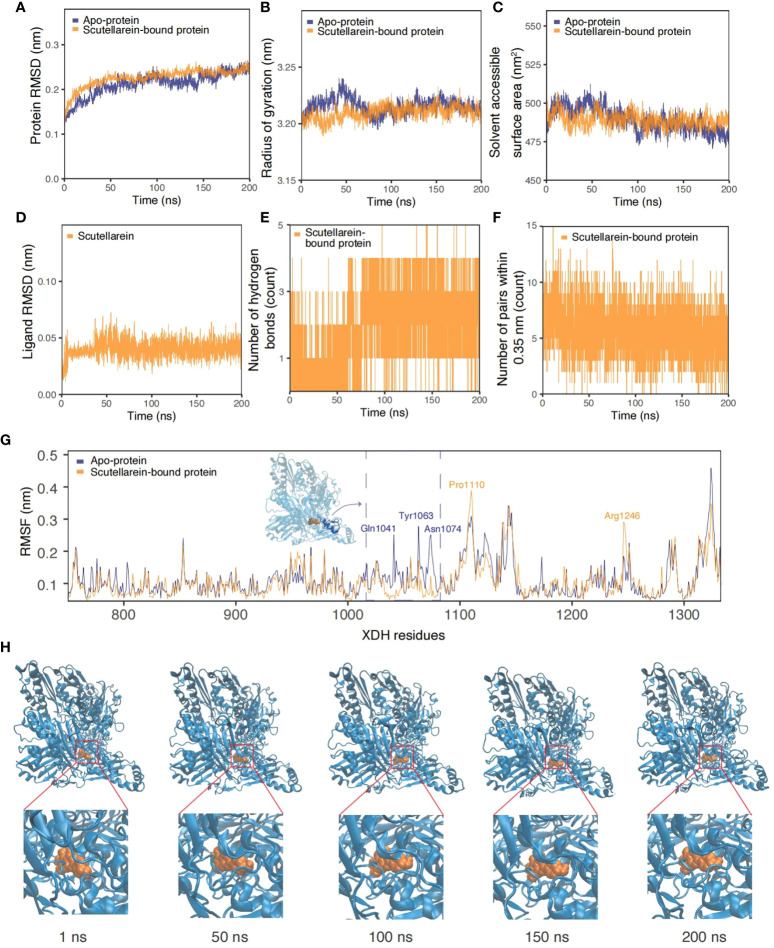
Time-series analysis from the MDS trajectories of the apo-protein (blue) and scutellarein-bound protein (orange). **(A)** Protein backbone RMSD (nm), **(B)** Radius of gyration (nm), **(C)** Solvent accessible surface area (nm^2^), **(D)** Ligand scutellarein RMSD (nm), **(E)** Number of hydrogen bonds, **(F)** Number of pairs within 0.35 nm, **(G)** Per-residue RMSF (nm) highlighting the residues with the highest fluctuation and an accompanying inset of the protein structure, **(H)** Snapshots of the simulated trajectory of the protein (blue cartoon) bound to scutellarein (orange VDW) in 1 ns, 50 ns, 100 ns, 150 ns and 200 ns.

As the results of text-mining demonstrated, scutellarein was proved to exert an inhibitory effect *in vitro*. The complex was also shown to be stable over a 50 ns molecular dynamics simulation timeframe. To provide further indications of its stability and to determine whether ligand binding induces substantial conformational change, a significantly longer molecular dynamics simulation, with a duration of 200 ns, was conducted to examine the sustained stability of scutellarein binding to the XDH active site. **Figures 9A–F** depict the results from time-series analysis comparing the *apo* XDH protein and the scutellarein-bound XDH protein. Consistent with the time-series trends from section 3.7, scutellarein maintained a similar magnitude of RMSD, Rg, and SASA after equilibration at approximately 20 ns, with a mean of 0.213 nm, 3.21 nm, and 489.2 Å^2^, respectively. These results do not differ greatly from that of the *apo*-XDH protein: 0.218 nm for RMSD, 3.22 nm for Rg, and 489.5 Å^2^ for SASA. Interestingly, as observed in the RMSD, Rg, and SASA values in **Figures 9A–C**, scutellarein binding achieved greater compactness and stability of protein folding compared to the absence of ligand in this longer simulation.

Additionally, the possibility of further compactness following a longer simulation was reflected in the increasing frequency of hydrogen bonds. Initially, a mean of 1-2 bonds stabilized the binding of scutellarein. After approximately 75 ns, there was an increase to 3-4 bonds, as depicted through the highly dense thick bands in **Figure 9E**. Although the number of pairs within 0.35 nm in **Figure 9F** showed a slight decline in numbers shortly after system relaxation at 20 ns, the fluctuation in magnitude and degree remained consistent, following a sine wave trend.

It is observed in **Figure 9D** that there was no significant motion in the structure of scutellarein with a mean RMSD of 0.041 nm. This low value implies effective and stable binding at the XDH active site. Snapshots of scutellarein (orange VDW) bound to XDH (blue cartoons) from the initial through to the final stages of the simulation showed that the ligand was tightly bound to the XDH active pocket, as illustrated in **Figure 9H**. A closer examination of the ligand structure demonstrated that it shifted from its initial starting position and settled into a stable conformation soon after equilibration, in which it remained throughout the remainder of the 200 ns simulation (**Supplementary Figure S6** and **Supplementary Video S1**).

Per-residue RMSF plots in **Figure 9G** also support the time-series analyses and suggest greater stability and less fluctuation of the XDH active site residues upon binding to scutellarein. Generally, the RMSF of scutellarein-bound XDH residues (orange) was lower than the *apo*-protein (dark blue). In particular, the residues between Gln1041 and Asn1074, as highlighted in blue in an in-set, appeared to become more rigid with ligand binding, showing a lower RMSF. Two exceptions were residues Pro1110 and Arg1246; however, they both were situated a distance away from the scutellarein binding site.

## 4 Discussion

HUA is a metabolic disorder syndrome caused by overproduction or underexcretion of serum urate. Many dietary factors were proven to contribute to the excessive production of serum urate and increased HUA prevalence, such as seafood intake (7, 69, 70). PF, the leaves of the leaf of *Perilla frutescens* (L.) Britt, is a very common plant-based functional food, which has a long history as herbal therapy used for mitigating the toxicity of fish and crab since the Eastern Han Dynasty (25–220 AD) (71). Hence, we speculate that some of the compounds from PF may play a role in lowering uric acid and preventing HUA underlying such associations. However, whether PF can lower uric acid and the mechanism behind it were rarely reported in previous studies. Here, the identification of the phytochemical composition based on virtual screening, untargeted metabolomics, bioinformatics analysis, and molecular docking was conducted to uncover the possible mechanism of PF treating HUA. Subsequently, structure cluster analysis, visualization of binding modes, ADMET evaluation, and MD simulation analysis were also performed to identify and validate the potential inhibitors against HUA. Our findings provided the basis for the lead identification and development of new anti-HUA agents.

In the bioinformatics analysis, 25 potential targets of PF against HUA were selected and then used for the establishment of the PPI network (**Figure 2B**). The results of the PPI analysis revealed the close interactions and strong biological connectivity among the targets. To uncover the underlying biological mechanism of PF treating HUA, GO and KEGG enrichment analysis and molecular docking were conducted. In the CC terms (**Figure 2D**), the extracellular region part had the lowest *p*-value and contained a total of 15 targets, including ABCC1, SLC22A6, CXCL8, MMP3, TNF, VEGFA, SLC22A12, IL6, PNP, ALDH2, ALB, REN, SLC22A8, XDH, and SLC22A11, with the average binding energy ranging from -6.76 to -4.6 kcal/mol. Among them, XDH possessed the most favorable binding scores, which can be interpreted as PF compounds acting strongly on XDH. In the MF categories of GO analysis (**Supplementary Figure S1**), the term “organic acid transmembrane transporter activity”, which is defined as the activities that enable the transmembrane movement of organic anions, has the most significant *p*-value, where SLC22A12, ABCC1, SLC22A6, ABCC2, SLC22A8, and SLC22A11 were enriched. The average binding affinity of each of these six proteins to PF compounds were -6.16, -6.53, -6.29, -6.19, -6.44, and -6.09 kcal/mol, separately, which can be seen as relatively strong interaction. In the BP terms (**Supplementary Figure S1**), 17 of the 25 proteins (more than 60%) were enriched in “response to oxygen-containing compound”, the most prominent term, with the average binding energy ranging from -4.6 to -6.56 kcal/mol. This can be interpreted as PF being associated with this biological process, which may have relevance to its ability for treating HUA.

In the molecular docking results, from consideration of the compounds (**Supplementary Table S2**), 139 from PF (more than 40%), were predicted to dock well with the 25 potential targets. Interestingly, 16 pan-acting compounds worked on all the 25 proteins and further experimental validation concerning these highly promiscuous compounds is needed. In summary, the above results demonstrated the potential anti-hyperuricemia effect of PF. Consideration of the protein targets (**Figure 3**) and the binding energy histograms for each target class indicated that a high number of ligands bound well with targets in Group (I) while ZH01, namely XDH, was predicted to possess the best average binding results and docked with the most ligands with excellent binding scores (lower than -9 kcal/mol). In addition, a total of 236 compounds (nearly 72%) had good binding energy (< -6 kcal/mol) with XDH while 27 of them were lower than -8.7 kcal/mol, the binding energy of febuxostat. XDH, one of the two forms of xanthine oxidoreductase (XO), enabling the formation of uric acid, is an essential target in the development of urate-lowering agents (24). Febuxostat, as a potent inhibitor against XDH (50), which has been widely used in clinical practice, has a good curative effect on patients with HUA or gout (21, 72). The molecular docking results indicated that many compounds of PF bound well with XDH while some of them were bound even more tightly than febuxostat. It is reasonable for us to speculate that some of the PF compounds may have the potential to be applied for urate-lowering therapy in the future.

As the molecular structure generally determines the binding affinity and biological function, we performed a cluster analysis according to the molecular structural similarity and molecular docking results with the assistance of DataWarrior. On the one hand, many compounds in PF were different from allopurinol and febuxostat (**Figure 5A** and **Supplementary Figure S2**) which may be driven by the difference between natural ingredients and synthetic drugs. The result that ZS025 possessed the greatest similarity with the standard inhibitors among the 328 compounds may help to partially explain its eminent performance in our study, such as the molecular docking results and the MD simulations with 50 or 200 ns length. On the other hand, the cluster analysis showed that some compounds in PF were clustered by structural similarity and these compounds were mainly identified as prenol lipids, saturated hydrocarbons, fatty acyls, cinnamic acids and derivatives, and phenols (**Figure 5B**). Interestingly, the largest cluster, consisting of the highest number of compounds which were commonly characterized by good binding affinity with XDH, was mainly classified as prenol lipids and sesquiterpenoids. Given that some compounds with specific structures showed impressive XO inhibitory effect (73), prenol lipids and sesquiterpenoids, found in our study, may contribute to the considerable biological interactions between PF compounds and XDH. Such compounds may have the potential to be used for the development of XDH inhibitors.

To further screen for the most promising candidate ligands from PF, we first identified 37 compounds that bound with the active site residues of XDH, as the docking results indicated (**Supplementary Table S6**). Then for these compounds, we performed a PAINS test to remove the false positive compounds and conducted an ADMET evaluation to seek satisfactory ligands based on our pre-defined screening criteria. Finally, five ligands satisfying docking, ADMET, and PAINS criteria were found (**Table 2**). Specifically, all molecules received 0 alerts in the PAINS test and the average binding scores ranged from -7.85 to -6.30 kcal/mol while ZS025 (scutellarein) had the lowest binding energy. In terms of H-bonds, except for ZS090, four compounds formed more than one H-bond. Additionally, ZS093 had the least probability of hepatotoxicity (0.008) (**Supplementary Table S6**).

For the five ligands, we employed MD simulations as a feasibility test to screen for candidate PF compounds as potential XDH inhibitors in HUA (**Figure 7**). Findings from MD simulations between the ligand-free and ligand-bound complexes revealed comparable trends in conformational stability, protein compactness, and fluctuation behaviors. Findings from the time-dependent RMSD analysis suggest that all five PF ligand-protein systems achieved equilibration, although the ligands introduced greater displacements to the overall structure compared to the ligand-free protein. Compared to existing studies on XDH, the values and magnitude of the protein RMSD caused by the PF compounds (between 0.204 to 0.247 nm) were consistent and occasionally outperformed various natural compounds in terms of lower RMSD: quercetin (0.25 nm) and luteolin (0.27 nm) (74), and a potent XDH inhibitor: allopurinol (0.43 nm) (75). This difference may be a result of differing simulation times, as longer simulations may capture greater variations in the protein structure. Despite the relatively stable RMSD values, the compactness and rigidity of the structures measured by the average Rg and SASA differed from another study (75) by approximately 0.32 nm and 169 nm^2^, respectively. Minor differences in protein structures were observed, as the enzyme presents in two interconvertible forms: XDH and XO. It is not surprising that the compactness of XO bound to allopurinol was higher than that of XDH, as shown by higher Rg and SASA values in our study. RMSF values were also comparable to existing studies on MD simulations of similar enzymes. The *apo*-protein showed greater RMSF fluctuation compared to ligand-bound structures around peaks of Gly801 and Asn1074 in our study, consistently echoing the findings from the ligand-bound and ligand-free studies on XO (75). Greater RMSF fluctuations were also observed in the active-site residues of XDH when binding to other naturally occurring compounds (76), with a difference of ~0.1 nm compared to our study. From the hydrogen bond analysis, it is evident that ZS025, followed by ZS252 and ZS056 formed the highest number of polar contacts with XDH residues. Although the polar interactions were fluctuant across the 50 ns time scale, the largely consistent pattern suggests stable and strong interactions at the binding pocket, compared to other ligands such as ZS093. Structural differences leading to higher hydrophilicity of ZS025, ZS252, and ZS056 (xlogP: 1.4, 1.6, and -0.7, respectively) may have contributed to the presence of increased polar contacts. Further mutagenesis studies, focusing on the residues in contact with ligands identified by our docking and simulations, are required to ascertain the roles of these polar contacts on XDH inhibition. Overall, the high binding stability, compactness, and the number of polar contacts between ZS025, ZS252, and ZS056 and XDH residues suggest promising feasibility that these compounds could be candidates for development into future XDH inhibitors.

Next, we conducted literature mining to seek laboratory evidence involved in the five compounds treating HUA (**Supplementary Table S9**). As a result, only scutellarein, a flavonoid, was found to show a potent inhibitory effect on xanthine oxidase activity *in vitro* (77). In addition, we performed a quality control study for PF by using untargeted metabolomics with UPLC-ESI-Q-Orbitrap-MS (**Supplementary Table S10**) and the results showed that scutellarein was one of the 543 identified compounds while the rest of the five potential inhibitors were not found in the PF granule mixture (**Figure 8B**, **Supplementary Table S10**). Consequently, scutellarein became our primary focus, and we conducted a 200 ns MD analysis to further validate its stability when bound to XDH.

Interestingly, MD results with a longer time of 200 ns further demonstrated that scutellarein was able to bind to XDH stably and influence the compactness of the XDH protein. Time-series analysis showed that scutellarein induced a tightening of the complex, contributed by an increased number of polar contacts. Scutellarein was also found to induce rigidity around residues Gln1041 to Asn1074, a major helix and its connected loop motif proximal to the active binding site where a potent inhibitor alloxanthine was found to bind (78). While scutellarein demonstrated the ability to stabilize the residues at the active site ligand binding domain, its interaction with other cofactors such as the FAD prosthetic groups, the iron-sulfur clusters, and the molybdenum atom are worth further investigation in future studies.

Scutellarein, a common ingredient identified in many herbal medicines, such as Scutellaria baicalensis (79) and Perilla frutescens (29, 80), was reported to exert anti-microbial, anti-viral, anti-tumor, hepatoprotective, anti-neurodegenerative, and anti-oxidant effect (81). As the previous studies showed, scutellarein from different sources, including Indian medicinal plants, Perilla frutescens, Salvia plebeia, and Salvia officinalis L., had a potent inhibitory effect on XDH *in vitro* (29, 73, 77, 82) and played a significant role in reducing the serum uric acid *in vivo* (77). Similarly, our study showed that scutellarein bound well with XDH with high binding energy (-7.85 kcal/mol) and two H-bonds as well as forming a stable complex whether during 50 ns or 200 ns MD simulations (**Figures 7**, **9**), which further provides evidence at a molecular level for the underlying mechanism of the abovementioned laboratory studies. Interestingly, in the structure-based analysis, scutellarein showed the greatest similarity with febuxostat among the five inhibitors in the active sites of XDH, and even amongst all 328 PF compounds (**Supplementary Figure S4**, **Figure 5A**). In addition, it generated 0 alerts in the PAINS test, a good lead-like tendency as Lipinski’s rule of five indicated, and no hepatotoxicity. Satisfaction of all of these criteria fully demonstrated that scutellarein has the potential to be a promising therapeutic agent and is worth further investigation (**Table 2**). Additionally, given the broad and mature application of PF in the food industry, on this basis, we plan to develop an anti-hyperuricemic product based on PF, such as oil and condiments, which can be commonly used for many cooking procedures, especially in Asia. Most importantly, we assume that scutellarein can be used as a quality control standard in these potential urate-lowering products. Nevertheless, there are also some limitations in our study. Firstly, the compounds we obtained mainly relied on TCMSP, which was obtained based on literature mining conducted by the developers of the platform (31). Therefore, some newly discovered molecules in recent years may yet have been incorporated into this database, which may result in inconsistent results compared with the data of untargeted metabolomics we performed. Subsequently, despite the molecular docking we conducted showing good binding energy between some PF compounds, the bonding strength between ligands and the macromolecule still needs to be validated using more experiments, such as the surface plasmon resonance technique. Finally, our study was conducted mainly through *in silico* technologies and text-mining, which cannot fully illuminate the *in vivo* biological complexities of PF and its key components. Therefore, our findings need to be further validated in animal models to comprehensively evaluate the actual effect that scutellarein has on serum urate *in vivo*.

## 5 Conclusions

In our study, we first conducted network pharmacology analysis, via various online databases and untargeted metabolomics technology, to explore the potential mechanism of PF treating HUA. Based on enrichment analysis, it is suggested that PF compounds treating HUA may prominently associate with organic acid transmembrane transporter activity (MF), response to oxygen-containing compound (BP), and the extracellular region part (CC). Subsequently, supercomputer-aided molecular docking calculations were implemented to analyze the binding strength between the 326 compounds and the 25 targets. Among them, many compounds were reported to have high binding energy with the potential targets and XDH possessed the top average binding scores with all the 328 compounds. Subsequently, a PAINS test, ADMET properties prediction, and H-bonds analysis were performed to find the most desirable inhibitors against XDH according to our inclusion criteria, and five ligands were eventually identified. Next, MD simulations were conducted to evaluate the stability of the compound-target complexes and they revealed that ZS025, ZS252, and ZS056 had good performance using five basic MD measures of structural stability and dynamics. Finally, we verified the presence of the five compounds in the PF mixture using untargeted metabolomics technology, and scutellarein (ZS025) was found. In addition, we carried out a text-mining study to look for experimental evidence that the compounds may exert biological effects on HUA, and three studies were found to report the pharmacological effect of scutellarein on uric acid. Moreover, MD analysis with a longer time (200 ns) was used to further provide a basis for the stability of the scutellarein-XDH complex. In conclusion, our findings provide fundamental evidence for the potential role of PF, a commonly used plant-based functional food, in the management of HUA, including the development of anti-hyperuricemic agents and products. More *in vitro* and *in vivo* experimental verification will be performed to further investigate the biological effects and potential mechanisms of promising inhibitors we identified in the current study.

## Data availability statement

The original contributions presented in the study are included in the article/**Supplementary material**, further inquiries can be directed to the corresponding author/s.

## Author contributions

CW: Data curation, Formal analysis, Investigation, Software, Visualization, Writing – original draft, Writing – review & editing. AW: Data curation, Formal analysis, Investigation, Software, Validation, Visualization, Writing – original draft, Writing – review & editing. QC: Formal analysis, Investigation, Validation, Visualization, Writing – original draft, Writing – review & editing. SY: Data curation, Visualization, Writing – original draft, Writing – review & editing. MC: Data curation, Formal analysis, Software, Validation, Visualization, Writing – original draft, Writing – review & editing. XS: Methodology, Supervision, Validation, Writing – review & editing. LZ: Funding acquisition, Methodology, Supervision, Validation, Writing – review & editing. YL: Methodology, Supervision, Validation, Writing – review & editing. AY: Methodology, Supervision, Validation, Writing – review & editing. JB: Funding acquisition, Methodology, Supervision, Validation, Writing – review & editing. AH: Conceptualization, Data curation, Formal analysis, Investigation, Methodology, Project administration, Resources, Software, Supervision, Validation, Writing – original draft, Writing – review & editing. HL: Conceptualization, Data curation, Formal analysis, Funding acquisition, Investigation, Methodology, Project administration, Resources, Software, Supervision, Validation, Visualization, Writing – original draft, Writing – review & editing. XZ: Conceptualization, Data curation, Formal analysis, Funding acquisition, Methodology, Project administration, Resources, Supervision, Validation, Writing – review & editing.
