# Supplementary material for: Identification of inhibitors from a functional food-based plant Perillae Folium against hyperuricemia via metabolomics profiling, network pharmacology and all-atom molecular dynamics simulations
Source: Front Endocrinol (Lausanne). 2024 Feb 16;15:1320092. doi: 10.3389/fendo.2024.1320092 (PMC10905266; doi:10.3389/fendo.2024.1320092)
Supplement: Supplementary file 1 [file DataSheet_1.docx]

Supplementary Materials for

Identification of inhibitors from a functional food-based plant Perillae Folium against hyperuricemia via metabolomics profiling and supercomputer-aided drug design

*Chuanghai Wu ^a,b,1^, Ann Rann Wong ^c,1^, Qinghong Chen ^a,b,1^, Xiaomei Chen ^a,b^, Shuxuan Yang ^a,b^, Meilin Chen ^a,b^, Xiaomin Sun ^a^, Yanyan Liu ^a^, Angela Wei Hong Yang ^c^, Jianlu Bi ^d^, Andrew Hung ^e,*^, Hong Li ^a,b,^**^e,*^, Xiaoshan Zhao ^a,b,*^*

Correspondence to: Dr. Andrew Hung andrew.hung@rmit.edu.au

Dr Hong Li gdsyiceman@smu.edu.cn

Prof Xiaoshan Zhao zhaoxs0609@163.com

**Content**

[Fig. S1. Gene ontology enrichment of the candidate targets of Perillae Folium treating hyperuricemia, including the top 10 annotated biological processes and molecular functions terms. 2](#_Toc27800)

[Fig. S2. The visualization of similarity scores of 328 compounds from Perillae Folium compared with allopurinol. 3](#_Toc12970)

[Fig. S3. Details of compounds for clusters 1-7. 4](#_Toc25940)

[Fig. S4. The structural similarity analysis between febuxostat and the five potential inhibitors. 5](#_Toc2663)

[Fig. S5. The mass spectrum of the Perillae Folium granule mixture in negative ion mode. 6](#_Toc16617)

[Fig. S6. Physical behavior of scutellarein in the pocket of xanthine dehydrogenase in 200 ns molecule dynamics simulation. 7](#_Toc21864)

[Table S1. Details of 328 Perillae Folium compounds obtained from the traditional Chinese medicine systems pharmacology database and analysis platform (TCMSP). 8](#_Toc13382)

[Table S3. The interactions between the top 10 active Perillae Folium compounds for each target and all of the 25 candidate targets. 40](#_Toc13638)

[Table S4. Similarity scores of the compounds compared with febuxostat. 41](#_Toc16746)

[Table S5. Classifications of compounds from seven different clusters. 42](#_Toc30657)

[Table S6. Details of 37 compounds identified in the active sites of xanthine dehydrogenase. 46](#_Toc27620)

[Table S7. The search strategy for text-mining. 52](#_Toc5046)

[Table S8. List of the excluded articles with reasons. 53](#_Toc29310)

[Table S9. Characteristics of the included papers for the literature-mining section. 54](#_Toc27298)

[Table S10. The mass spectrometry results of Perillae Folium granules mixture. 55](#_Toc16177)

[Video S1. Movie of the molecular dynamics’ trajectory. 79](#_Toc23358)

[References 80](#_Toc2008)

# Fig. S1. Gene ontology enrichment of the candidate targets of Perillae Folium treating hyperuricemia, including the top 10 annotated biological processes and molecular functions terms.


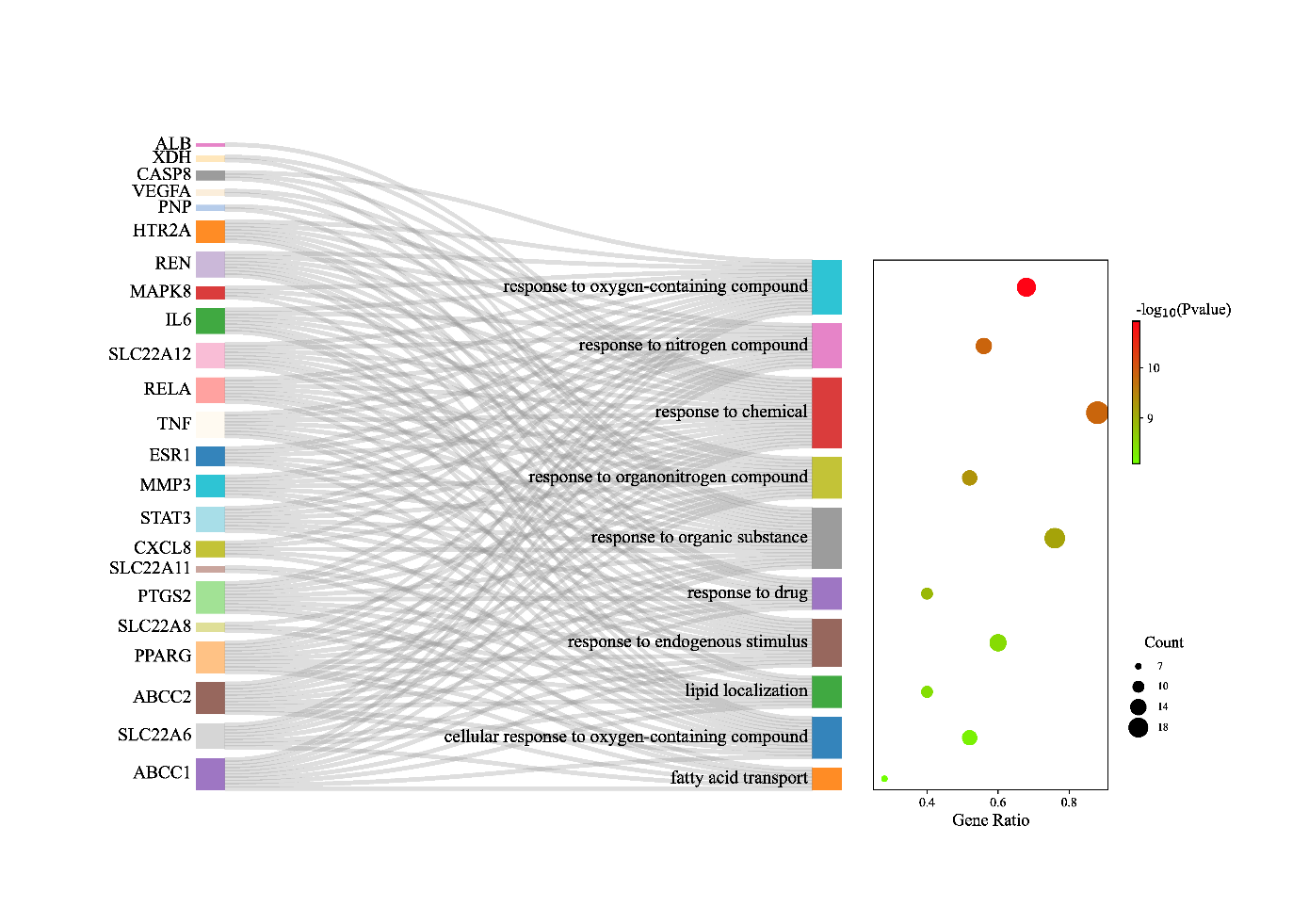

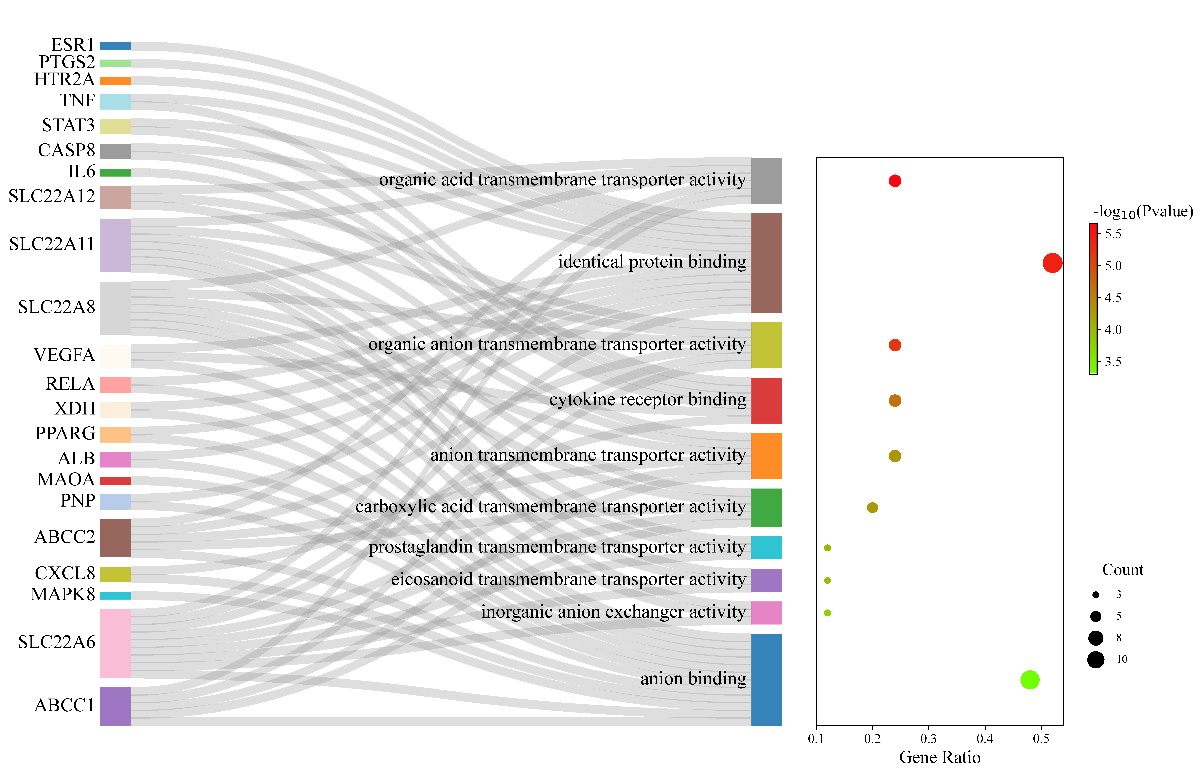


Note: The Sankey diagram is composed of gene names, GO terms, and a bubble plot. The diagram on the top illustrates the result of the BP enrichment analysis, while the bottom indicates the results of the MF analysis. ABCC1, ATP-binding cassette sub-family C member 1; ABCC2, ATP-binding cassette sub-family C member 2; ALB, albumin; ALDH2, aldehyde dehydrogenase, mitochondrial; BP, biological processes; CASP8, caspase-8; CXCL8, chemokine (C-X-C motif) ligand 8; ESR1, estrogen; GO, gene ontology;HTR2A, 5-hydroxytryptamine receptor 2A; IL6, interleukin-6; MAOA, monoamine oxidase type A; MAPK8, mitogen-activated protein kinase 8; MF, molecular functions; MMP3, matrix metalloproteinase-3; PNP, purine nucleoside phosphorylase; PPARG, peroxisome proliferator-activated receptor gamma; PTGS2, prostaglandin G/H synthase 2; RELA, transcription factor p65; REN, renin; SLC22A11, solute carrier family 22 member 11; SLC22A12, solute carrier family 22 member 12; SLC22A6, solute carrier family 22 member 6; SLC22A8, solute carrier family 22 member 8; STAT3, signal transducer and activator of transcription 3; TNF, tumor necrosis factor; VEGFA, vascular endothelial growth factor A; XDH, xanthine dehydrogenase.

# Fig. S2. The visualization of similarity scores of 328 compounds from Perillae Folium compared with allopurinol.


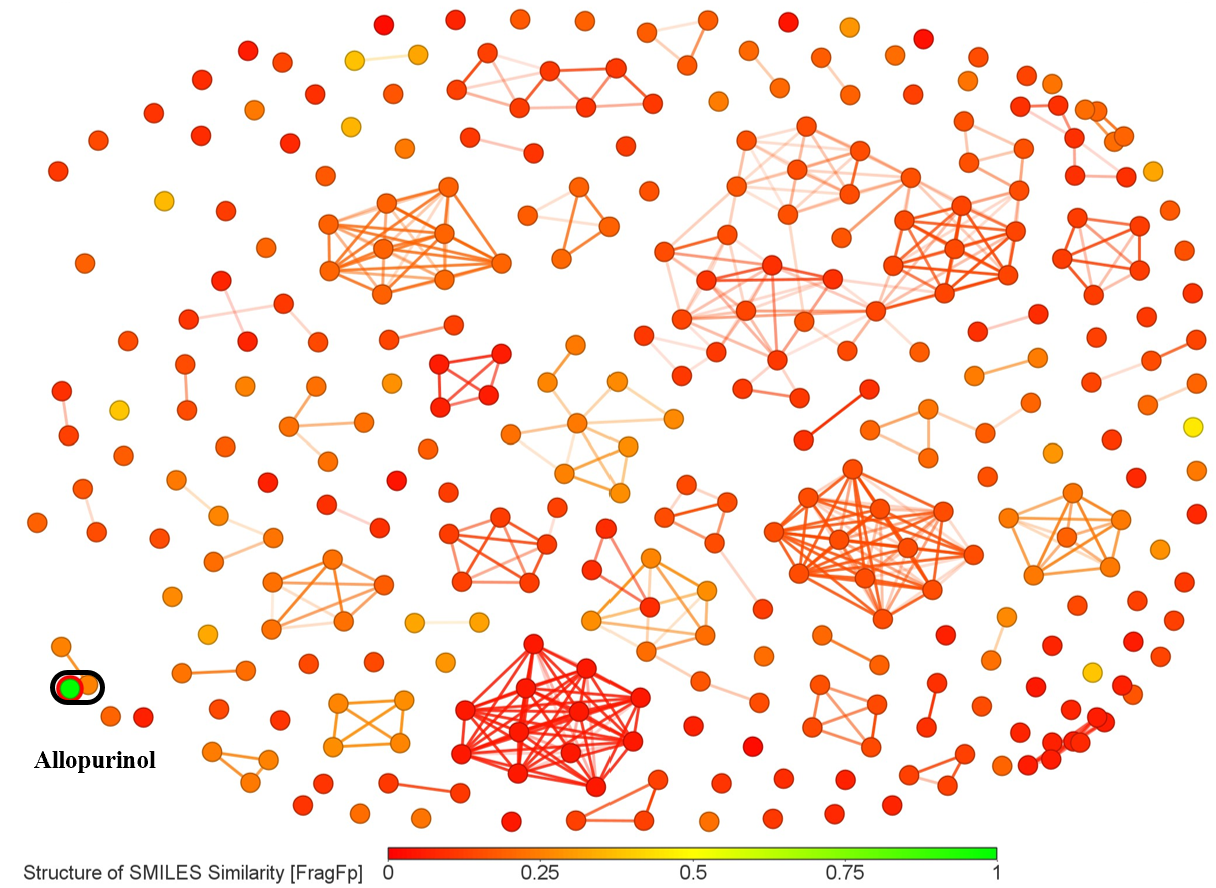


Note: Green nodes represent allopurinol, while other nodes stand for 328 Perillae Folium compounds.

# Fig. S3. Details of compounds for clusters 1-7.


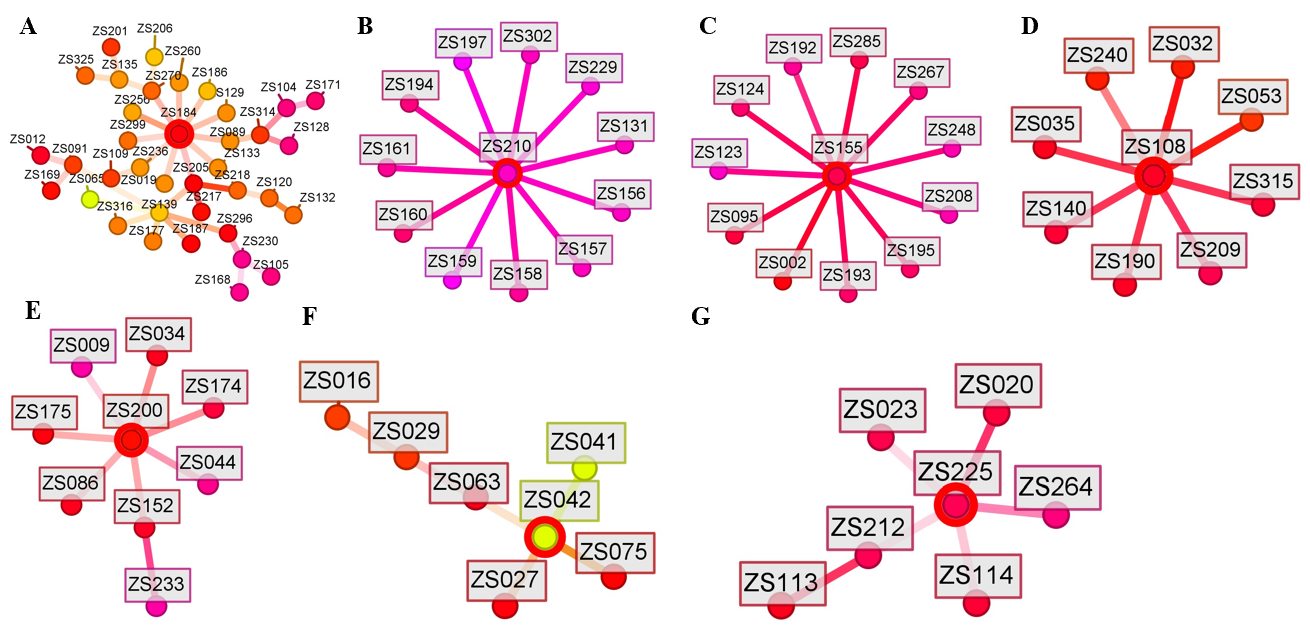


Note: Figure A-G represents clusters 1-7, respectively. Each cluster contains more than or equal to seven compounds.

# Fig. S4. The structural similarity analysis between febuxostat and the five potential inhibitors.


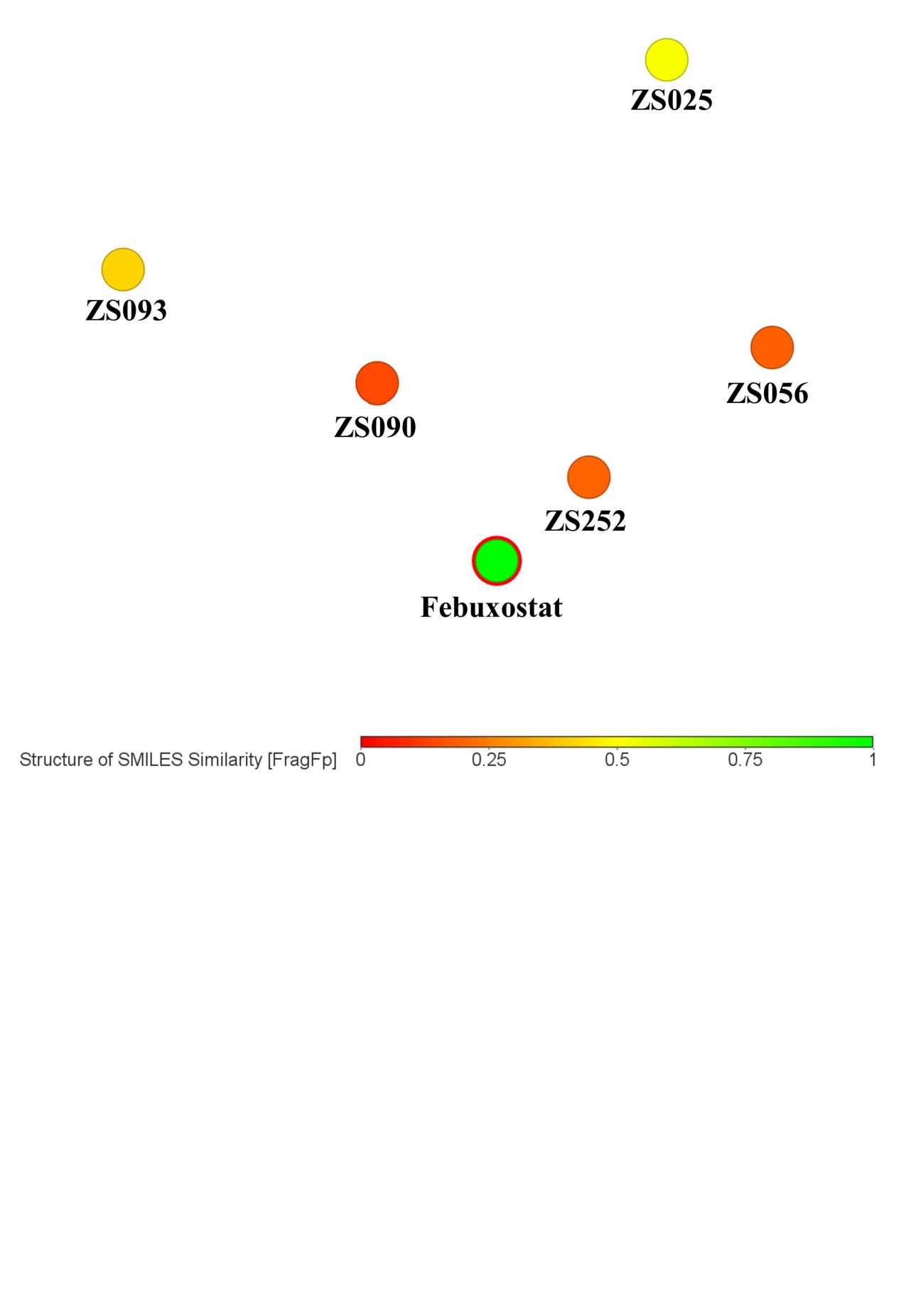


Note: ZS025, scutellarein; ZS056, benzyl alpha-D-mannopyranoside; ZS090, elemol; ZS093, diisobutyl phthalate; ZS252, (3R)-Hydroxy-beta-ionone.

# Fig. S5. The mass spectrum of the Perillae Folium granule mixture in negative ion mode.


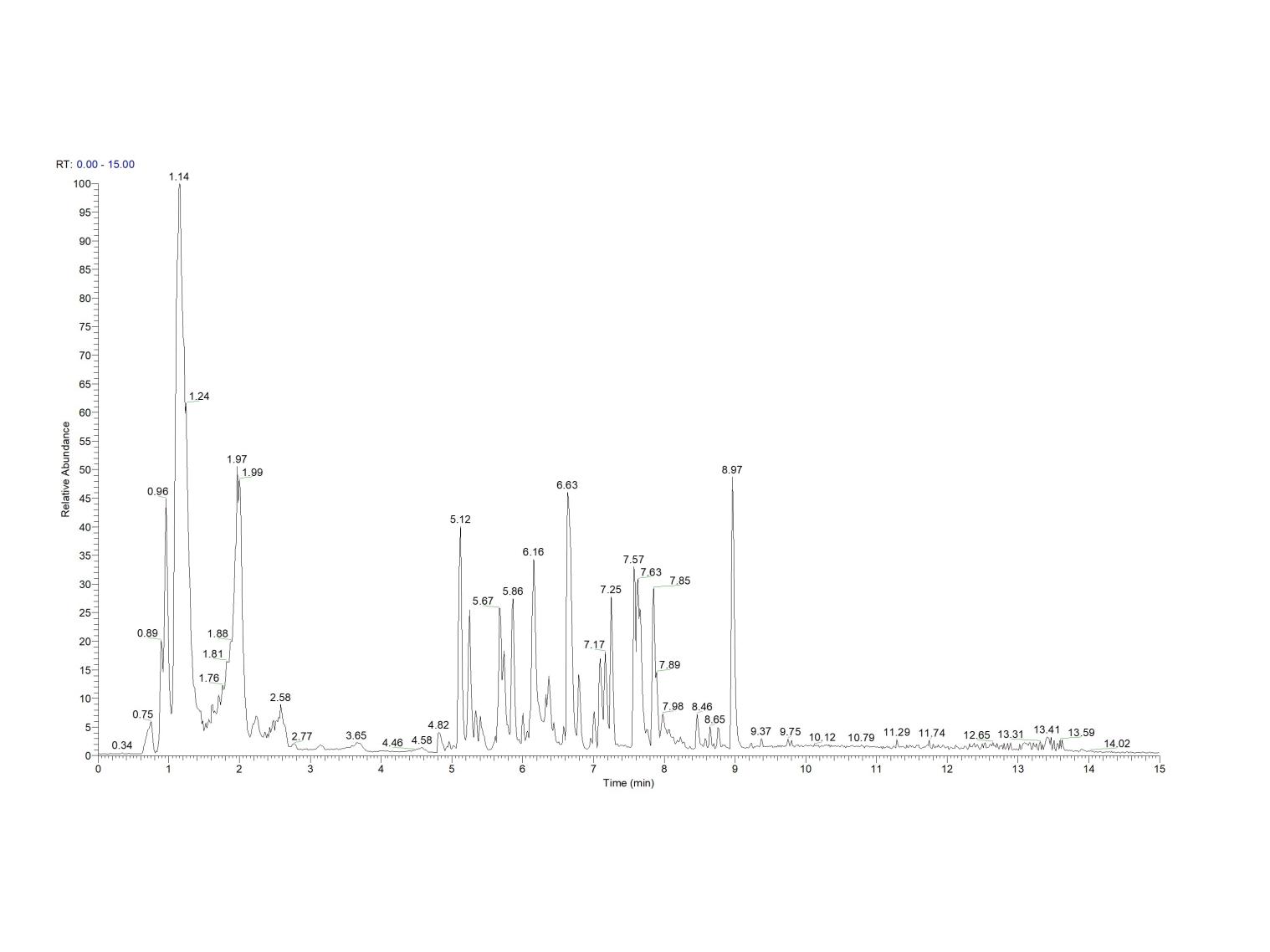


Note: The *x*-axis stands for retention time (min), which ranges from 0 to 15 minutes. The *y*-axis represents the signal intensity. Each peak indicates a unique compound detected in the Perillae Folium granule mixture. The black arrow indicates the inhibitor we focus on, whose schematic diagram is on the top right.

# Fig. S6. Physical behavior of scutellarein in the pocket of xanthine dehydrogenase in 200 ns molecule dynamics simulation.


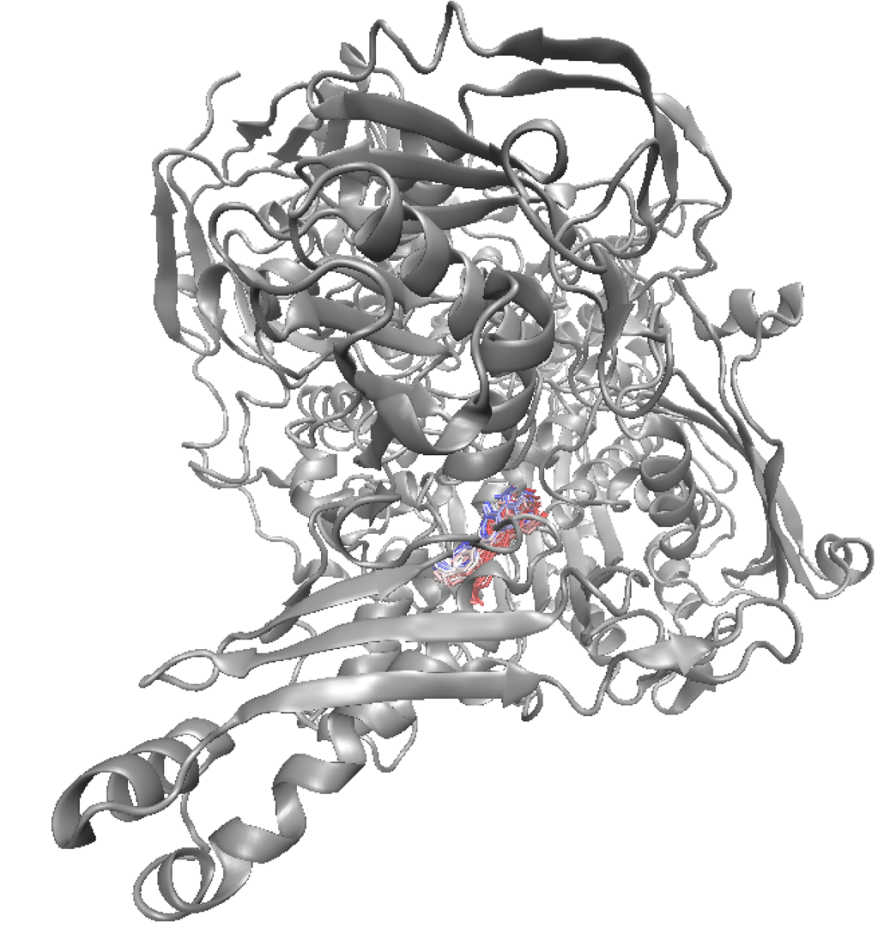


Note: The silver object represents the target (xanthine dehydrogenase) while the red/blue one stands for ligand (scutellarein). The location of the red molecules indicates the initial position while the blue one symbolizes the final.

# Table S1. Details of 328 Perillae Folium compounds obtained from the traditional Chinese medicine systems pharmacology database and analysis platform (TCMSP).

| **Compound code** | **Molecule name** | **Mol ID** | **PubChem CID/SID** | **PubChem name** | **SMILES** |
| --- | --- | --- | --- | --- | --- |
| ZS001 | (L)-alpha-Terpineol | MOL000118 | [443162](http://pubchem.ncbi.nlm.nih.gov/summary/summary.cgi?cid=443162) | (-)-alpha-Terpineol | CC1=CC[C@H](CC1)C(C)(C)O |
| ZS002 | Arachic acid | MOL000012 | [10467](http://pubchem.ncbi.nlm.nih.gov/summary/summary.cgi?cid=10467) | Arachidic acid | CCCCCCCCCCCCCCCCCCCC(=O)O |
| ZS003 | Neral | MOL000127 | 643779 | Neral | CC(=CCC/C(=C\C=O)/C)C |
| ZS004 | d-mandelonitrile | MOL001321 | [9548674](http://pubchem.ncbi.nlm.nih.gov/summary/summary.cgi?cid=9548674) | (R)-mandelonitrile | C1=CC=C(C=C1)[C@H](C#N)O |
| ZS005 | MNN | MOL001366 | [439767](http://pubchem.ncbi.nlm.nih.gov/summary/summary.cgi?cid=439767) | (S)-Mandelonitrile | C1=CC=C(C=C1)[C@@H](C#N)O |
| ZS006 | protocatechualdehyde | MOL001452 | [8768](http://pubchem.ncbi.nlm.nih.gov/summary/summary.cgi?cid=8768) | 3,4-Dihydroxybenzaldehyde | C1=CC(=C(C=C1C=O)O)O |
| ZS007 | α-cubebol | MOL001599 | N/A | N/A | CC1=C[C@@H]2[C@H](CC1)[C@](C)(O)CCC2(C)C |
| ZS008 | Perillyl alcohol | MOL001694 | [369312](http://pubchem.ncbi.nlm.nih.gov/summary/summary.cgi?cid=369312) | (-)-Perillyl alcohol | CC(=C)[C@H]1CCC(=CC1)CO |
| ZS009 | Myrcene | MOL000197 | [31253](http://pubchem.ncbi.nlm.nih.gov/summary/summary.cgi?cid=31253) | Myrcene | CC(=CCCC(=C)C=C)C |
| ZS010 | (R)-linalool | MOL000198 | [443158](http://pubchem.ncbi.nlm.nih.gov/summary/summary.cgi?cid=443158) | (-)-Linalool | CC(=CCC[C@](C)(C=C)O)C |
| ZS011 | cyanidol | MOL000002 | N/A | N/A | Oc1cc(O)c2cc(O)c(-c3ccc(O)c(O)c3)[o+]c2c1 |
| ZS012 | -cis-.beta.-Elemene diastereomer | MOL000204 | [6431152](http://pubchem.ncbi.nlm.nih.gov/summary/summary.cgi?cid=6431152) | (1S,2R,4R)-1-ethenyl-1-methyl-2 | CC(=C)[C@@H]1CC[C@@]([C@H](C1)C(=C)C)(C)C=C |
| ZS013 | thymol | MOL002042 | [6989](http://pubchem.ncbi.nlm.nih.gov/summary/summary.cgi?cid=6989) | Thymol | CC1=CC(=C(C=C1)C(C)C)O |
| ZS014 | luteolin-7-o-glucoside | MOL000009 | [5280637](http://pubchem.ncbi.nlm.nih.gov/summary/summary.cgi?cid=5280637) | Cynaroside | C1=CC(=C(C=C1C2=CC(=O)C3=C(C=C(C=C3O2)O[C@H]4[C@@H]([C@H]([C@@H]([C@H](O4)CO)O)O)O)O)O)O |
| ZS015 | 1,3,8-p-Menthatriene | MOL002128 | [176983](http://pubchem.ncbi.nlm.nih.gov/summary/summary.cgi?cid=176983) | p-Mentha-1,3,8-triene | CC1=CC=C(CC1)C(=C)C |
| ZS016 | caffeic acid | MOL000223 | [689043](http://pubchem.ncbi.nlm.nih.gov/summary/summary.cgi?cid=689043) | caffeic acid | C1=CC(=C(C=C1/C=C/C(=O)O)O)O |
| ZS017 | beta-Ionone | MOL002363 | [638014](http://pubchem.ncbi.nlm.nih.gov/summary/summary.cgi?cid=638014) | beta-Ionone | CC1=C(C(CCC1)(C)C)/C=C/C(=O)C |
| ZS018 | (Z,Z)-farnesol | MOL000247 | [1549107](http://pubchem.ncbi.nlm.nih.gov/summary/summary.cgi?cid=1549107) | cis,cis-Farnesol | CC(=CCC/C(=C\CC/C(=C\CO)/C)/C)C |
| ZS019 | copaene | MOL002502 | N/A | N/A | CC1=CC[C@@H]2[C@@H]3[C@H](C(C)C)CC[C@@]2(C)[C@@H]13 |
| ZS020 | eugenol | MOL000254 | [3314](http://pubchem.ncbi.nlm.nih.gov/summary/summary.cgi?cid=3314) | Eugenol | C=CCc1ccc(O)c(OC)c1 |
| ZS021 | o-Thymol | MOL000259 | [10364](http://pubchem.ncbi.nlm.nih.gov/summary/summary.cgi?cid=10364) | Carvacrol | CC1=C(C=C(C=C1)C(C)C)O |
| ZS022 | Myristicin | MOL000261 | [4276](http://pubchem.ncbi.nlm.nih.gov/summary/summary.cgi?cid=4276) | Myristicin | COC1=CC(=CC2=C1OCO2)CC=C |
| ZS023 | Elemicin | MOL000269 | [10248](http://pubchem.ncbi.nlm.nih.gov/summary/summary.cgi?cid=10248) | Elemicin | COC1=CC(=CC(=C1OC)OC)CC=C |
| ZS024 | luteolin | MOL000006 | [5280445](http://pubchem.ncbi.nlm.nih.gov/summary/summary.cgi?cid=5280445) | Luteolin | C1=CC(=C(C=C1C2=CC(=O)C3=C(C=C(C=C3O2)O)O)O)O |
| ZS025 | scutellarein | MOL002737 | [5281697](http://pubchem.ncbi.nlm.nih.gov/summary/summary.cgi?cid=5281697) | Scutellarein | C1=CC(=CC=C1C2=CC(=O)C3=C(O2)C=C(C(=C3O)O)O)O |
| ZS026 | scutellarin | MOL002931 | [185617](http://pubchem.ncbi.nlm.nih.gov/summary/summary.cgi?cid=185617) | Scutellarin | C1=CC(=CC=C1C2=CC(=O)C3=C(C(=C(C=C3O2)O[C@H]4[C@@H]([C@H]([C@@H]([C@H](O4)C(=O)O)O)O)O)O)O)O |
| ZS027 | Methyl caffeate | MOL006218 | [689075](http://pubchem.ncbi.nlm.nih.gov/summary/summary.cgi?cid=689075) | Methyl caffeate | COC(=O)/C=C/C1=CC(=C(C=C1)O)O |
| ZS028 | Methyl protocatechuate | MOL003503 | [287064](http://pubchem.ncbi.nlm.nih.gov/summary/summary.cgi?cid=287064) | Methyl 3,4-dihydroxybenzoate | COC(=O)C1=CC(=C(C=C1)O)O |
| ZS029 | FER | MOL000360 | [445858](http://pubchem.ncbi.nlm.nih.gov/summary/summary.cgi?cid=445858) | Ferulic acid | COC1=C(C=CC(=C1)/C=C/C(=O)O)O |
| ZS030 | Methyl geranate | MOL003622 | [5365910](http://pubchem.ncbi.nlm.nih.gov/summary/summary.cgi?cid=5365910) | Methyl geranate | CC(=CCC/C(=C/C(=O)OC)/C)C |
| ZS031 | esculetin | MOL003837 | [5281416](http://pubchem.ncbi.nlm.nih.gov/summary/summary.cgi?cid=5281416) | Esculetin | C1=CC(=O)OC2=CC(=C(C=C21)O)O |
| ZS032 | linolenic acid | MOL000432 | [5280934](http://pubchem.ncbi.nlm.nih.gov/summary/summary.cgi?cid=5280934) | Linolenic acid | CC/C=C\C/C=C\C/C=C\CCCCCCCC(=O)O |
| ZS033 | Cumic acid | MOL004688 | [10820](http://pubchem.ncbi.nlm.nih.gov/summary/summary.cgi?cid=10820) | 4-Isopropylbenzoic acid | CC(C)C1=CC=C(C=C1)C(=O)O |
| ZS034 | Farnesene | MOL000479 | [5281517](http://pubchem.ncbi.nlm.nih.gov/summary/summary.cgi?cid=5281517) | beta-Farnesene | CC(=CCC/C(=C/CCC(=C)C=C)/C)C |
| ZS035 | gondoic acid | MOL005030 | [5282768](http://pubchem.ncbi.nlm.nih.gov/summary/summary.cgi?cid=5282768) | cis-11-Eicosenoic acid | CCCCCCCC/C=C\CCCCCCCCCC(=O)O |
| ZS036 | ursolic acid | MOL000511 | [64945](http://pubchem.ncbi.nlm.nih.gov/summary/summary.cgi?cid=64945) | Ursolic acid | C[C@@H]1CC[C@@]2(CC[C@@]3(C(=CC[C@H]4[C@]3(CC[C@@H]5[C@@]4(CC[C@@H](C5(C)C)O)C)C)[C@@H]2[C@H]1C)C)C(=O)O |
| ZS037 | (-)-Caryophyllene oxide | MOL002003 | N/A | N/A | C=C1CC[C@@H]2O[C@]2(C)CC[C@@H]2[C@@H]1CC2(C)C |
| ZS038 | AMG | MOL005180 | [76935](http://pubchem.ncbi.nlm.nih.gov/summary/summary.cgi?cid=76935) | Methyl alpha-D-galactopyranoside | CO[C@@H]1[C@@H]([C@H]([C@H]([C@H](O1)CO)O)O)O |
| ZS039 | Perilloside A | MOL005691 | [3086657](http://pubchem.ncbi.nlm.nih.gov/summary/summary.cgi?cid=3086657) | Perilloside A | CC(=C)[C@H]1CCC(=CC1)CO[C@H]2[C@@H]([C@H]([C@@H]([C@H](O2)CO)O)O)O |
| ZS040 | ZINC04521547 | MOL006189 | [7269382](http://pubchem.ncbi.nlm.nih.gov/summary/summary.cgi?cid=7269382) | (3R)-hept-1-en-3-ol | CCCC[C@H](C=C)O |
| ZS041 | [(Z)-2-(3,5-dihydroxyphenyl)vinyl] (E)-3-(3,4-dihydroxyphenyl)prop-2-enoate | MOL006190 | 5316820 | Nepetoidin A | C1=CC(=C(C=C1/C=C/C(=O)O/C=C\C2=CC(=CC(=C2)O)O)O)O |
| ZS042 | [(Z)-2-(3,4-dihydroxyphenyl)vinyl] (E)-3-(3,4-dihydroxyphenyl)prop-2-enoate | MOL006191 | [5316819](http://pubchem.ncbi.nlm.nih.gov/summary/summary.cgi?cid=5316819) | Nepetoidin B | C1=CC(=C(C=C1/C=C/C(=O)O/C=C\C2=CC(=C(C=C2)O)O)O)O |
| ZS043 | (R,E)-3-(3-ethoxy-4-hydroxyphenyl)-2-((3-(3-ethoxy-4-hydroxyphenyl)acryloyl)oxy)propanoic acid | MOL006192 | N/A | N/A | CCOc1cc(C=CC(=O)O[C@H](Cc2ccc(O)c(OCC)c2)C(=O)O)ccc1O |
| ZS044 | Citralva | MOL006193 | [1551246](http://pubchem.ncbi.nlm.nih.gov/summary/summary.cgi?cid=1551246) | 3,7-Dimethylocta-2,6-dienenitrile | CC(=CCC/C(=C/C#N)/C)C |
| ZS045 | 3-β-D-glucopyranosyl-3-epi-2-isocucurbic acid | MOL006194 | N/A | N/A | CCC=CC[C@@H]1CC[C@H](O[C@H]2O[C@H](CO)[C@@H](O)[C@H](O)[C@@H]2O)[C@H]1CC(=O)O |
| ZS046 | 3-β-D-glucopyranosyl-3-epi-2-isocucurbic acid_qt | MOL006195 | N/A | N/A | CCC=CC[C@@H]1CC[C@H](O)[C@H]1CC(=O)O |
| ZS047 | 3-β-D-glucopyranosyloxy-5-phenylvaleric acid | MOL006196 | N/A | N/A | O=C(O)C[C@@H](CCc1ccccc1)O[C@H]1O[C@H](CO)[C@@H](O)[C@H](O)[C@@H]1O |
| ZS048 | (3R)-3-hydroxy-5-phenyl-valeric acid | MOL006197 | [9815528](http://pubchem.ncbi.nlm.nih.gov/summary/summary.cgi?cid=9815528) | (r)-3-Hydroxy-5-phenylvaleric acid | C1=CC=C(C=C1)CC[C@H](CC(=O)O)O |
| ZS049 | APIOL | MOL006198 | [10659](http://pubchem.ncbi.nlm.nih.gov/summary/summary.cgi?cid=10659) | Apiole | COC1=C2C(=C(C(=C1)CC=C)OC)OCO2 |
| ZS050 | 4-(2-Butyl)phenol | MOL006199 | [38989049](http://pubchem.ncbi.nlm.nih.gov/summary/summary.cgi?cid=38989049) | 4-[(2~{R})-butan-2-yl]phenol | CC[C@@H](C)C1=CC=C(C=C1)O |
| ZS051 | 5'-β-D-glucopyranosyloxyjasmonic acid | MOL006200 | N/A | N/A | O=C(O)C[C@H]1CCC(=O)[C@H]1CC=CCCO[C@H]1O[C@H](CO)[C@@H](O)[C@H](O)[C@@H]1O |
| ZS052 | Tuberonic acid | MOL006201 | [6443968](http://pubchem.ncbi.nlm.nih.gov/summary/summary.cgi?cid=6443968) | Tuberonic acid | C1CC(=O)[C@H]([C@H]1CC(=O)O)C/C=C\CCO |
| ZS053 | LAX | MOL006202 | [5280581](http://pubchem.ncbi.nlm.nih.gov/summary/summary.cgi?cid=5280581) | Dihomo-gamma-linolenic acid | CCCCC/C=C\C/C=C\C/C=C\CCCCCCC(=O)O |
| ZS054 | SBB009008 | MOL006203 | [76015](http://pubchem.ncbi.nlm.nih.gov/summary/summary.cgi?cid=76015) | 10-Undecyn-1-ol | C#CCCCCCCCCCO |
| ZS055 | antheraxanthin | MOL006204 | N/A | N/A | CC(C=CC=C(C)C=CC1=C(C)C[C@H](O)CC1(C)C)=CC=CC=C(C)C=CC=C(C)C=C[C@]12O[C@@]1(C)C[C@H](O)CC2(C)C |
| ZS056 | Benzyl alpha-D-mannopyranoside | MOL006205 | [10956572](http://pubchem.ncbi.nlm.nih.gov/summary/summary.cgi?cid=10956572) | Benzyl alpha-D-mannopyranoside | C1=CC=C(C=C1)CO[C@@H]2[C@H]([C@H]([C@@H]([C@H](O2)CO)O)O)O |
| ZS057 | beta-carotene | MOL002773 | [5280489](http://pubchem.ncbi.nlm.nih.gov/summary/summary.cgi?cid=5280489) | beta-Carotene | CC1=C(C(CCC1)(C)C)/C=C/C(=C/C=C/C(=C/C=C/C=C(/C=C/C=C(/C=C/C2=C(CCCC2(C)C)C)\C)\C)/C)/C |
| ZS058 | caffeylcyanin | MOL006207 | N/A | N/A | O=C(C=Cc1ccc(O)c(O)c1)OC[C@@H]1O[C@@H](Oc2cc3c(O[C@H]4O[C@@H](CO)[C@H](O)[C@@H](O)[C@H]4O)cc(O)cc3[o+]c2-c2ccc(O)c(O)c2)C[C@H](O)[C@@H]1O |
| ZS059 | caffeylmalonylcyanin | MOL006208 | N/A | N/A | O=C(O)CC(=O)OC[C@@H]1O[C@H](Oc2cc(O)cc3[o+]c(-c4ccc(O)c(O)c4)c(O[C@H]4C[C@H](O)[C@H](O)[C@H](COC(=O)C=Cc5ccc(O)c(O)c5)O4)cc23)[C@H](O)[C@H](O)[C@H]1O |
| ZS060 | cyanin | MOL006209 | N/A | N/A | CC(C)CCN1C=CC(=Cc2cc[n+](CCC(C)C)c3ccccc23)c2ccccc21 |
| ZS061 | eugenyl-β-D-glucopyranoside(cirtrusinc) | MOL006210 | N/A | N/A | C=CCc1cccc(O[C@@H]2O[C@H](CO)[C@@H](O)[C@H](O)[C@H]2O)c1OC |
| ZS062 | 3-Allyl-2-methoxyphenol | MOL006211 | 596373 | Phenol, 2-methoxy(2-propenyl)- | COC1=C(C=CC=C1O)CC=C |
| ZS063 | FERULIC ACID METHYL ESTER | MOL006212 | [10176654](http://pubchem.ncbi.nlm.nih.gov/summary/summary.cgi?cid=10176654) | Methyl cis-ferulate | COC1=C(C=CC(=C1)/C=C\C(=O)OC)O |
| ZS064 | linaly-β-D-glucopyranoside | MOL006213 | [38358979](http://pubchem.ncbi.nlm.nih.gov/summary/summary.cgi?cid=38358979) | (R)-Linaryl beta-D-glucopyranoside | CC(=CCC[C@](C)(C=C)O[C@H]1[C@@H]([C@H]([C@@H]([C@H](O1)CO)O)O)O)C |
| ZS065 | progesterone | MOL006214 | [5994](http://pubchem.ncbi.nlm.nih.gov/summary/summary.cgi?cid=5994) | Progesterone | CC(=O)[C@H]1CC[C@@H]2[C@@]1(CC[C@H]3[C@H]2CCC4=CC(=O)CC[C@]34C)C |
| ZS066 | luteolin-3,7-diglucoside | MOL006215 | N/A | N/A | O=c1c(-c2ccc(O)c(O[C@H]3O[C@H](CO)[C@@H](O)[C@H](O)[C@H]3O)c2)coc2cc(O[C@H]3O[C@H](CO)[C@@H](O)[C@H](O)[C@H]3O)cc(O)c12 |
| ZS067 | Santol | MOL006216 | [5281801](http://pubchem.ncbi.nlm.nih.gov/summary/summary.cgi?cid=5281801) | Orobol | C1=CC(=C(C=C1C2=COC3=CC(=CC(=C3C2=O)O)O)O)O |
| ZS068 | malonylshisonin | MOL006217 | N/A | N/A | C=C(OC[C@@H]1O[C@H](Oc2cc(O)cc3[o+]c(-c4ccc(O)c(O)c4)c(O[C@@H]4O[C@@H](COC(=O)C=Cc5ccc(O)cc5)[C@@H](O)[C@@H](O)[C@@H]4O)cc23)[C@@H](O)[C@H](O)[C@@H]1O)C(=O)O |
| ZS069 | Clorius | MOL006219 | [7150](http://pubchem.ncbi.nlm.nih.gov/summary/summary.cgi?cid=7150) | Methyl benzoate | COC(=O)C1=CC=CC=C1 |
| ZS070 | neoxanthin | MOL006220 | N/A | N/A | CC(C=CC=CC(C)=CC=CC(C)=CC[C@@]12O[C@]1(C)C[C@@H](O)CC2(C)C)=CC=CC(C)=CC=C1C(C)(C)C[C@@H](O)C[C@@]1(C)O |
| ZS071 | prunasin | MOL006221 | 92450246 | (2R)-2-phenyl-2-[(2R,3S,4R,5S,6S)-3,4,5-trihydroxy-6-(hydroxymethyl)oxan-2-yl]oxyacetonitrile | C1=CC=C(C=C1)[C@H](C#N)O[C@H]2[C@H]([C@@H]([C@@H]([C@@H](O2)CO)O)O)O |
| ZS072 | Sambunigrin | MOL006222 | N/A | N/A | N#C[C@@H](O[C@H]1O[C@H](CO)[C@@H](O)[C@H](O)[C@@H]1O)c1ccccc1 |
| ZS073 | rosmarinic acid | MOL011865 | [5281792](http://pubchem.ncbi.nlm.nih.gov/summary/summary.cgi?cid=5281792) | Rosmarinic acid | C1=CC(=C(C=C1C[C@H](C(=O)O)OC(=O)/C=C/C2=CC(=C(C=C2)O)O)O)O |
| ZS074 | Shisonin | MOL006224 | N/A | N/A | O=C(C=Cc1ccc(O)cc1)OC[C@@H]1O[C@@H](Oc2cc3c(O[C@H]4O[C@@H](CO)[C@@H](O)[C@@H](O)[C@@H]4O)cc(O)cc3[o+]c2-c2ccc(O)c(O)c2)[C@@H](O)[C@H](O)[C@@H]1O |
| ZS075 | vinyl (E)-3-(3,4-dihydroxyphenyl)prop-2-enoate | MOL006225 | [11127419](http://pubchem.ncbi.nlm.nih.gov/summary/summary.cgi?cid=11127419) | Vinyl caffeate | C=COC(=O)/C=C/C1=CC(=C(C=C1)O)O |
| ZS076 | XAT | MOL013354 | [448438](http://pubchem.ncbi.nlm.nih.gov/summary/summary.cgi?cid=448438) | Violaxanthin | C/C(=C\C=C\C=C(\C=C\C=C(\C=C\[C@@]12O[C@@]1(C[C@H](CC2(C)C)O)C)/C)/C)/C=C/C=C(/C=C/[C@@]34O[C@@]3(C[C@H](CC4(C)C)O)C)\C |
| ZS077 | Elsholtziaketone | MOL006227 | [521240](http://pubchem.ncbi.nlm.nih.gov/summary/summary.cgi?cid=521240) | 3-Methyl-1-(3-methylfuran-2-yl)butan-1-one | CC1=C(OC=C1)C(=O)CC(C)C |
| ZS078 | [(2S,3R,4S,5S,6R)-3,4,5-trihydroxy-6-(hydroxymethyl)oxan-2-yl] (4S)-4-prop-1-en-2-ylcyclohexene-1-carboxylate | MOL006228 | [21631012](http://pubchem.ncbi.nlm.nih.gov/summary/summary.cgi?cid=21631012) | [(2S,3R,4S,5S,6R)-3,4,5-trihydroxy-6-(hydroxymethyl)oxan-2-yl] (4S)-4-prop-1-en-2-ylcyclohexene-1-carboxylate | CC(=C)[C@H]1CCC(=CC1)C(=O)O[C@H]2[C@@H]([C@H]([C@@H]([C@H](O2)CO)O)O)O |
| ZS079 | perilloside C | MOL006229 | N/A | N/A | C=C(C)[C@H]1CC[C@H](CO[C@@H]2O[C@H](O)[C@@H](O)[C@H](O)[C@H]2O)CC1 |
| ZS080 | perilloside c_qt | MOL006230 | [519954](http://pubchem.ncbi.nlm.nih.gov/summary/summary.cgi?cid=519954) | [4-(Prop-1-en-2-yl)cyclohexyl]methanol | CC(=C)C1CCC(CC1)CO |
| ZS081 | perilloside D | MOL006231 | [21631019](http://pubchem.ncbi.nlm.nih.gov/summary/summary.cgi?cid=21631019) | (2R,3S,4S,5R,6R)-2-(hydroxymethyl)-6-[(4-prop-1-en-2-ylcyclohexyl)methoxy]oxane-3,4,5-triol | CC(=C)C1CCC(CC1)CO[C@H]2[C@@H]([C@H]([C@@H]([C@H](O2)CO)O)O)O |
| ZS082 | Perilla ketone | MOL006232 | [68381](http://pubchem.ncbi.nlm.nih.gov/summary/summary.cgi?cid=68381) | Perilla ketone | CC(C)CCC(=O)C1=COC=C1 |
| ZS083 | Cosmetin | MOL000007 | [5280704](http://pubchem.ncbi.nlm.nih.gov/summary/summary.cgi?cid=5280704) | Cosmosiin | C1=CC(=CC=C1C2=CC(=O)C3=C(C=C(C=C3O2)O[C@H]4[C@@H]([C@H]([C@@H]([C@H](O4)CO)O)O)O)O)O |
| ZS084 | apigenin | MOL000008 | [5280443](http://pubchem.ncbi.nlm.nih.gov/summary/summary.cgi?cid=5280443) | Apigenin | C1=CC(=CC=C1C2=CC(=O)C3=C(C=C(C=C3O2)O)O)O |
| ZS085 | Germacron | MOL000910 | [6436348](http://pubchem.ncbi.nlm.nih.gov/summary/summary.cgi?cid=6436348) | Germacrone | C/C/1=C\CC(=C(C)C)C(=O)C/C(=C/CC1)/C |
| ZS086 | alpha-Farnesene | MOL000932 | [5281516](http://pubchem.ncbi.nlm.nih.gov/summary/summary.cgi?cid=5281516) | alpha-Farnesene | CC(=CCC/C(=C/C/C=C(\C)/C=C)/C)C |
| ZS087 | (3R,6E,10S)-3-isopropyl-6,10-dimethylcyclodec-6-ene-1,4-dione | MOL000957 | [10466651](http://pubchem.ncbi.nlm.nih.gov/summary/summary.cgi?cid=10466651) | CID 10466651 | C[C@H]1CC/C=C(/CC(=O)[C@H](CC1=O)C(C)C)\C |
| ZS088 | Hemo-sol | MOL000023 | [440917](http://pubchem.ncbi.nlm.nih.gov/summary/summary.cgi?cid=440917) | D-Limonene | CC1=CC[C@@H](CC1)C(=C)C |
| ZS089 | α-Longipinene | MOL000025 | 12311396 | (+)-alpha-Longipinene | CC1=CC[C@@H]2[C@@H]3[C@H]1[C@]2(CCCC3(C)C)C |
| ZS090 | 2-[(1R,3S,4S)-3-isopropenyl-4-methyl-4-vinylcyclohexyl]propan-2-ol | MOL000034 | [92138](http://pubchem.ncbi.nlm.nih.gov/summary/summary.cgi?cid=92138) | Elemol | CC(=C)[C@@H]1C[C@@H](CC[C@@]1(C)C=C)C(C)(C)O |
| ZS091 | beta-Selinene | MOL000035 | [442393](http://pubchem.ncbi.nlm.nih.gov/summary/summary.cgi?cid=442393) | beta-Selinene | CC(=C)[C@@H]1CC[C@]2(CCCC(=C)[C@@H]2C1)C |
| ZS092 | γ-elemene | MOL000037 | [12309452](http://pubchem.ncbi.nlm.nih.gov/summary/summary.cgi?cid=12309452) | 1-Ethenyl-1-methyl-2-(1-methylethenyl)-4-(1-methylethylidene)-cyclohexane | CC(=C1CC[C@]([C@H](C1)C(=C)C)(C)C=C)C |
| ZS093 | DIBP | MOL000057 | [6782](http://pubchem.ncbi.nlm.nih.gov/summary/summary.cgi?cid=6782) | Diisobutyl phthalate | CC(C)COC(=O)C1=CC=CC=C1C(=O)OCC(C)C |
| ZS094 | 2-[(2R,5S,6S)-6,10-dimethylspiro[4.5]dec-9-en-2-yl]propan-2-ol | MOL000058 | [10878761](http://pubchem.ncbi.nlm.nih.gov/summary/summary.cgi?cid=10878761) | CID 10878761 | C[C@H]1CCC=C([C@]12CC[C@H](C2)C(C)(C)O)C |
| ZS095 | palmitic acid | MOL000069 | [985](http://pubchem.ncbi.nlm.nih.gov/summary/summary.cgi?cid=985) | Palmitic acid | CCCCCCCCCCCCCCCC(=O)O |
| ZS096 | Aldrich | MOL000077 | [7041](http://pubchem.ncbi.nlm.nih.gov/summary/summary.cgi?cid=7041) | 2,6-Dimethoxyphenol | COC1=C(C(=CC=C1)OC)O |
| ZS097 | Nonanal | MOL000116 | [31289](http://pubchem.ncbi.nlm.nih.gov/summary/summary.cgi?cid=31289) | Nonanal | CCCCCCCCC=O |
| ZS098 | Cymol | MOL000117 | 7463 | P-Cymene | CC1=CC=C(C=C1)C(C)C |
| ZS099 | ZINC02040970 | MOL000119 | 11241545 | (3R,6E)-nerolidol | CC(=CCC/C(=C/CC[C@](C)(C=C)O)/C)C |
| ZS100 | Decanal | MOL000121 | 8175 | Decanal | CCCCCCCCCC=O |
| ZS101 | 1,8-cineole | MOL000122 | 2758 | Eucalyptol | CC1(C2CCC(O1)(CC2)C)C |
| ZS102 | geraniol | MOL000123 | 637566 | Geraniol | CC(=CCC/C(=C/CO)/C)C |
| ZS103 | citral | MOL000124 | 638011 | Citral | CC(=CCC/C(=C/C=O)/C)C |
| ZS104 | (-)-alpha-Pinene | MOL000125 | 440968 | (-)-alpha-Pinene | CC1=CC[C@H]2C[C@@H]1C2(C)C |
| ZS105 | (-)-nopinene | MOL000126 | 440967 | (-)-beta-Pinene | CC1([C@H]2CCC(=C)[C@@H]1C2)C |
| ZS106 | NERYLACETATE | MOL000128 | 1549026 | Geranyl acetate | CC(=CCC/C(=C/COC(=O)C)/C)C |
| ZS107 | CAM | MOL000130 | 159055 | D-Camphor | C[C@@]12CC[C@@H](C1(C)C)CC2=O |
| ZS108 | EIC | MOL000131 | 5280450 | Linoleic acid | CCCCC/C=C\C/C=C\CCCCCCCC(=O)O |
| ZS109 | alpha-Guaiene | MOL000169 | 5317844 | alpha-Guaiene | C[C@H]1CC[C@H](CC2=C1CC[C@@H]2C)C(=C)C |
| ZS110 | Furol | MOL000172 | 7362 | Furfural | C1=COC(=C1)C=O |
| ZS111 | Safrol | MOL000199 | 5144 | Safrole | C=CCC1=CC2=C(C=C1)OCO2 |
| ZS112 | Moslene | MOL000202 | 7461 | gamma-Terpinene | CC1=CCC(=CC1)C(C)C |
| ZS113 | isoeugenol | MOL000206 | 853433 | Isoeugenol | C/C=C/C1=CC(=C(C=C1)O)OC |
| ZS114 | Methyleugenol | MOL000207 | 7127 | Methyleugenol | COC1=C(C=C(C=C1)CC=C)OC |
| ZS115 | L-Limonen | MOL000234 | 439250 | (-)-Limonene | CC1=CC[C@H](CC1)C(=C)C |
| ZS116 | farnesol | MOL000252 | 445070 | Farnesol | CC(=CCC/C(=C/CC/C(=C/CO)/C)/C)C |
| ZS117 | (-)-beta-Phellandrene | MOL000257 | 443161 | (-)-beta-Phellandrene | CC(C)[C@H]1CCC(=C)C=C1 |
| ZS118 | oleanolic acid | MOL000263 | 10494 | Oleanolic acid | C[C@]12CC[C@@H](C([C@@H]1CC[C@@]3([C@@H]2CC=C4[C@]3(CC[C@@]5([C@H]4CC(CC5)(C)C)C(=O)O)C)C)(C)C)O |
| ZS119 | Tereben | MOL000264 | 11463 | Terpinolene | CC1=CCC(=C(C)C)CC1 |
| ZS120 | beta-Cubebene | MOL000266 | 93081 | beta-Cubebene | C[C@@H]1CC[C@H]([C@H]2[C@]13[C@@H]2C(=C)CC3)C(C)C |
| ZS121 | (1S,5S)-1-isopropyl-4-methylenebicyclo[3.1.0]hexane | MOL000268 | 11051711 | (-)-Sabinene | CC(C)[C@@]12CCC(=C)[C@@H]1C2 |
| ZS122 | l-carvone | MOL000271 | 439570 | (-)-Carvone | CC1=CC[C@H](CC1=O)C(=C)C |
| ZS123 | caprylic acid | MOL000303 | 379 | Octanoic acid | CCCCCCCC(=O)O |
| ZS124 | lauric acid | MOL000305 | 3893 | Lauric acid | CCCCCCCCCCCC(=O)O |
| ZS125 | beta-sitosterol | MOL000358 | 222284 | beta-Sitosterol | CC[C@H](CC[C@@H](C)[C@H]1CC[C@@H]2[C@@]1(CC[C@H]3[C@H]2CC=C4[C@@]3(CC[C@@H](C4)O)C)C)C(C)C |
| ZS126 | (-)-Epoxycaryophyllene | MOL000474 | 1742210 | Caryophyllene oxide | C[C@@]12CC[C@@H]3[C@H](CC3(C)C)C(=C)CC[C@H]1O2 |
| ZS127 | anethole | MOL000475 | 637563 | Anethole | C/C=C/C1=CC=C(C=C1)OC |
| ZS128 | TMH | MOL000485 | 82227 | (+)-alpha-Pinene | CC1=CC[C@@H]2C[C@H]1C2(C)C |
| ZS129 | (1S,4aR,8aR)-1-isopropyl-7-methyl-4-methylene-2,3,4a,5,6,8a-hexahydro-1H-naphthalene | MOL000489 | 6432404 | (+)-gamma-Cadinene | CC1=C[C@@H]2[C@@H](CC1)C(=C)CC[C@H]2C(C)C |
| ZS130 | (+)-catechin | MOL000492 | 9064 | Cianidanol | C1[C@@H]([C@H](OC2=CC(=CC(=C21)O)O)C3=CC(=C(C=C3)O)O)O |
| ZS131 | Nonacosane | MOL000514 | 12409 | Nonacosane | CCCCCCCCCCCCCCCCCCCCCCCCCCCCC |
| ZS132 | beta-Bourbonene | MOL000611 | 62566 | (-)-beta-Bourbonene | CC(C)[C@@H]1CC[C@@]2([C@H]1[C@@H]3[C@H]2CCC3=C)C |
| ZS133 | (-)-alpha-cedrene | MOL000612 | 6431015 | (-)-alpha-Cedrene | C[C@@H]1CC[C@@H]2[C@]13CC=C([C@H](C3)C2(C)C)C |
| ZS134 | osthol | MOL000614 | 10228 | Osthole | CC(=CCC1=C(C=CC2=C1OC(=O)C=C2)OC)C |
| ZS135 | delta-amorphene | MOL000615 | 441005 | (+)-delta-Cadinene | CC1=C[C@H]2[C@@H](CCC(=C2CC1)C)C(C)C |
| ZS136 | hexanal | MOL000666 | 6184 | Hexanal | CCCCCC=O |
| ZS137 | 1-hexanol | MOL000667 | 8103 | 1-Hexanol | CCCCCCO |
| ZS138 | PENTYLFURAN | MOL000668 | 19602 | 2-Pentylfuran | CCCCCC1=CC=CO1 |
| ZS139 | 58924_FLUKA | MOL000673 | 11127402 | Isolongifolene | CC1(CCC=C2[C@@]13CC[C@@H](C3)C2(C)C)C |
| ZS140 | oleic acid | MOL000675 | 445639 | Oleic acid | CCCCCCCC/C=C\CCCCCCCC(=O)O |
| ZS141 | DBP | MOL000676 | 3026 | Dibutyl phthalate | CCCCOC(=O)C1=CC=CC=C1C(=O)OCCCC |
| ZS142 | patchouli alcohol | MOL000695 | 10955174 | Patchouli alcohol | C[C@H]1CC[C@@]2([C@@]3([C@H]1C[C@H](C2(C)C)CC3)C)O |
| ZS143 | (R)-(-)-alpha-Phellandrene | MOL000698 | 442482 | (R)-(-)-alpha-Phellandrene | CC1=CC[C@@H](C=C1)C(C)C |
| ZS144 | m-Cymol | MOL000699 | 10812 | M-Cymene | CC1=CC(=CC=C1)C(C)C |
| ZS145 | Nerol | MOL000700 | 643820 | Nerol | CC(=CCC/C(=C\CO)/C)C |
| ZS146 | WLN: VHR | MOL000708 | 240 | Benzaldehyde | C1=CC=C(C=C1)C=O |
| ZS147 | (S)-Matsutake alcohol | MOL000709 | 2724898 | (S)-1-Octen-3-ol | CCCCC[C@@H](C=C)O |
| ZS148 | OYA | MOL000710 | 454 | Octanal | CCCCCCCC=O |
| ZS149 | o-Cymol | MOL000712 | 10703 | O-Cymene | CC1=CC=CC=C1C(C)C |
| ZS150 | Hyacinthin | MOL000714 | 998 | Phenylacetaldehyde | C1=CC=C(C=C1)CC=O |
| ZS151 | trans-2-nonenal | MOL000716 | 5283335 | 2-Nonenal | CCCCCC/C=C/C=O |
| ZS152 | Geranylacetone | MOL000724 | 1549778 | Geranylacetone | CC(=CCC/C(=C/CCC(=O)C)/C)C |
| ZS153 | EEE | MOL000775 | 8857 | Ethyl acetate | CCOC(=O)C |
| ZS154 | OXA | MOL000776 | 702 | Ethanol | CCO |
| ZS155 | stearic acid | MOL000860 | 5281 | Stearic acid | CCCCCCCCCCCCCCCCCC(=O)O |
| ZS156 | MYS | MOL000864 | 12391 | Pentadecane | CCCCCCCCCCCCCCC |
| ZS157 | hexadecane | MOL000865 | 11006 | Hexadecane | CCCCCCCCCCCCCCCC |
| ZS158 | Heptadekan | MOL000867 | 12398 | Heptadecane | CCCCCCCCCCCCCCCCC |
| ZS159 | LFA | MOL000868 | 8222 | Eicosane | CCCCCCCCCCCCCCCCCCCC |
| ZS160 | Henicosane | MOL000869 | 12403 | Heneicosane | CCCCCCCCCCCCCCCCCCCCC |
| ZS161 | HEXATRIACONTANE | MOL000870 | 12412 | Hexatriacontane | CCCCCCCCCCCCCCCCCCCCCCCCCCCCCCCCCCCC |
| ZS162 | CYH | MOL000873 | 7967 | Cyclohexanone | C1CCC(=O)CC1 |
| ZS163 | Cedrol | MOL000875 | 65575 | Cedrol | C[C@@H]1CC[C@@H]2[C@]13CC[C@@]([C@H](C3)C2(C)C)(C)O |
| ZS164 | methyl palmitate | MOL000879 | 8181 | Methyl palmitate | CCCCCCCCCCCCCCCC(=O)OC |
| ZS165 | Azulen | MOL000884 | 9231 | Azulene | C1=CC=C2C=CC=C2C=C1 |
| ZS166 | Zingiberene | MOL000895 | 92776 | Zingiberene | CC1=CC[C@@H](C=C1)[C@@H](C)CCC=C(C)C |
| ZS167 | 13657-68-6 | MOL000896 | 6441391 | Germacr-1(10)-ene-5,8-dione | C[C@H]1CC/C=C(/CC(=O)[C@@H](CC1=O)C(C)C)\C |
| ZS168 | ()-beta-Pinene | MOL000905 | 10290825 | (+)-beta-Pinene | CC1([C@@H]2CCC(=C)[C@H]1C2)C |
| ZS169 | beta-elemene | MOL000908 | 6918391 | beta-Elemene | CC(=C)[C@@H]1CC[C@@]([C@@H](C1)C(=C)C)(C)C=C |
| ZS170 | Terpilene | MOL000911 | 7462 | alpha-Terpinene | CC1=CC=C(CC1)C(C)C |
| ZS171 | Car-3-ene | MOL000916 | 442461 | (-)-3-Carene | CC1=CC[C@H]2[C@@H](C1)C2(C)C |
| ZS172 | (R)-p-Menth-1-en-4-ol | MOL000922 | 5325830 | (-)-Terpinen-4-ol | CC1=CC[C@](CC1)(C(C)C)O |
| ZS173 | Mnk | MOL000924 | 8163 | 2-Undecanone | CCCCCCCCCC(=O)C |
| ZS174 | cis-beta-farnesene | MOL000926 | 5317319 | cis-beta-Farnesene | CC(=CCC/C(=C\CCC(=C)C=C)/C)C |
| ZS175 | cis-.alpha.-Farnesene | MOL000930 | 5362889 | (Z,E)-alpha-Farnesene | CC(=CCC/C(=C/C/C=C(/C)\C=C)/C)C |
| ZS176 | Germacrene B | MOL000936 | 5281519 | Germacrene B | C/C/1=C\CC/C(=C/CC(=C(C)C)CC1)/C |
| ZS177 | 58870_FLUKA | MOL000937 | 15431199 | (1aR,4R,7R,7bS)-1,1,4,7-Tetramethyl-1a,2,3,4,5,6,7,7b-octahydro-1H-cyclopropa[e]azulene | C[C@@H]1CC[C@@H]2[C@@H](C2(C)C)C3=C1CC[C@H]3C |
| ZS178 | (1R,4S,4aR,8aR)-4-isopropyl-1,6-dimethyl-3,4,4a,7,8,8a-hexahydro-2H-naphthalen-1-ol | MOL000942 | 10398656 | (-)-alpha-Cadinol | CC1=C[C@H]2[C@@H](CC[C@@]([C@@H]2CC1)(C)O)C(C)C |
| ZS179 | CLR | MOL000953 | 5997 | Cholesterol | C[C@H](CCCC(C)C)[C@H]1CC[C@@H]2[C@@]1(CC[C@H]3[C@H]2CC=C4[C@@]3(CC[C@@H](C4)O)C)C |
| ZS180 | (4S)-4-[(1Z)-1,5-dimethylhexa-1,4-dienyl]-1-methylcyclohexene | MOL000980 | 24798702 | (S,Z)-alpha-bisabolene | CC1=CC[C@H](CC1)/C(=C\CC=C(C)C)/C |
| ZS181 | cinnamaldehyde | MOL000991 | 637511 | Cinnamaldehyde | C1=CC=C(C=C1)/C=C/C=O |
| ZS182 | m-xylene | MOL001098 | 7929 | m-Xylene | CC1=CC(=CC=C1)C |
| ZS183 | cis-beta-Ocimene | MOL001110 | 5320250 | (Z)-beta-Ocimene | CC(=CC/C=C(/C)\C=C)C |
| ZS184 | muurolene | MOL001123 | 12306047 | alpha-Muurolene | CC1=C[C@@H]2[C@H](CC1)C(=CC[C@H]2C(C)C)C |
| ZS185 | l-Verbenone | MOL001129 | 92874 | (-)-Verbenone | CC1=CC(=O)[C@H]2C[C@@H]1C2(C)C |
| ZS186 | γ-muurolene | MOL001180 | 12313020 | (1S,4aS,8aR)-1-isopropyl-7-methyl-4-methylene-1,2,3,4,4a,5,6,8a-octahydronaphthalene | CC1=C[C@@H]2[C@H](CC1)C(=C)CC[C@H]2C(C)C |
| ZS187 | Santalene (minor) | MOL001206 | 6431197 | Santalene (minor) | CC(=CCC[C@@]1([C@@H]2CC[C@@H](C2)C1=C)C)C |
| ZS188 | (S)-p-Mentha-1,8-dien-7-al | MOL001254 | 2724159 | (-)-Perillaldehyde | CC(=C)[C@H]1CCC(=CC1)C=O |
| ZS189 | C09704 | MOL001283 | 5281525 | (3S,6E)-Nerolidol | CC(=CCC/C(=C/CC[C@@](C)(C=C)O)/C)C |
| ZS190 | oleic acid | MOL001308 | 637517 | Elaidic acid | CCCCCCCC/C=C/CCCCCCCC(=O)O |
| ZS191 | WLN: Q1R | MOL001335 | 244 | Benzyl alcohol | C1=CC=C(C=C1)CO |
| ZS192 | 9-Oxononanoic acid | MOL001385 | 75704 | 9-Oxononanoic acid | C(CCCC=O)CCCC(=O)O |
| ZS193 | myristic acid | MOL001393 | 11005 | Myristic acid | CCCCCCCCCCCCCC(=O)O |
| ZS194 | Oktadekan | MOL001394 | 11635 | Octadecane | CCCCCCCCCCCCCCCCCC |
| ZS195 | PENTADECYLIC ACID | MOL001396 | 13849 | Pentadecanoic acid | CCCCCCCCCCCCCCC(=O)O |
| ZS196 | Methyllinolenate | MOL001398 | 5319706 | Methyl linolenate | CC/C=C\C/C=C\C/C=C\CCCCCCCC(=O)OC |
| ZS197 | Octacosane | MOL001402 | 12408 | Octacosane | CCCCCCCCCCCCCCCCCCCCCCCCCCCC |
| ZS198 | phytol | MOL001442 | 5280435 | Phytol | C[C@@H](CCC[C@@H](C)CCC/C(=C/CO)/C)CCCC(C)C |
| ZS199 | FITONE | MOL001487 | 1810796 | (6S,10R)-6,10,14-trimethylpentadecan-2-one | C[C@@H](CCC[C@H](C)CCCC(=O)C)CCCC(C)C |
| ZS200 | Supraene | MOL001506 | [638072](http://pubchem.ncbi.nlm.nih.gov/summary/summary.cgi?cid=638072) | Squalene | CC(=CCC/C(=C/CC/C(=C/CC/C=C(/CC/C=C(/CCC=C(C)C)\C)\C)/C)/C)C |
| ZS201 | Isocaryophyllene | MOL001556 | [5281522](http://pubchem.ncbi.nlm.nih.gov/summary/summary.cgi?cid=5281522) | Isocaryophyllene | C/C/1=C/CCC(=C)[C@H]2CC([C@@H]2CC1)(C)C |
| ZS202 | 1,4,7,-Cycloundecatriene, 1,5,9,9-tetramethyl-, Z,Z,Z- | MOL001570 | [5368784](http://pubchem.ncbi.nlm.nih.gov/summary/summary.cgi?cid=5368784) | 1,5,9,9-Tetramethyl-1,4,7-cycloundecatriene | C/C/1=C/C/C=C(\C/C=C\C(CC1)(C)C)/C |
| ZS203 | Hypnon | MOL001578 | [7410](http://pubchem.ncbi.nlm.nih.gov/summary/summary.cgi?cid=7410) | Acetophenone | CC(=O)C1=CC=CC=C1 |
| ZS204 | Piperitenone | MOL001593 | [381152](http://pubchem.ncbi.nlm.nih.gov/summary/summary.cgi?cid=381152) | Piperitenone | CC1=CC(=O)C(=C(C)C)CC1 |
| ZS205 | copaene | MOL001600 | N/A | N/A | CC1=CC[C@@H]2[C@H]3[C@H](C(C)C)CC[C@]2(C)[C@@H]13 |
| ZS206 | 1,4-cadinadiene | MOL001602 | [50986185](http://pubchem.ncbi.nlm.nih.gov/summary/summary.cgi?cid=50986185) | (+)-Cubenene | C[C@H]1CC[C@@H]([C@@H]2C1=CCC(=C2)C)C(C)C |
| ZS207 | delta-elemene | MOL001608 | [12309449](http://pubchem.ncbi.nlm.nih.gov/summary/summary.cgi?cid=12309449) | delta-Elemene | CC(C)C1=C[C@@H]([C@@](CC1)(C)C=C)C(=C)C |
| ZS208 | NON | MOL001640 | [2969](http://pubchem.ncbi.nlm.nih.gov/summary/summary.cgi?cid=2969) | Decanoic acid | CCCCCCCCCC(=O)O |
| ZS209 | zoomaric acid | MOL001739 | [445638](http://pubchem.ncbi.nlm.nih.gov/summary/summary.cgi?cid=445638) | Palmitoleic acid | CCCCCC/C=C\CCCCCCCC(=O)O |
| ZS210 | Tetracosane | MOL001747 | [12592](http://pubchem.ncbi.nlm.nih.gov/summary/summary.cgi?cid=12592) | Tetracosane | CCCCCCCCCCCCCCCCCCCCCCCC |
| ZS211 | ZINC03860434 | MOL001749 | [7057921](http://pubchem.ncbi.nlm.nih.gov/summary/summary.cgi?cid=7057921) | bis[(2R)-2-ethylhexyl] benzene-1,2-dicarboxylate | CCCC[C@@H](CC)COC(=O)C1=CC=CC=C1C(=O)OC[C@H](CC)CCCC |
| ZS212 | EUG | MOL001752 | [332](http://pubchem.ncbi.nlm.nih.gov/summary/summary.cgi?cid=332) | 2-Methoxy-4-vinylphenol | COC1=C(C=CC(=C1)C=C)O |
| ZS213 | poriferast-5-en-3beta-ol | MOL001771 | [457801](http://pubchem.ncbi.nlm.nih.gov/summary/summary.cgi?cid=457801) | Clionasterol | CC[C@@H](CC[C@@H](C)[C@H]1CC[C@@H]2[C@@]1(CC[C@H]3[C@H]2CC=C4[C@@]3(CC[C@@H](C4)O)C)C)C(C)C |
| ZS214 | Methyl linolelaidate | MOL001889 | [5362793](http://pubchem.ncbi.nlm.nih.gov/summary/summary.cgi?cid=5362793) | Methyl linolelaidate | CCCCC/C=C/C/C=C/CCCCCCCC(=O)OC |
| ZS215 | Pulegone | MOL001972 | [442495](http://pubchem.ncbi.nlm.nih.gov/summary/summary.cgi?cid=442495) | Pulegone | C[C@@H]1CCC(=C(C)C)C(=O)C1 |
| ZS216 | ()-Cuparene | MOL002029 | [86895](http://pubchem.ncbi.nlm.nih.gov/summary/summary.cgi?cid=86895) | Cuparene | CC1=CC=C(C=C1)[C@@]2(CCCC2(C)C)C |
| ZS217 | cis-Thujopsene | MOL002033 | [442402](http://pubchem.ncbi.nlm.nih.gov/summary/summary.cgi?cid=442402) | Thujopsene | CC1=CC[C@@]2(CCCC([C@]23[C@H]1C3)(C)C)C |
| ZS218 | alpha-Cubebene | MOL002085 | N/A | N/A | CC1=CC[C@]23[C@H]1[C@H]2[C@H](C(C)C)CC[C@H]3C |
| ZS219 | Antioxidant No. 33 | MOL002092 | [7311](http://pubchem.ncbi.nlm.nih.gov/summary/summary.cgi?cid=7311) | 2,4-Di-tert-butylphenol | CC(C)(C)C1=CC(=C(C=C1)O)C(C)(C)C |
| ZS220 | DEP | MOL002095 | [6781](http://pubchem.ncbi.nlm.nih.gov/summary/summary.cgi?cid=6781) | Diethyl phthalate | CCOC(=O)C1=CC=CC=C1C(=O)OCC |
| ZS221 | β-sesquiphellandrene | MOL002117 | [12315492](http://pubchem.ncbi.nlm.nih.gov/summary/summary.cgi?cid=12315492) | beta-Sesquiphellandrene | C[C@@H](CCC=C(C)C)[C@H]1CCC(=C)C=C1 |
| ZS222 | 1H-Cycloprop(e)azulen-7-ol, decahydro-1,1,7-trimethyl-4-methylene-, (1aR-(1aalpha,4aalpha,7beta,7abeta,7balpha))- | MOL002153 | [92231](http://pubchem.ncbi.nlm.nih.gov/summary/summary.cgi?cid=92231) | Spathulenol | C[C@@]1(CC[C@@H]2[C@@H]1[C@H]3[C@H](C3(C)C)CCC2=C)O |
| ZS223 | 4-Octanone | MOL002180 | [11516](http://pubchem.ncbi.nlm.nih.gov/summary/summary.cgi?cid=11516) | 4-Octanone | CCCCC(=O)CCC |
| ZS224 | cinnamic acid | MOL002295 | [444539](http://pubchem.ncbi.nlm.nih.gov/summary/summary.cgi?cid=444539) | Cinnamic acid | C1=CC=C(C=C1)/C=C/C(=O)O |
| ZS225 | Homocresol | MOL002334 | [62465](http://pubchem.ncbi.nlm.nih.gov/summary/summary.cgi?cid=62465) | 4-Ethyl-2-methoxyphenol | CCC1=CC(=C(C=C1)O)OC |
| ZS226 | 3-Hexenol | MOL002351 | [5281167](http://pubchem.ncbi.nlm.nih.gov/summary/summary.cgi?cid=5281167) | cis-3-Hexen-1-ol | CC/C=C\CCO |
| ZS227 | Terragon | MOL002361 | [8815](http://pubchem.ncbi.nlm.nih.gov/summary/summary.cgi?cid=8815) | Estragole | COC1=CC=C(C=C1)CC=C |
| ZS228 | Isogeraniol | MOL002362 | [5362876](http://pubchem.ncbi.nlm.nih.gov/summary/summary.cgi?cid=5362876) | Isogeraniol | CC(=CC/C=C(/C)\CCO)C |
| ZS229 | UND | MOL002378 | [14257](http://pubchem.ncbi.nlm.nih.gov/summary/summary.cgi?cid=14257) | Undecane | CCCCCCCCCCC |
| ZS230 | (-)-Comphene | MOL002453 | [440966](http://pubchem.ncbi.nlm.nih.gov/summary/summary.cgi?cid=440966) | (-)-Camphene | CC1(C2CCC(C2)C1=C)C |
| ZS231 | (2R)-2,6-dimethylhept-5-enal | MOL002477 | [6997374](http://pubchem.ncbi.nlm.nih.gov/summary/summary.cgi?cid=6997374) | (R)-2,6-Dimethyl-5-heptenal | C[C@H](CCC=C(C)C)C=O |
| ZS232 | Isovaleral | MOL002484 | [11552](http://pubchem.ncbi.nlm.nih.gov/summary/summary.cgi?cid=11552) | 3-Methylbutanal | CC(C)CC=O |
| ZS233 | Sulcatone | MOL002496 | [9862](http://pubchem.ncbi.nlm.nih.gov/summary/summary.cgi?cid=9862) | 6-Methyl-5-hepten-2-one | CC(=CCCC(=O)C)C |
| ZS234 | Peruviol | MOL002504 | [5356544](http://pubchem.ncbi.nlm.nih.gov/summary/summary.cgi?cid=5356544) | Peruviol | CC(=CCC/C(=C\CC[C@@](C)(C=C)O)/C)C |
| ZS235 | .beta.-Fenchyl acetate, exo- | MOL002520 | [156660](http://pubchem.ncbi.nlm.nih.gov/summary/summary.cgi?cid=156660) | beta-Fenchyl acetate, exo- | CC(=O)O[C@@H]1[C@]2(CC[C@H](C2)C1(C)C)C |
| ZS236 | 3691-11-0 | MOL002526 | [94275](http://pubchem.ncbi.nlm.nih.gov/summary/summary.cgi?cid=94275) | delta-Guaiene | C[C@H]1CCC2=C(CC[C@H](C[C@@H]12)C(=C)C)C |
| ZS237 | trans-p-mentha-1(7),8-diene | MOL002537 | [68140](http://pubchem.ncbi.nlm.nih.gov/summary/summary.cgi?cid=68140) | 1-Methylene-4-(1-methylvinyl)cyclohexane | CC(=C)C1CCC(=C)CC1 |
| ZS238 | Hexenal | MOL002675 | [5281168](http://pubchem.ncbi.nlm.nih.gov/summary/summary.cgi?cid=5281168) | 2-Hexenal | CCC/C=C/C=O |
| ZS239 | EB | MOL002678 | [7500](http://pubchem.ncbi.nlm.nih.gov/summary/summary.cgi?cid=7500) | Ethylbenzene | CCC1=CC=CC=C1 |
| ZS240 | Ligla | MOL002683 | [5280933](http://pubchem.ncbi.nlm.nih.gov/summary/summary.cgi?cid=5280933) | gamma-Linolenic acid | CCCCC/C=C\C/C=C\C/C=C\CCCCC(=O)O |
| ZS241 | PCR | MOL002830 | [2879](http://pubchem.ncbi.nlm.nih.gov/summary/summary.cgi?cid=2879) | P-Cresol | CC1=CC=C(C=C1)O |
| ZS242 | butylated hydroxytoluene | MOL002850 | [31404](http://pubchem.ncbi.nlm.nih.gov/summary/summary.cgi?cid=31404) | Butylated hydroxytoluene | CC1=CC(=C(C(=C1)C(C)(C)C)O)C(C)(C)C |
| ZS243 | Methyl oleate | MOL002875 | [5280590](http://pubchem.ncbi.nlm.nih.gov/summary/summary.cgi?cid=5280590) | Methyl elaidate | CCCCCCCC/C=C/CCCCCCCC(=O)OC |
| ZS244 | (4S)-1-methyl-4-(6-methylhepta-1,5-dien-2-yl)cyclohexene | MOL002972 | [10104370](http://pubchem.ncbi.nlm.nih.gov/summary/summary.cgi?cid=10104370) | beta-Bisabolene | CC1=CC[C@H](CC1)C(=C)CCC=C(C)C |
| ZS245 | Guasol | MOL002983 | [460](http://pubchem.ncbi.nlm.nih.gov/summary/summary.cgi?cid=460) | Guaiacol | COC1=CC=CC=C1O |
| ZS246 | IPH | MOL002998 | [20488062](http://pubchem.ncbi.nlm.nih.gov/summary/summary.cgi?cid=20488062) | Hydron;phenoxide | [H+].C1=CC=C(C=C1)[O-] |
| ZS247 | [(1S)-endo]-(-)-Borneol | MOL003047 | [1201518](http://pubchem.ncbi.nlm.nih.gov/summary/summary.cgi?cid=1201518) | L-Borneol | C[C@]12CC[C@H](C1(C)C)C[C@H]2O |
| ZS248 | nonanoic acid | MOL003050 | [8158](http://pubchem.ncbi.nlm.nih.gov/summary/summary.cgi?cid=8158) | Nonanoic acid | CCCCCCCCC(=O)O |
| ZS249 | Hexadienal | MOL003060 | [637564](http://pubchem.ncbi.nlm.nih.gov/summary/summary.cgi?cid=637564) | 2,4-Hexadienal | C/C=C/C=C/C=O |
| ZS250 | Germacrene D | MOL003127 | [5317570](http://pubchem.ncbi.nlm.nih.gov/summary/summary.cgi?cid=5317570) | (-)-Germacrene D | C/C/1=C\CCC(=C)/C=C/[C@@H](CC1)C(C)C |
| ZS251 | Isobutyral | MOL003416 | [6561](http://pubchem.ncbi.nlm.nih.gov/summary/summary.cgi?cid=6561) | Isobutyraldehyde | CC(C)C=O |
| ZS252 | (E)-4-[(4R)-4-hydroxy-2,6,6-trimethyl-1-cyclohexenyl]but-3-en-2-one | MOL003469 | [11127505](http://pubchem.ncbi.nlm.nih.gov/summary/summary.cgi?cid=11127505) | (3R)-hydroxy-beta-ionone | CC1=C(C(C[C@@H](C1)O)(C)C)/C=C/C(=O)C |
| ZS253 | PEY | MOL003484 | [995](http://pubchem.ncbi.nlm.nih.gov/summary/summary.cgi?cid=995) | Phenanthrene | C1=CC=C2C(=C1)C=CC3=CC=CC=C32 |
| ZS254 | naphthalene | MOL003493 | [931](http://pubchem.ncbi.nlm.nih.gov/summary/summary.cgi?cid=931) | Naphthalene | C1=CC=C2C=CC=CC2=C1 |
| ZS255 | Damascenone | MOL003520 | [5366074](http://pubchem.ncbi.nlm.nih.gov/summary/summary.cgi?cid=5366074) | Damascenone | C/C=C/C(=O)C1=C(C=CCC1(C)C)C |
| ZS256 | CADINENE | MOL003534 | [10657](http://pubchem.ncbi.nlm.nih.gov/summary/summary.cgi?cid=10657) | beta-Cadinene | CC1=CC[C@@H]2[C@@H](C1)[C@@H](CC=C2C)C(C)C |
| ZS257 | T-Cadinol | MOL003536 | [160799](http://pubchem.ncbi.nlm.nih.gov/summary/summary.cgi?cid=160799) | Cedrelanol | CC1=C[C@H]2[C@@H](CC[C@]([C@@H]2CC1)(C)O)C(C)C |
| ZS258 | Azaron | MOL003547 | [636822](http://pubchem.ncbi.nlm.nih.gov/summary/summary.cgi?cid=636822) | alpha-Asarone | C/C=C/C1=CC(=C(C=C1OC)OC)OC |
| ZS259 | Fuseloel | MOL003877 | [31260](http://pubchem.ncbi.nlm.nih.gov/summary/summary.cgi?cid=31260) | Isoamyl alcohol | CC(C)CCO |
| ZS260 | Naphthalene, 1,2,3,4,4a,5,6,8a-octahydro-7-methyl-4-methylene-1-(1-methylethyl)-, (1alpha,4abeta,8aalpha)- | MOL003937 | [92313](http://pubchem.ncbi.nlm.nih.gov/summary/summary.cgi?cid=92313) | (-)-gamma-Cadinene | CC1=C[C@H]2[C@H](CC1)C(=C)CC[C@@H]2C(C)C |
| ZS261 | nerolidyl acetate | MOL003938 | N/A | N/A | C=C[C@@](C)(CCC=C(C)CCC=C(C)C)OC(C)=O |
| ZS262 | Perillen | MOL004294 | [68316](http://pubchem.ncbi.nlm.nih.gov/summary/summary.cgi?cid=68316) | Perillene | CC(=CCCC1=COC=C1)C |
| ZS263 | globulol | MOL004419 | [11996452](http://pubchem.ncbi.nlm.nih.gov/summary/summary.cgi?cid=11996452) | Viridiflorol | C[C@@H]1CC[C@H]2[C@@H]1[C@H]3[C@H](C3(C)C)CC[C@]2(C)O |
| ZS264 | CREOSOL | MOL004483 | [7144](http://pubchem.ncbi.nlm.nih.gov/summary/summary.cgi?cid=7144) | 2-Methoxy-4-methylphenol | CC1=CC(=C(C=C1)O)OC |
| ZS265 | Methyl naphthalene | MOL004582 | [7002](http://pubchem.ncbi.nlm.nih.gov/summary/summary.cgi?cid=7002) | 1-Methylnaphthalene | CC1=CC=CC2=CC=CC=C12 |
| ZS266 | PAC | MOL004629 | [999](http://pubchem.ncbi.nlm.nih.gov/summary/summary.cgi?cid=999) | Phenylacetic acid | C1=CC=C(C=C1)CC(=O)O |
| ZS267 | TDA | MOL004647 | [12530](http://pubchem.ncbi.nlm.nih.gov/summary/summary.cgi?cid=12530) | Tridecanoic acid | CCCCCCCCCCCCC(=O)O |
| ZS268 | tau-cadinol | MOL004707 | [12302227](http://pubchem.ncbi.nlm.nih.gov/summary/summary.cgi?cid=12302227) | (1S)-1,2,3,4,4aalpha,7,8,8aalpha-Octahydro-1,6-dimethyl-4beta-isopropylnaphthalen-1beta-ol | CC1=C[C@@H]2[C@@H](CC[C@]([C@@H]2CC1)(C)O)C(C)C |
| ZS269 | beta-Terpinene | MOL004723 | [66841](http://pubchem.ncbi.nlm.nih.gov/summary/summary.cgi?cid=66841) | beta-Terpinene | CC(C)C1=CCC(=C)CC1 |
| ZS270 | (1R,4aR,8aS)-1-isopropyl-7-methyl-4-methylene-2,3,4a,5,6,8a-hexahydro-1H-naphthalene | MOL004727 | [6432308](http://pubchem.ncbi.nlm.nih.gov/summary/summary.cgi?cid=6432308) | gamma-Muurolene | CC1=C[C@H]2[C@@H](CC1)C(=C)CC[C@@H]2C(C)C |
| ZS271 | CHX | MOL004735 | [8078](http://pubchem.ncbi.nlm.nih.gov/summary/summary.cgi?cid=8078) | Cyclohexane | C1CCCCC1 |
| ZS272 | 2-methyl-6-ethyl decane | MOL004922 | N/A | N/A | CCCCC(CC)CCCC(C)C |
| ZS273 | SKM | MOL005074 | [8742](http://pubchem.ncbi.nlm.nih.gov/summary/summary.cgi?cid=8742) | Shikimic acid | C1[C@H]([C@@H]([C@@H](C=C1C(=O)O)O)O)O |
| ZS274 | Ethylcyclohexane | MOL005268 | [15504](http://pubchem.ncbi.nlm.nih.gov/summary/summary.cgi?cid=15504) | Ethylcyclohexane | CCC1CCCCC1 |
| ZS275 | 5-methyl-tetradecane | MOL005299 | N/A | N/A | CCCCCCCCC[C@H](C)CCCC |
| ZS276 | 1,2-Benzenedicarboxylicacid, mono(2-ethyl) hexylester | MOL005472 | N/A | N/A | CCCC[C@@H](CC)COC(=O)c1ccccc1C(=O)O |
| ZS277 | phytane | MOL005521 | N/A | N/A | CC[C@@H](C)CCC[C@@H](C)CCC[C@H](C)CCCC(C)C |
| ZS278 | Linolenyl alcohol | MOL005538 | [6436081](http://pubchem.ncbi.nlm.nih.gov/summary/summary.cgi?cid=6436081) | Linolenyl alcohol | CC/C=C\C/C=C\C/C=C\CCCCCCCCO |
| ZS279 | 3-methyl-6-(1-methylethylidene)-cyclohexene | MOL005712 | [71385764](http://pubchem.ncbi.nlm.nih.gov/summary/summary.cgi?cid=71385764) | UNII-VR4XVW6V2H component CIPXOBMYVWRNLL-SECBINFHSA-N | C[C@H]1CCC(=C(C)C)C=C1 |
| ZS280 | Pentadecanal | MOL005732 | [17697](http://pubchem.ncbi.nlm.nih.gov/summary/summary.cgi?cid=17697) | Pentadecanal | CCCCCCCCCCCCCCC=O |
| ZS281 | [(4R)-4-isopropenyl-1-cyclohexenyl]methanol | MOL005752 | [11788398](http://pubchem.ncbi.nlm.nih.gov/summary/summary.cgi?cid=11788398) | [(4R)-4-(prop-1-en-2-yl)cyclohex-1-en-1-yl]methanol | CC(=C)[C@@H]1CCC(=CC1)CO |
| ZS282 | 2,6,11-trimethyldodecane | MOL005819 | N/A | N/A | CC(C)CCCC[C@@H](C)CCCC(C)C |
| ZS283 | Tormentic acid | MOL005855 | [73193](http://pubchem.ncbi.nlm.nih.gov/summary/summary.cgi?cid=73193) | Tormentic acid | C[C@@H]1CC[C@@]2(CC[C@@]3(C(=CC[C@H]4[C@]3(CC[C@@H]5[C@@]4(C[C@H]([C@@H](C5(C)C)O)O)C)C)[C@@H]2[C@]1(C)O)C)C(=O)O |
| ZS284 | 16-Heptadecenal | MOL005962 | [557527](http://pubchem.ncbi.nlm.nih.gov/summary/summary.cgi?cid=557527) | 16-Heptadecenal | C=CCCCCCCCCCCCCCCC=O |
| ZS285 | Nonadecylic acid | MOL005971 | [12591](http://pubchem.ncbi.nlm.nih.gov/summary/summary.cgi?cid=12591) | Nonadecanoic acid | CCCCCCCCCCCCCCCCCCC(=O)O |
| ZS286 | δ-selinene | MOL006292 | [12308845](http://pubchem.ncbi.nlm.nih.gov/summary/summary.cgi?cid=12308845) | (-)-delta-Selinene | CC1=C2C=C(CC[C@@]2(CCC1)C)C(C)C |
| ZS287 | Furfuranol | MOL006735 | [7361](http://pubchem.ncbi.nlm.nih.gov/summary/summary.cgi?cid=7361) | Furfuryl alcohol | C1=COC(=C1)CO |
| ZS288 | Methylpyrazine | MOL006939 | [7976](http://pubchem.ncbi.nlm.nih.gov/summary/summary.cgi?cid=7976) | 2-Methylpyrazine | CC1=NC=CN=C1 |
| ZS289 | Linolenic acid ethyl ester | MOL007179 | [5367460](http://pubchem.ncbi.nlm.nih.gov/summary/summary.cgi?cid=5367460) | Ethyl linolenate | CC/C=C\C/C=C\C/C=C\CCCCCCCC(=O)OCC |
| ZS290 | MENTHOL | MOL007330 | [16666](http://pubchem.ncbi.nlm.nih.gov/summary/summary.cgi?cid=16666) | l-Menthol | C[C@@H]1CC[C@H]([C@@H](C1)O)C(C)C |
| ZS291 | 2-Butyl-4-hydroxyanisole | MOL007331 | [8456](http://pubchem.ncbi.nlm.nih.gov/summary/summary.cgi?cid=8456) | 2-tert-Butyl-4-methoxyphenol | CC(C)(C)C1=C(C=CC(=C1)OC)O |
| ZS292 | 2,5-ditert-butylphenol | MOL007339 | [79983](http://pubchem.ncbi.nlm.nih.gov/summary/summary.cgi?cid=79983) | 2,5-Di-tert-butylphenol | CC(C)(C)C1=CC(=C(C=C1)C(C)(C)C)O |
| ZS293 | NaPst | MOL007497 | [7406](http://pubchem.ncbi.nlm.nih.gov/summary/summary.cgi?cid=7406) | Cumene | CC(C)C1=CC=CC=C1 |
| ZS294 | methyl icosa-11,14-dienoate | MOL007514 | [5365566](http://pubchem.ncbi.nlm.nih.gov/summary/summary.cgi?cid=5365566) | 11,14-Eicosadienoic acid, methyl ester | CCCCC/C=C/C/C=C/CCCCCCCCCC(=O)OC |
| ZS295 | (-)-beta-Fenchol | MOL007553 | [61123](http://pubchem.ncbi.nlm.nih.gov/summary/summary.cgi?cid=61123) | (-)-beta-Fenchyl alcohol | C[C@]12CC[C@H](C1)C([C@@H]2O)(C)C |
| ZS296 | (1S,2R,4R)-2-methyl-3-methylene-2-(4-methylpent-3-enyl)norbornane | MOL007554 | [10889018](http://pubchem.ncbi.nlm.nih.gov/summary/summary.cgi?cid=10889018) | beta-Santalene | CC(=CCC[C@@]1([C@H]2CC[C@H](C2)C1=C)C)C |
| ZS297 | Hexyl formate | MOL007887 | [61177](http://pubchem.ncbi.nlm.nih.gov/summary/summary.cgi?cid=61177) | Hexyl formate | CCCCCCOC=O |
| ZS298 | 2-Hexanoylfuran | MOL007934 | [61738](http://pubchem.ncbi.nlm.nih.gov/summary/summary.cgi?cid=61738) | 2-Hexanoylfuran | CCCCCC(=O)C1=CC=CO1 |
| ZS299 | α-patchoulene | MOL007935 | N/A | N/A | CC1=CC[C@@H]2C[C@H]3[C@H](C)CC[C@]13C2(C)C |
| ZS300 | 19435-97-3 | MOL007946 | [3084311](http://pubchem.ncbi.nlm.nih.gov/summary/summary.cgi?cid=3084311) | delta-Cadinol | CC1=C[C@H]2[C@@H](CC[C@@]([C@H]2CC1)(C)O)C(C)C |
| ZS301 | ZINC02011663 | MOL008138 | [445789](http://pubchem.ncbi.nlm.nih.gov/summary/summary.cgi?cid=445789) | (3R)-octan-3-ol | CCCCC[C@@H](CC)O |
| ZS302 | Tritriacontane | MOL008236 | [12411](http://pubchem.ncbi.nlm.nih.gov/summary/summary.cgi?cid=12411) | Tritriacontane | CCCCCCCCCCCCCCCCCCCCCCCCCCCCCCCCC |
| ZS303 | DODECENE | MOL008245 | [8183](http://pubchem.ncbi.nlm.nih.gov/summary/summary.cgi?cid=8183) | 1-Dodecene | CCCCCCCCCCC=C |
| ZS304 | m-Ethylphenol | MOL008292 | [12101](http://pubchem.ncbi.nlm.nih.gov/summary/summary.cgi?cid=12101) | 3-Ethylphenol | CCC1=CC(=CC=C1)O |
| ZS305 | Vinyl amyl ketone | MOL008661 | [61346](http://pubchem.ncbi.nlm.nih.gov/summary/summary.cgi?cid=61346) | 1-Octen-3-one | CCCCCC(=O)C=C |
| ZS306 | ZINC00901303 | MOL008672 | [6971249](http://pubchem.ncbi.nlm.nih.gov/summary/summary.cgi?cid=6971249) | (S)-2-methylbutanal | CC[C@H](C)C=O |
| ZS307 | 1,2-benzothiazole | MOL008769 | 9225 | 1,2-Benzisothiazole | C1=CC=C2C(=C1)C=NS2 |
| ZS308 | Butylbenzene | MOL008777 | [7705](http://pubchem.ncbi.nlm.nih.gov/summary/summary.cgi?cid=7705) | Butylbenzene | CCCCC1=CC=CC=C1 |
| ZS309 | m-Tolualdehyde | MOL009552 | [12105](http://pubchem.ncbi.nlm.nih.gov/summary/summary.cgi?cid=12105) | 3-Methylbenzaldehyde | CC1=CC(=CC=C1)C=O |
| ZS310 | (1S,4aS,6S,8aR)-1,6-dimethyldecalin | MOL009610 | [21718000](http://pubchem.ncbi.nlm.nih.gov/summary/summary.cgi?cid=21718000) | (1S,4aS,6S,8aR)-1,6-dimethyl-1,2,3,4,4a,5,6,7,8,8a-decahydronaphthalene | C[C@H]1CC[C@@H]2[C@H](CCC[C@H]2C1)C |
| ZS311 | Prehnitol | MOL009852 | [10263](http://pubchem.ncbi.nlm.nih.gov/summary/summary.cgi?cid=10263) | 1,2,3,4-Tetramethylbenzene | C[C@H]1CC[C@H](C2=C1C=CC(=C2)C)C(C)C |
| ZS312 | 2-Hexenol | MOL010610 | [5318042](http://pubchem.ncbi.nlm.nih.gov/summary/summary.cgi?cid=5318042) | CCCC=CCO | CCC/C=C/CO |
| ZS313 | (Z)-calamenene | MOL010947 | [6429077](http://pubchem.ncbi.nlm.nih.gov/summary/summary.cgi?cid=6429077) | Calamenene | C[C@H]1CC[C@H](C2=C1C=CC(=C2)C)C(C)C |
| ZS314 | bergamotene (Z,.alpha.,cis) | MOL011148 | [6429302](http://pubchem.ncbi.nlm.nih.gov/summary/summary.cgi?cid=6429302) | trans-alpha-Bergamotene | CC1=CC[C@H]2C[C@@H]1[C@]2(C)CCC=C(C)C |
| ZS315 | VCA | MOL011320 | [5282761](http://pubchem.ncbi.nlm.nih.gov/summary/summary.cgi?cid=5282761) | cis-Vaccenic acid | CCCCCC/C=C\CCCCCCCCCC(=O)O |
| ZS316 | ZINC02140511 | MOL011544 | N/A | N/A | C=C1[C@H]2CC[C@@H]3[C@H]2C(C)(C)CCC[C@]13C |
| ZS317 | gamma-Hexenol | MOL011563 | [5284503](http://pubchem.ncbi.nlm.nih.gov/summary/summary.cgi?cid=5284503) | trans-3-Hexen-1-ol | CC/C=C/CCO |
| ZS318 | 2-Aminoimidazole | MOL011652 | [82140](http://pubchem.ncbi.nlm.nih.gov/summary/summary.cgi?cid=82140) | 1H-Imidazol-2-amine | C1=CN=C(N1)N |
| ZS319 | 7-octen-4-ol | MOL011756 | N/A | N/A | C=CCC[C@H](O)CCC |
| ZS320 | 4-methoxy-6-[(E)-prop-1-enyl]-1,3-benzodioxole | MOL011777 | [6436484](http://pubchem.ncbi.nlm.nih.gov/summary/summary.cgi?cid=6436484) | Isomyristicin | C/C=C/C1=CC2=C(C(=C1)OC)OCO2 |
| ZS321 | EAK | MOL011840 | [246728](http://pubchem.ncbi.nlm.nih.gov/summary/summary.cgi?cid=246728) | 3-Octanone | CCCCCC(=O)CC |
| ZS322 | d-Piperitone | MOL011848 | [61362](http://pubchem.ncbi.nlm.nih.gov/summary/summary.cgi?cid=61362) | d-Piperitone | CC1=CC(=O)[C@@H](CC1)C(C)C |
| ZS323 | (1Z,4E,8E)-2,6,6,9-tetramethylcycloundeca-1,4,8-triene | MOL012439 | [5318101](http://pubchem.ncbi.nlm.nih.gov/summary/summary.cgi?cid=5318101) | (1Z,4E,8E)-2,6,6,9-tetramethylcycloundeca-1,4,8-triene | CC1=CCC(C=CCC(=CCC1)C)(C)C |
| ZS324 | cis-nerolidol | MOL012609 | [12227246](http://pubchem.ncbi.nlm.nih.gov/summary/summary.cgi?cid=12227246) | 1,6,10-Dodecatrien-3-ol, 3,7,11-trimethyl-, (3R,6Z)- | CC(=CCCC(=CCCC(C)(C=C)O)C)C |
| ZS325 | caryophellene | MOL012644 | [5498518](http://pubchem.ncbi.nlm.nih.gov/summary/summary.cgi?cid=5498518) | Caryophyllene (z-) | C/C/1=C/CCC(=C)[C@H]2CC([C@H]2CC1)(C)C |
| ZS326 | 3,5-Octadien-2-one, (E,E)- | MOL013022 | [5352876](http://pubchem.ncbi.nlm.nih.gov/summary/summary.cgi?cid=5352876) | (E,E)-3,5-octadien-2-one | CCC=CC=CC(=O)C |
| ZS327 | 1,7-Octadien-3-ol,3,7-dimethyl | MOL013261 | N/A | 1,7-Octadien-3-ol,3,7-dimethyl | C=C[C@](C)(O)CCCC(=C)C |
| ZS328 | .alpha.-Myrcene | MOL013263 | [519324](http://pubchem.ncbi.nlm.nih.gov/summary/summary.cgi?cid=519324) | alpha-Myrcene | CC(=C)CCCC(=C)C=C |

# Table S3. The interactions between the top 10 active Perillae Folium compounds for each target and all of the 25 candidate targets.

| **Compound code** | **Degree** | **Compound code** | **Degree** |
| --- | --- | --- | --- |
| ZS059 | 21 | ZS026 | 6 |
| ZS058 | 21 | ZS024 | 4 |
| ZS074 | 19 | ZS067 | 3 |
| ZS014 | 17 | ZS042 | 3 |
| ZS070 | 15 | ZS084 | 2 |
| ZS118 | 15 | ZS073 | 2 |
| ZS083 | 14 | ZS060 | 2 |
| ZS076 | 14 | ZS025 | 2 |
| ZS036 | 14 | ZS011 | 1 |
| ZS066 | 13 | ZS039 | 1 |
| ZS055 | 10 | ZS213 | 1 |
| ZS068 | 10 | ZS179 | 1 |
| ZS283 | 10 | ZS253 | 1 |
| ZS057 | 8 | ZS201 | 1 |
| ZS065 | 8 | ZS037 | 1 |
| ZS125 | 8 | ZS270 | 1 |

Note: The degree value indicates how many targets a compound is able to bind with. Corresponding compound names could refer to Table S1.

# Table S4. Similarity scores of the compounds compared with febuxostat.

| **Compound code** | **Similarity scores** |
| --- | --- |
| ZS025+ | 0.50877 |
| ZS024+ | 0.491123 |
| ZS031+ | 0.49068 |
| ZS134+ | 0.47305 |
| ZS084+ | 0.46784 |
| ZS025- | 0.50877 |
| ZS093- | 0.41916 |
| ZS252- | 0.19431 |
| ZS056- | 0.19066 |
| ZS090- | 0.13808 |

Note: Compounds marked with “+” are those with the top five similarity scores. Compounds with “-” are the five inhibitors identified in the active sites of xanthine dehydrogenase. Corresponding compound names could refer to Table S1.

# Table S5. Classifications of compounds from seven different clusters.

| **Cluster** | **Compound code** | **Class** | **Subclass** |
| --- | --- | --- | --- |
| **Cluster 1**  **(n=37)** | ZS184 | Prenol lipids | Sesquiterpenoids |
|  | ZS091 | Prenol lipids | Sesquiterpenoids |
|  | ZS012 | Prenol lipids | Sesquiterpenoids |
|  | ZS169 | Prenol lipids | Sesquiterpenoids |
|  | ZS065 | Steroids and steroid derivatives | Pregnane steroids |
|  | ZS109 | Prenol lipids | Sesquiterpenoids |
|  | ZS139 | Prenol lipids | Sesquiterpenoids |
|  | ZS316 | Prenol lipids | Sesquiterpenoids |
|  | ZS177 | Polycyclic hydrocarbons | NA |
|  | ZS187 | Prenol lipids | Sesquiterpenoids |
|  | ZS296 | Prenol lipids | Sesquiterpenoids |
|  | ZS230 | Prenol lipids | Monoterpenoids |
|  | ZS105 | Prenol lipids | Monoterpenoids |
|  | ZS168 | Prenol lipids | Monoterpenoids |
|  | ZS132 | Prenol lipids | Sesquiterpenoids |
|  | ZS120 | Prenol lipids | Sesquiterpenoids |
|  | ZS218 | Prenol lipids | Sesquiterpenoids |
|  | ZS217 | Prenol lipids | Sesquiterpenoids |
|  | ZS205 | Prenol lipids | Sesquiterpenoids |
|  | ZS133 | Prenol lipids | Sesquiterpenoids |
|  | ZS089 | Prenol lipids | Sesquiterpenoids |
|  | ZS314 | Prenol lipids | Monoterpenoids |
|  | ZS128 | Prenol lipids | Monoterpenoids |
|  | ZS104 | Prenol lipids | Monoterpenoids |
|  | ZS171 | Prenol lipids | Monoterpenoids |
|  | ZS129 | Prenol lipids | Sesquiterpenoids |
|  | ZS186 | Prenol lipids | Sesquiterpenoids |
|  | ZS260 | Prenol lipids | Sesquiterpenoids |
|  | ZS270 | Prenol lipids | Sesquiterpenoids |
|  | ZS206 | Prenol lipids | Sesquiterpenoids |
|  | ZS135 | Prenol lipids | Sesquiterpenoids |
|  | ZS201 | Prenol lipids | Sesquiterpenoids |
|  | ZS325 | Prenol lipids | Sesquiterpenoids |
|  | ZS256 | Prenol lipids | Sesquiterpenoids |
|  | ZS299 | Prenol lipids | Sesquiterpenoids |
|  | ZS236 | Prenol lipids | Sesquiterpenoids |
|  | ZS019 | Prenol lipids | Sesquiterpenoids |
| **Cluster 2**  **(n=12)** | ZS210 | Saturated hydrocarbons | Alkanes |
|  | ZS161 | Saturated hydrocarbons | Alkanes |
|  | ZS160 | Saturated hydrocarbons | Alkanes |
|  | ZS159 | Saturated hydrocarbons | Alkanes |
|  | ZS158 | Saturated hydrocarbons | Alkanes |
|  | ZS157 | Saturated hydrocarbons | Alkanes |
|  | ZS156 | Saturated hydrocarbons | Alkanes |
|  | ZS131 | Saturated hydrocarbons | Alkanes |
|  | ZS229 | Saturated hydrocarbons | Alkanes |
|  | ZS302 | Saturated hydrocarbons | Alkanes |
|  | ZS197 | Saturated hydrocarbons | Alkanes |
|  | ZS194 | Saturated hydrocarbons | Alkanes |
| **Cluster 3**  **(n=12)** | ZS155 | Fatty Acyls | Fatty acids and conjugates |
|  | ZS123 | Fatty Acyls | Fatty acids and conjugates |
|  | ZS095 | Fatty Acyls | Fatty acids and conjugates |
|  | ZS002 | Fatty Acyls | Fatty acids and conjugates |
|  | ZS193 | Fatty Acyls | Fatty acids and conjugates |
|  | ZS195 | Fatty Acyls | Fatty acids and conjugates |
|  | ZS208 | Fatty Acyls | Fatty acids and conjugates |
|  | ZS248 | Fatty Acyls | Fatty acids and conjugates |
|  | ZS267 | Fatty Acyls | Fatty acids and conjugates |
|  | ZS285 | Fatty Acyls | Fatty acids and conjugates |
|  | ZS192 | Fatty Acyls | Fatty acids and conjugates |
|  | ZS124 | Fatty Acyls | Fatty acids and conjugates |
| **Cluster 4**  **(n=9)** | ZS108 | Fatty Acyls | Lineolic acids and derivatives |
|  | ZS053 | Fatty Acyls | Fatty acids and conjugates |
|  | ZS032 | Fatty Acyls | Lineolic acids and derivatives |
|  | ZS209 | Fatty Acyls | Fatty acids and conjugates |
|  | ZS190 | Fatty Acyls | Fatty acids and conjugates |
|  | ZS315 | Fatty Acyls | Fatty acids and conjugates |
|  | ZS140 | Fatty Acyls | Fatty acids and conjugates |
|  | ZS035 | Fatty Acyls | Fatty acids and conjugates |
|  | ZS240 | Fatty Acyls | Lineolic acids and derivatives |
| **Cluster 5**  **(n=9)** | ZS200 | Prenol lipids | Triterpenoids |
|  | ZS175 | Prenol lipids | Sesquiterpenoids |
|  | ZS086 | Prenol lipids | Sesquiterpenoids |
|  | ZS152 | Prenol lipids | Monoterpenoids |
|  | ZS233 | Organooxygen compounds | Carbonyl compounds |
|  | ZS044 | Prenol lipids | Monoterpenoids |
|  | ZS174 | Prenol lipids | Sesquiterpenoids |
|  | ZS034 | Prenol lipids | Sesquiterpenoids |
|  | ZS009 | Prenol lipids | Monoterpenoids |
| **Cluster 6**  **(n=7)** | ZS042 | Cinnamic acids and derivatives | Hydroxycinnamic acids and derivatives |
|  | ZS027 | Cinnamic acids and derivatives | Hydroxycinnamic acids and derivatives |
|  | ZS075 | Cinnamic acids and derivatives | Hydroxycinnamic acids and derivatives |
|  | ZS041 | Cinnamic acids and derivatives | Hydroxycinnamic acids and derivatives |
|  | ZS063 | Cinnamic acids and derivatives | Hydroxycinnamic acids and derivatives |
|  | ZS029 | Cinnamic acids and derivatives | Hydroxycinnamic acids and derivatives |
|  | ZS016 | Cinnamic acids and derivatives | Hydroxycinnamic acids and derivatives |
| **Cluster 7**  **(n=7)** | ZS225 | Phenols | Methoxyphenols |
|  | ZS113 | Phenols | Methoxyphenols |
|  | ZS212 | Phenols | Methoxyphenols |
|  | ZS114 | Benzene and substituted derivatives | Methoxyphenols |
|  | ZS264 | Phenols | Methoxyphenols |
|  | ZS020 | Phenols | Methoxyphenols |
|  | ZS023 | Phenol ethers | Anisoles |

Note: Corresponding compound names could refer to Table S1.

# Table S6. Details of 37 compounds identified in the active sites of xanthine dehydrogenase.

| **Compound code** | **Molecule name** | **Mol ID** | **Conventional H-bond formed at the four active sites** | **Conventional H-bond formed at other sites** | **Unfavorable donor-donor bonds** | **Binding affinity (kcal/mol)** | **H-HT** | **PAINS** | **Lipinski rules**  **of five** |  |
| --- | --- | --- | --- | --- | --- | --- | --- | --- | --- | --- |
| ZS004 | d-mandelonitrile | MOL001321 | 0 | 2(Lys1046, Ala1084) | 0 | -5.72 | 0.178 | 0 | Accepted |  |
| ZS007 | α-cubebol | MOL001599 | 0 | 0 | 0 | -6.66 | 0.261 | 0 | Accepted |  |
| ZS018 | (Z,Z)-farnesol | MOL000247 | 0 | 1(Glu1262) | 0 | -5.84 | 0.794 | 0 | Accepted |  |
| ZS025^*^ | scutellarein | MOL002737 | 0 | 2(Ala1080, Lys1046) | 0 | -7.85 | 0.086 | 0 | Accepted |  |
| ZS044 | Citralva | MOL006193 | 0 | 2(Ser1083, Ala1084) | 0 | -5.29 | 0.895 | 0 | Accepted |  |
| ZS056^*^ | Benzyl alpha-D-mannopyranoside | MOL006205 | 0 | 2(Gln768, Gln1195) | 0 | -6.86 | 0.044 | 0 | Accepted |  |
| ZS080 | perilloside c_qt | MOL006230 | 0 | 0 | 0 | -5.47 | 0.369 | 0 | Accepted |  |
| ZS090^*^ | 2-[(1R,3S,4S)-3-isopropenyl-4-methyl-4-vinylcyclohexyl]propan-2-ol | MOL000034 | 0 | 1(Ser1081) | 0 | -6.3 | 0.082 | 0 | Accepted |  |
| ZS093^*^ | DIBP | MOL000057 | 0 | 2(Ser1081, Gln1041) | 0 | -6.4 | 0.008 | 0 | Accepted |  |
| ZS094 | 2-[(2R,5S,6S)-6,10-dimethylspiro[4.5]dec-9-en-2-yl]propan-2-ol | MOL000058 | 0 | 0 | 0 | -6.74 | 0.368 | 0 | Accepted |  |
| ZS096 | Aldrich | MOL000077 | 0 | 1(Gln768) | 0 | -5.19 | 0.085 | 0 | Accepted |  |
| ZS108 | EIC | MOL000131 | 0 | 1(Glu1262) | 0 | -5.52 | 0.174 | 0 | Accepted |  |
| ZS116 | farnesol | MOL000252 | 0 | 0 | 0 | -6.11 | 0.701 | 0 | Accepted |  |
| ZS127 | anethole | MOL000475 | 0 | 1(Ala1080) | 0 | -5.69 | 0.082 | 0 | Accepted |  |
| ZS136 | hexanal | MOL000666 | 1 (1011) | 1(Val1012) | 0 | -4.09 | 0.027 | 0 | Accepted |  |
| ZS140 | oleic acid | MOL000675 | 0 | 2(Ala1080, Glu1262) | 0 | -5.23 | 0.05 | 0 | Accepted |  |
| ZS150 | Hyacinthin | MOL000714 | 0 | 1(Ala1080) | 0 | -5.38 | 0.059 | 0 | Accepted |  |
| ZS165 | Azulen | MOL000884 | 0 | 0 | 0 | -6.25 | 0.049 | 0 | Accepted |  |
| ZS192 | 9-Oxononanoic acid | MOL001385 | 0 | 0 | 1(Gln1195) | -4.97 | 0.037 | 0 | Accepted |  |
| ZS195 | PENTADECYLIC ACID | MOL001396 | 0 | 0 | 1(Gln1195) | -5.2 | 0.028 | 0 | Accepted |  |
| ZS209 | zoomaric acid | MOL001739 | 0 | 0 | 1(Gln768) | -5.2 | 0.054 | 0 | Accepted |  |
| ZS214 | Methyl linolelaidate | MOL001889 | 0 | 0 | 0 | -5.34 | 0.278 | 0 | Accepted |  |
| ZS232 | Isovaleral | MOL002484 | 0 | 0 | 0 | -3.92 | 0.038 | 0 | Accepted |  |
| ZS238 | Hexenal | MOL002675 | 0 | 1(Ala1080) | 0 | -4.28 | 0.082 | 0 | Accepted |  |
| ZS239 | EB | MOL002678 | 0 | 0 | 0 | -5.26 | 0.041 | 0 | Accepted |  |
| ZS240 | Ligla | MOL002683 | 0 | 1(Lys1046) | 1(Gly1261) | -5.76 | 0.221 | 0 | Accepted |  |
| ZS249 | Hexadienal | MOL003060 | 0 | 1(Gln768) | 0 | -4.44 | 0.547 | 0 | Accepted |  |
| ZS252^*^ | (E)-4-[(4R)-4-hydroxy-2,6,6-trimethyl-1-cyclohexenyl]but-3-en-2-one | MOL003469 | 0 | 2(913, Gln1195) | 1(Ser1081) | -6.42 | 0.311 | 0 | Accepted |  |
| ZS262 | Perillen | MOL004294 | 0 | 2(Gln768,800) | 0 | -5.46 | 0.674 | 0 | Accepted |  |
| ZS267 | TDA | MOL004647 | 0 | 1(Lys1046) | 1(Val1260) | -5.2 | 0.031 | 0 | Accepted |  |
| ZS270 | (1R,4aR,8aS)-1-isopropyl-7-methyl-4-methylene-2,3,4a,5,6,8a-hexahydro-1H-naphthalene | MOL004727 | 0 | 0 | 0 | -6.53 | 0.219 | 0 | Accepted |  |
| ZS272 | 2-methyl-6-ethyl decane | MOL004922 | 0 | 0 | 0 | -5.08 | 0.019 | 0 | Accepted |  |
| ZS278 | Linolenyl alcohol | MOL005538 | 0 | 1(Val1260) | 0 | -5.55 | 0.39 | 0 | Accepted |  |
| ZS280 | Pentadecanal | MOL005732 | 0 | 0 | 0 | -4.86 | 0.013 | 0 | Accepted |  |
| ZS282 | 2,6,11-trimethyldodecane | MOL005819 | 0 | 0 | 0 | -5.34 | 0.02 | 0 | Accepted |  |
| ZS291 | 2-Butyl-4-hydroxyanisole | MOL007331 | 0 | 1(Ser1081) | 0 | -5.8 | 0.038 | 0 | Accepted |  |
| ZS309 | m-Tolualdehyde | MOL009552 | 0 | 1(Ser877) | 0 | -5.5 | 0.028 | 0 | Accepted |  |

Note: The compounds marked with “*” are the focused inhibitors against HUA. H-bonds, hydrogen bonds; H-HT, the human hepatotoxicity; PAINS, pan assay interference compounds.

# Table S7. The search strategy for text-mining.

| **Search** | **Query** |
| --- | --- |
| #13 | #10 AND (#12 OR #13) |
| #12 | #8 OR #9 |
| #11 | #6 OR #7 |
| #10 | #1 OR #2 OR #3 OR #4 OR #5 |
| #9 | Xanthine Dehydrogenase [Mesh] |
| #8 | Xanthine Oxidase [Mesh] |
| #7 | Uric Acid [Mesh] |
| #6 | Hyperuricemia [Mesh] |
| #5 | (3R)-hydroxy-beta-ionone- Schema: all |
| #4 | Diisobutyl phthalate |
| #3 | Elemol |
| #2 | Benzyl alpha-D-mannopyranoside |
| #1 | Scutellarein |

# Table S8. List of the excluded articles with reasons.

| **Reasons for exclusions** | **Study ID** | **References** |
| --- | --- | --- |
| Not included in vivo or in vitro sections (n=3) | Rahaman et al., 2022 | Rahaman, M. S., et al. (2022). Crosstalk between xanthine oxidase (XO) inhibiting and cancer chemotherapeutic properties of comestible flavonoids- a comprehensive update. Journal of Nutritional Biochemistry 110. |
|  | Orhan et al., 2021 | Orhan, I. E. and F. S. S. Deniz (2021). Natural Products and Extracts as Xantine Oxidase Inhibitors - A Hope for Gout Disease? Curr Pharm Des 27(2): 143-158. |
|  | Catarino et al., 2015 | Catarino, M. D., et al. (2015). Antioxidant capacities of Flavones and benefits in Oxidative-Stress related Diseases. Current Topics in Medicinal Chemistry 15(2): 105-119. |
|  |  |  |
| Not used the five compounds as an invention (n=3) | Pereira et al., 2018 | Pereira, O. R., et al. (2018). Salvia elegans, salvia greggii and salvia officinalis decoctions: Antioxidant activities and inhibition of carbohydrate and lipid metabolic enzymes. Molecules 23(12). |
|  | Liu H et al., 2003 | Liu, H., et al. (2003). Protective effects of scutellarin on superoxide-induced oxidative stress in rat cortical synaptosomes. Acta Pharmacol Sin 24(11): 1113-1117+1174. |
|  | Yang et al., 2003 | Yang, X. F., et al. (2003). Effects of scutellarin on liver function after brain ischemia/reperfusion in rats. Acta Pharmacol Sin 24(11): 1118-1124+1174. |
|  |  |  |
| 2.Not published in English (n=1) | Liu et al., 2002 | Liu, H., et al. (2002). Study on effects of scutellarin on scavenging reactive oxygen. Zhong yao cai = Zhongyaocai = Journal of Chinese medicinal materials 25(7): 491-493. |

# Table S9. Characteristics of the included papers for the literature-mining section.

| **Study ID** | **Compound** | **Study type** | **Experimental assay** | **Primary results** |
| --- | --- | --- | --- | --- |
| Kim et al, 2017 [1] | Scutellarein | *in vivo* and *in vitro* | Xanthine oxidase inhibitory activity assay | Scutellarein exhibited potent inhibitory activity against xanthine oxidase with IC50 values = 1.74 (1.63–1.92) µM. |
| Liu et al, 2017 [2] | Scutellarein | *in vitro* | Xanthine oxidase inhibitory activity assay | Scutellarein exhibited moderate inhibitory activity against xanthine oxidase with IC50 values of 48.66 µM. |
| Sanz et al, 1994  [3] | Scutellarein | *in vivo* and *in vitro* | Xanthine oxidase inhibitory activity assay | Scutellarein exhibited inhibitory activity against xanthine oxidase with IC50 values = 17.7 ± 1.9 µM. |

# Table S10. The mass spectrometry results of Perillae Folium granules mixture.

| **Alignment ID** | **ESI mode** | **RT (min)** | **m/z** | **Metabolite name** | **Formula** | **Peak area** |
| --- | --- | --- | --- | --- | --- | --- |
| Pos_00153 | Positive | 6.74025 | 177.0909 | Zingerone | C11H14O3 | 157257680 |
| Pos_00109 | Positive | 7.3391 | 201.1637 | Zerumbone | C15H22O | 263431728 |
| Pos_00032 | Positive | 0.8800833 | 319.1499 | Zearalenone | C18H22O5 | 133789144 |
| Neg_00342 | Negative | 0.9606333 | 149.045 | Xylose | C5H10O5 | 317814528 |
| Neg_00198 | Negative | 0.93275 | 151.0603 | Xylitol | C5H12O5 | 366145440 |
| Pos_00760 | Positive | 2.3508 | 251.0642 | Xanthyletin | C14H12O3 | 226327600 |
| Pos_00277 | Positive | 2.655883 | 153.0408 | Xanthine | C5H4N4O2 | 175649760 |
| Neg_00101 | Negative | 7.487817 | 211.134 | Vulgarole | C12H20O3 | 267926368 |
| Pos_00734 | Positive | 5.416467 | 286.2014 | Viridiflorine | C15H27NO4 | 150305552 |
| Pos_00124 | Positive | 0.7128834 | 151.0354 | Vanillate | C8H8O4 | 24231600128 |
| Neg_00059 | Negative | 6.2279 | 167.0343 | Vanillate | C8H8O4 | 305761760 |
| Pos_00343 | Positive | 3.1413 | 245.0766 | Uridine | C9H12N2O6 | 692853376 |
| Neg_00230 | Negative | 3.147683 | 243.0633 | Uridine | C9H12N2O6 | 4815248896 |
| Pos_00598 | Positive | 1.976533 | 169.0358 | Urate | C5H4N4O3 | 1826739840 |
| Neg_00222 | Negative | 1.966467 | 167.0205 | Urate | C5H4N4O3 | 1563959808 |
| Pos_00505 | Positive | 3.1413 | 113.0348 | Uracil | C4H4N2O2 | 3842699776 |
| Neg_00318 | Negative | 2.4942 | 323.0302 | Ump | C9H13N2O9P | 510425440 |
| Neg_00354 | Negative | 7.850867 | 161.0236 | Umbelliferone | C9H6O3 | 5635041280 |
| Pos_00251 | Positive | 2.212167 | 182.0812 | Tyrosine\|2-Amino-3-(4-Hydroxyphenyl)Propanoic Acid | C9H11NO3 | 13357786112 |
| Pos_00278 | Positive | 2.752883 | 182.0812 | Tyrosine | C9H11NO3 | 7179302400 |
| Neg_00224 | Negative | 7.3482 | 136.0759 | Tyramine | C8H11NO | 106999296 |
| Pos_00515 | Positive | 1.227683 | 360.1497 | Turanose | C12H22O11 | 600668416 |
| Pos_00713 | Positive | 6.754183 | 308.185 | Tulipinolide | C17H22O4 | 89660448 |
| Pos_00201 | Positive | 4.971717 | 149.0598 | Tropate | C9H10O3 | 192021200 |
| Pos_00209 | Positive | 11.13025 | 732.5488 | Trimethyl(2-{[(2R)-3-[(9Z)-Octadec-9-Enoyloxy]-2-(Tetradecanoyloxy)Propyl Phosphonato]Oxy}Ethyl)Azanium | C40H78NO8P | 18479926 |
| Pos_00179 | Positive | 11.2709 | 786.601 | Trimethyl(2-{[(2R)-2-[(9Z,12Z)-Octadeca-9,12-Dienoyloxy]-3-(Octadecanoyloxy)Propyl Phosphonato]Oxy}Ethyl)Azanium | C44H84NO8P | 42621012 |
| Pos_00199 | Positive | 6.782017 | 227.1644 | Tridecanedioic Acid | C13H24O4 | 427000128 |
| Neg_00330 | Negative | 6.564 | 301.0367 | Tricetin | C15H10O7 | 89799408 |
| Pos_00268 | Positive | 4.971717 | 131.0491 | Trans-Cinnamate | C9H8O2 | 175330064 |
| Neg_00315 | Negative | 5.1246 | 179.0346 | Trans-Caffeic Acid | C9H8O4 | 1174262272 |
| Pos_00578 | Positive | 5.569417 | 344.1338 | Trans-Beta-D-Glucosyl-2-Hydroxycinnamate | C15H18O8 | 191771872 |
| Neg_00339 | Negative | 5.7798 | 325.0941 | Trans-Beta-D-Glucosyl-2-Hydroxycinnamate | C15H18O8 | 991999488 |
| Neg_00033 | Negative | 1.002333 | 173.0087 | Trans-Aconitic Acid | C6H6O6 | 1953729536 |
| Neg_00314 | Negative | 6.32575 | 163.0395 | Trans-4-Coumaric Acid | C9H8O3 | 8799563776 |
| Pos_00606 | Positive | 7.589467 | 331.1175 | Toddalolactone | C16H20O6 | 218785376 |
| Pos_00611 | Positive | 6.782017 | 409.147 | Tinnevellin Glucoside | C20H24O9 | 90746648 |
| Neg_00215 | Negative | 3.679533 | 99.04401 | Tiglic Acid | C5H8O2 | 231123088 |
| Neg_00037 | Negative | 4.99925 | 287.0902 | Thymidine | C10H14N2O5 | 127745944 |
| Pos_00328 | Positive | 7.659133 | 271.0599 | Thunberginol A | C15H10O5 | 10095760384 |
| Pos_00530 | Positive | 0.9635333 | 175.1076 | Theanine | C7H14N2O3 | 7712886272 |
| Pos_00043 | Positive | 8.0626 | 365.2321 | The | C21H32O5 | 179918704 |
| Pos_00634 | Positive | 9.888833 | 269.0802 | Tectochrysin | C16H12O4 | 192245392 |
| Pos_00224 | Positive | 5.555483 | 181.0495 | Syringic Acid | C9H10O5 | 717552256 |
| Neg_00239 | Negative | 6.0035 | 197.0457 | Syringic Acid | C9H10O5 | 2997122048 |
| Pos_00310 | Positive | 1.227683 | 365.1049 | Sucrose | C12H22O11 | 163837808 |
| Neg_00167 | Negative | 2.577517 | 99.00754 | Succinic Anhydride | C4H4O3 | 492535072 |
| Neg_00218 | Negative | 2.577517 | 117.0185 | Succinate | C4H6O4 | 11148505088 |
| Neg_00074 | Negative | 6.311717 | 173.0814 | Suberate | C8H14O4 | 688151040 |
| Pos_00414 | Positive | 7.172033 | 121.0651 | Styrene Oxide | C8H8O | 340092576 |
| Pos_00665 | Positive | 5.13845 | 105.0703 | Styrene | C8H8 | 427121312 |
| Pos_00169 | Positive | 4.888317 | 298.1394 | Stepharine | C18H19NO3 | 287239104 |
| Pos_00244 | Positive | 8.954833 | 259.2057 | Stearidonic Acid | C18H28O2 | 561911808 |
| Pos_00355 | Positive | 1.407817 | 684.2566 | Stachyose | C24H42O21 | 39543544 |
| Pos_00736 | Positive | 0.9635333 | 144.1018 | Stachydrine | C7H13NO2 | 18316599296 |
| Pos_00265 | Positive | 5.277433 | 237.1236 | Spectral Match To Phe-Ala From Nist14 | C12H16N2O3 | 233032896 |
| Pos_00158 | Positive | 2.766783 | 165.0545 | Spectral Match To L-Tyrosine From Nist14 [Iin-Based On | C9H11NO3 | 1125176576 |
| Pos_00217 | Positive | 1.158183 | 163.0603 | Spectral Match To D-(+)-Mannose From Nist14 | C6H12O6 | 376044448 |
| Pos_00226 | Positive | 8.006933 | 219.1742 | Spectral Match To Curcumol From Nist14 | C15H24O2 | 2452980992 |
| Pos_00182 | Positive | 11.2709 | 279.232 | Spectral Match To 9(10)-Epome From Nist14 | C18H32O3 | 50362176 |
| Pos_00263 | Positive | 9.9168 | 315.1228 | Spectral Match To 4',5,7-Trimethoxyflavanone From Nist14 | C18H18O5 | 72091192 |
| Pos_00181 | Positive | 7.36685 | 195.1744 | Spectral Match To 2-Butanone, 4-(2,6,6-Trimethyl-2-Cyclohexen-1-Yl)- From Nist14 | C13H22O | 197381696 |
| Pos_00240 | Positive | 10.2929 | 522.3552 | Spectral Match To 1-(9Z-Octadecenoyl)-Sn-Glycero-3-Phosphocholine From Nist14 | C26H52NO7P | 242411248 |
| Pos_00249 | Positive | 1.019183 | 198.0971 | Spectral Match To .Beta.-D-Glucose From Nist14 | C6H12O6 | 525051744 |
| Pos_00203 | Positive | 6.364316 | 135.1169 | Spectral Match To (-)-Carveol From Nist14 | C10H16O | 543918784 |
| Pos_00702 | Positive | 5.041183 | 260.0917 | Skimmianine | C14H13NO4 | 357448608 |
| Pos_00125 | Positive | 6.3086 | 193.086 | Sinapyl Alcohol | C11H14O4 | 234309936 |
| Neg_00282 | Negative | 4.985283 | 173.0453 | Shikimate | C7H10O5 | 489994752 |
| Neg_00150 | Negative | 1.312783 | 137.0237 | Sesamol | C7H6O3 | 350036000 |
| Neg_00163 | Negative | 0.9466833 | 209.0666 | Sedoheptulose | C7H14O7 | 357326976 |
| Neg_00078 | Negative | 7.264333 | 201.1131 | Sebacic Acid | C10H18O4 | 519254784 |
| Neg_00123 | Negative | 0.9187833 | 179.0556 | Scyllo-Inositol | C6H12O6 | 3846996736 |
| Pos_00102 | Positive | 6.949383 | 287.0552 | Scutellarein | C15H10O6 | 5686912512 |
| Neg_00002 | Negative | 5.7798 | 191.0347 | Scopoletin | C10H8O4 | 134464800 |
| Pos_00638 | Positive | 8.6485 | 439.3215 | Sarsasapogenin | C27H44O3 | 111009224 |
| Pos_00503 | Positive | 0.9078667 | 90.05552 | Sarcosine | C3H7NO2 | 494576576 |
| Pos_00576 | Positive | 5.666934 | 194.1176 | Salsoline | C11H15NO2 | 268959360 |
| Neg_00207 | Negative | 4.92965 | 194.0457 | Salicyluric Acid | C9H9NO4 | 232099680 |
| Pos_00510 | Positive | 5.666934 | 139.039 | Salicylate | C7H6O3 | 1560089600 |
| Neg_00004 | Negative | 1.002333 | 271.0585 | Rubrofusarin | C15H12O5 | 452498944 |
| Neg_00335 | Negative | 8.701417 | 253.0519 | Rubiadin | C15H10O4 | 851496384 |
| Pos_00156 | Positive | 6.6287 | 361.0915 | Rosmarinic Acid | C18H16O8 | 274014176 |
| Neg_00272 | Negative | 6.6481 | 359.0781 | Rosmarinic Acid | C18H16O8 | 25148596224 |
| Pos_00223 | Positive | 6.6287 | 343.0809 | Rosmarinate | C18H16O8 | 269731776 |
| Neg_00036 | Negative | 6.6481 | 395.0554 | Rosmarinate | C18H16O8 | 1000002880 |
| Pos_00228 | Positive | 5.72265 | 377.1453 | Riboflavin | C17H20N4O6 | 258436096 |
| Pos_00624 | Positive | 1.338583 | 259.0929 | Rhapontigenin | C15H14O4 | 754199872 |
| Pos_00707 | Positive | 8.25795 | 266.1753 | Reynosin | C15H20O3 | 100054608 |
| Pos_00513 | Positive | 1.477333 | 522.2028 | Raffinose | C18H32O16 | 155206112 |
| Neg_00091 | Negative | 1.4796 | 539.1418 | Raffinose | C18H32O16 | 71962656 |
| Pos_00721 | Positive | 5.918 | 479.0815 | Quercetin 3-O-Glucuronide | C21H18O13 | 120002048 |
| Pos_00057 | Positive | 6.001817 | 465.1021 | Quercetin 3-O-Glucoside | C21H20O12 | 154856496 |
| Pos_00256 | Positive | 6.001817 | 303.0503 | Quercetin | C15H10O7 | 324252576 |
| Neg_00003 | Negative | 7.29235 | 301.0367 | Quercetin | C15H10O7 | 183054240 |
| Pos_00487 | Positive | 4.527817 | 86.06075 | Pyrrolidin-2-One | C4H7NO | 10082402304 |
| Pos_00127 | Positive | 5.750517 | 112.0397 | Pyrrole-2-Carboxylate | C5H5NO2 | 1678090624 |
| Neg_00031 | Negative | 2.911133 | 110.0238 | Pyrrole-2-Carboxylate | C5H5NO2 | 887373056 |
| Pos_00584 | Positive | 2.253717 | 170.0813 | Pyridoxine | C8H11NO3 | 1354794368 |
| Pos_00094 | Positive | 1.934883 | 168.0655 | Pyridoxal | C8H9NO3 | 2971545600 |
| Neg_00291 | Negative | 4.957467 | 166.0507 | Pyridoxal | C8H9NO3 | 108845104 |
| Pos_00309 | Positive | 0.8800833 | 203.0523 | Psicose | C6H12O6 | 203890656 |
| Pos_00173 | Positive | 8.8016 | 413.1584 | Pseudolaric Acid C | C21H26O7 | 49932836 |
| Pos_00461 | Positive | 1.060833 | 97.02885 | Protoanemonin | C5H4O2 | 353707648 |
| Pos_00307 | Positive | 5.9739 | 181.0859 | Propylparaben | C10H12O3 | 1508601600 |
| Pos_00001 | Positive | 1.616 | 260.1603 | Propranolol | C16H21NO2 | 259838464 |
| Pos_00288 | Positive | 8.0905 | 210.1125 | Propoxur | C11H15NO3 | 184608544 |
| Pos_00514 | Positive | 0.9496334 | 116.071 | Proline | C5H9NO2 | 12134955008 |
| Pos_00031 | Positive | 6.1412 | 209.1173 | Primin | C12H16O3 | 6676360192 |
| Pos_00468 | Positive | 6.74025 | 443.1457 | Pomiferin | C25H24O6 | 54032380 |
| Pos_00273 | Positive | 2.766783 | 136.0758 | P-Octopamine | C8H11NO2 | 1445739904 |
| Pos_00410 | Positive | 7.478117 | 135.1169 | P-Mentha-1,3,8-Triene | C10H14 | 1074151552 |
| Pos_00435 | Positive | 7.533733 | 133.1013 | P-Mentha-1,3,5,8-Tetraene | C10H12 | 2348416768 |
| Pos_00728 | Positive | 7.227883 | 340.1183 | Piplartine | C17H19NO5 | 86362360 |
| Pos_00177 | Positive | 9.8052 | 361.1989 | Piperonyl Butoxide | C19H30O5 | 35323776 |
| Pos_00690 | Positive | 2.364717 | 86.09712 | Piperidine | C5H11N | 7996187136 |
| Pos_00342 | Positive | 0.9774 | 130.0864 | Pipecolate | C6H11NO2 | 20746178560 |
| Pos_00368 | Positive | 8.745983 | 257.0811 | Pinocembrine | C15H12O4 | 1208772096 |
| Neg_00087 | Negative | 8.770967 | 255.0674 | Pinocembrin | C15H12O4 | 2168401408 |
| Pos_00378 | Positive | 7.6452 | 195.1382 | Pinocarvyl Acetate | C12H18O2 | 721518144 |
| Neg_00194 | Negative | 6.171717 | 159.0658 | Pimelate | C7H12O4 | 1217221120 |
| Pos_00134 | Positive | 8.49485 | 318.3 | Phytosphingosine | C18H39NO3 | 196995952 |
| Pos_00301 | Positive | 8.745983 | 285.076 | Physcion | C16H12O5 | 1004209856 |
| Pos_00237 | Positive | 0.72675 | 149.0226 | Phthalic Anhydride | C8H4O3 | 849717824 |
| Pos_00569 | Positive | 5.638983 | 167.0338 | Phthalate | C8H6O4 | 129055792 |
| Neg_00235 | Negative | 5.863767 | 165.019 | Phthalate | C8H6O4 | 1809425536 |
| Neg_00260 | Negative | 13.75915 | 163.0395 | Phenylpyruvate | C9H8O3 | 625449664 |
| Pos_00356 | Positive | 4.971717 | 120.0809 | Phenylethanolamine | C8H11NO | 16420785152 |
| Pos_00082 | Positive | 4.971717 | 166.0862 | Phenylalanine | C9H11NO2 | 19597283328 |
| Pos_00056 | Positive | 2.766783 | 95.04975 | Phenol | C6H6O | 394559040 |
| Pos_00295 | Positive | 13.41232 | 122.0967 | Phenethylamine | C8H11N | 2360117760 |
| Pos_00567 | Positive | 6.1134 | 165.0912 | Phenethyl Acetate | C10H12O2 | 9920114688 |
| Pos_00403 | Positive | 6.280867 | 265.0973 | Perlolyrine | C16H12N2O2 | 700523776 |
| Neg_00168 | Negative | 7.362067 | 327.1469 | Perilloside B | C16H24O7 | 106799608 |
| Pos_00757 | Positive | 6.364316 | 314.1382 | Perfamine | C18H19NO4 | 95602184 |
| Neg_00161 | Negative | 1.271 | 75.00529 | Peracetic Acid | C2H4O3 | 286875968 |
| Pos_00575 | Positive | 5.444217 | 102.0918 | Pentanamide | C5H11NO | 765024128 |
| Pos_00382 | Positive | 11.68498 | 782.5685 | Pc(16:0/20:4(8Z,11Z,14Z,17Z)) | C44H80NO8P | 16640148 |
| Pos_00351 | Positive | 11.2709 | 808.5837 | Pc(16 | C46H82NO8P | 91403240 |
| Pos_00498 | Positive | 5.1246 | 220.1183 | Pantothenate | C9H17NO5 | 1674870784 |
| Pos_00650 | Positive | 5.24965 | 283.1649 | Panaxydiol | C17H24O2 | 133347760 |
| Pos_00696 | Positive | 6.6427 | 167.07 | Paeonol | C9H10O3 | 531428512 |
| Pos_00659 | Positive | 5.3052 | 263.1755 | Oxysophocarpine | C15H22N2O2 | 1624744704 |
| Neg_00336 | Negative | 6.802633 | 431.158 | Osmanthuside H | C19H28O11 | 69048256 |
| Pos_00457 | Positive | 5.583367 | 208.1333 | O-Methylcorypalline | C12H17NO2 | 857101376 |
| Pos_00154 | Positive | 4.971717 | 107.0496 | O-Methoxyphenol | C7H8O2 | 201996032 |
| Pos_00234 | Positive | 11.68498 | 439.3565 | Oleanolic Acid | C30H48O3 | 46814432 |
| Pos_00617 | Positive | 7.478117 | 195.1382 | Octyl Acetate | C10H20O2 | 1098879488 |
| Pos_00232 | Positive | 6.057533 | 136.0758 | Octopamine | C8H11NO2 | 519733888 |
| Pos_00248 | Positive | 12.22195 | 284.2946 | Octadecanamide | C18H37NO | 170063360 |
| Neg_00346 | Negative | 1.409983 | 146.0453 | O-Acetylserine | C5H9NO4 | 142054688 |
| Pos_00472 | Positive | 5.013417 | 220.1083 | N-Phenyl-2-Naphthylamine | C16H13N | 86767360 |
| Pos_00600 | Positive | 2.475667 | 241.1548 | Nootkatone | C15H22O | 598353664 |
| Pos_00465 | Positive | 8.7738 | 403.1382 | Nobiletin | C21H22O8 | 78441312 |
| Pos_00738 | Positive | 5.750517 | 138.0549 | N-Methylnicotinate | C7H7NO2 | 1210780288 |
| Pos_00492 | Positive | 8.7738 | 180.1384 | N-Methylephedrine | C11H17NO | 61005904 |
| Pos_00299 | Positive | 5.041183 | 100.0763 | N-Methyl-2-Pyrrolidinone | C5H9NO | 2934824192 |
| Pos_00144 | Positive | 1.657617 | 124.0395 | Nicotinate | C6H5NO2 | 23422511104 |
| Neg_00089 | Negative | 1.701633 | 122.0237 | Nicotinate | C6H5NO2 | 824333248 |
| Pos_00145 | Positive | 1.893333 | 123.0554 | Nicotinamide | C6H6N2O | 18304612352 |
| Pos_00467 | Positive | 7.1163 | 314.1382 | N-Feruloyltyramine | C18H19NO4 | 429568288 |
| Pos_00644 | Positive | 5.472034 | 245.1859 | Nerolidol | C15H26O | 146789760 |
| Pos_00664 | Positive | 1.53285 | 189.1232 | N-Epsilon-Acetyllysine | C8H16N2O3 | 418805600 |
| Neg_00060 | Negative | 5.27775 | 353.0892 | Neochlorogenic Acid | C16H18O9 | 103812256 |
| Pos_00072 | Positive | 3.155167 | 194.0812 | N-Benzoyl-Dl-Alanine | C10H11NO3 | 390394304 |
| Pos_00557 | Positive | 7.631267 | 275.2002 | Nandrolone | C18H26O2 | 2898488576 |
| Pos_00481 | Positive | 1.616 | 190.071 | N-Acetyl-Dl-Glutamic Acid | C7H11NO5 | 3700889088 |
| Neg_00189 | Negative | 1.61835 | 188.0567 | N-Acetyl-Dl-Glutamic Acid | C7H11NO5 | 9863733248 |
| Pos_00221 | Positive | 1.074767 | 259.1285 | N6-(L-1,3-Dicarboxypropyl)-L-Lysine | C11H20N2O6 | 955032704 |
| Pos_00653 | Positive | 5.346933 | 197.0921 | N5-Ethyl-L-Glutamine | C7H14N2O3 | 438031200 |
| Pos_00574 | Positive | 5.49975 | 266.1383 | N(Alpha)-Benzyloxycarbonyl-L-Leucine | C14H19NO4 | 359541536 |
| Neg_00169 | Negative | 8.19965 | 503.3402 | Myrianthic Acid | C30H48O6 | 96996904 |
| Pos_00744 | Positive | 8.006933 | 159.117 | Myrcene | C10H16 | 201165712 |
| Pos_00302 | Positive | 4.070384 | 88.07635 | Morpholine | C4H9NO | 400384672 |
| Pos_00432 | Positive | 6.46175 | 121.1015 | M-Ethyl_Toluene | C9H12 | 352483872 |
| Pos_00554 | Positive | 6.977217 | 225.1483 | Methyl Jasmonate | C13H20O3 | 1083208192 |
| Neg_00035 | Negative | 7.04075 | 193.0504 | Methyl Caffeate | C10H10O4 | 519250144 |
| Pos_00762 | Positive | 7.770333 | 235.0599 | Methyl 3,5-Dimethoxy-4-Hydroxy-Benzoate | C10H12O5 | 328139712 |
| Neg_00146 | Negative | 3.412567 | 125.0236 | Methyl 2-Furoate | C6H6O3 | 1091057280 |
| Pos_00739 | Positive | 1.657617 | 166.0501 | Methionine Sulfoxide | C5H11NO3S | 523974688 |
| Neg_00191 | Negative | 0.9187833 | 149.0083 | Meso-Tartaric Acid | C4H6O6 | 3277157888 |
| Neg_00286 | Negative | 3.777317 | 129.0184 | Mesaconate | C5H6O4 | 1203299840 |
| Pos_00450 | Positive | 4.874434 | 180.1018 | Maltoxazine | C10H13NO2 | 450908128 |
| Neg_00095 | Negative | 0.9187833 | 377.0869 | Maltose | C12H22O11 | 331089440 |
| Pos_00572 | Positive | 5.4303 | 127.0392 | Maltol | C6H6O3 | 3304044544 |
| Neg_00124 | Negative | 5.291667 | 87.00521 | Malonic Semialdehyde | C3H4O3 | 286684992 |
| Neg_00081 | Negative | 1.002333 | 103.0025 | Malonic Acid | C3H4O4 | 7673048064 |
| Neg_00225 | Negative | 0.9606333 | 115.0026 | Maleic Acid | C4H4O4 | 2153535232 |
| Pos_00651 | Positive | 0.9635333 | 337.1597 | Magnocurarine | C19H24NO3+ | 1071966400 |
| Pos_00104 | Positive | 8.188184 | 487.343 | Madecassic Acid | C30H48O6 | 113973512 |
| Neg_00304 | Negative | 8.5762 | 503.3405 | Madecassic Acid | C30H48O6 | 115765184 |
| Pos_00362 | Positive | 1.407817 | 118.0866 | L-Valine | C5H11NO2 | 12649570304 |
| Neg_00352 | Negative | 5.975567 | 447.0958 | Luteolin 7-O-Beta-D-Glucoside | C21H20O11 | 246654944 |
| Pos_00527 | Positive | 7.241783 | 287.0554 | Luteolin | C15H10O6 | 13241806848 |
| Neg_00343 | Negative | 7.250417 | 285.0413 | Luteolin | C15H10O6 | 10353655808 |
| Pos_00258 | Positive | 0.9635333 | 182.0811 | L-Tyrosine | C9H11NO3 | 1860358144 |
| Pos_00577 | Positive | 5.3052 | 205.0974 | L-Tryptophan | C11H12N2O2 | 3624315136 |
| Pos_00479 | Positive | 2.503383 | 116.071 | L-Proline | C5H9NO2 | 576091776 |
| Pos_00579 | Positive | 1.005183 | 262.1287 | Lotaustralin | C11H19NO6 | 1333856640 |
| Pos_00167 | Positive | 6.823983 | 179.1069 | Loliolide [Iin-Based On | C11H16O3 | 3844686336 |
| Pos_00162 | Positive | 6.823983 | 197.1169 | Loliolide [Iin-Based | C11H16O3 | 32821983232 |
| Pos_00592 | Positive | 13.70245 | 197.1169 | Loliolide | C11H16O3 | 69110440 |
| Neg_00187 | Negative | 5.222034 | 116.0706 | L-Norvaline | C5H11NO2 | 152961456 |
| Neg_00283 | Negative | 3.973383 | 130.0865 | L-Leucine | C6H13NO2 | 444018496 |
| Neg_00245 | Negative | 2.66085 | 130.0865 | L-Isoleucine | C6H13NO2 | 162267856 |
| Pos_00599 | Positive | 4.9856 | 219.1336 | Linalyl Acetate | C12H20O2 | 245482976 |
| Pos_00552 | Positive | 7.380733 | 155.143 | Linalool | C10H18O | 178077376 |
| Pos_00759 | Positive | 9.317767 | 493.1863 | Limonin | C26H30O8 | 40737884 |
| Pos_00262 | Positive | 6.6009 | 153.1273 | Limonene-1,2-Epoxide | C10H16O | 384647232 |
| Pos_00323 | Positive | 6.1412 | 191.1067 | Ligustilide | C12H14O2 | 2235457792 |
| Pos_00626 | Positive | 6.2389 | 327.1553 | Licarin A | C20H22O4 | 619931264 |
| Pos_00357 | Positive | 4.860517 | 102.0554 | L-Homoserine | C4H9NO3 | 1622269568 |
| Neg_00219 | Negative | 0.9606333 | 177.0399 | L-Gulono-1,4-Lactone | C6H10O6 | 354305248 |
| Neg_00011 | Negative | 1.521233 | 177.0399 | L-Gulcono-1,4-Lactone | C6H10O6 | 2190817280 |
| Neg_00257 | Negative | 0.8908167 | 146.0453 | L-Glutamate | C5H9NO4 | 167612960 |
| Neg_00259 | Negative | 7.641733 | 387.1462 | Levocetirizine | C21H25ClN2O3 | 965825216 |
| Pos_00138 | Positive | 9.526617 | 247.1328 | Leukamesine, Desacetoxymatricarin | C15H18O3 | 183030320 |
| Pos_00681 | Positive | 4.375283 | 132.102 | Leucine; Lc-Esi-Qtof | C6H13NO2 | 3202763008 |
| Pos_00040 | Positive | 5.0273 | 130.0864 | L-Beta-Homoproline | C6H11NO2 | 3525627648 |
| Neg_00232 | Negative | 0.8193333 | 132.0293 | L-Aspartate | C4H7NO4 | 51932820 |
| Pos_00523 | Positive | 0.8800833 | 175.1191 | L-Arginine | C6H14N4O2 | 647186240 |
| Pos_00490 | Positive | 2.184433 | 162.0763 | L-2-Aminoadipate | C6H11NO4 | 364643552 |
| Neg_00292 | Negative | 1.54905 | 160.0609 | L-2-Aminoadipate | C6H11NO4 | 297287808 |
| Pos_00632 | Positive | 10.08387 | 439.2124 | Kurarinone | C26H30O6 | 37147580 |
| Pos_00053 | Positive | 5.416467 | 143.0339 | Kojic Acid | C6H6O4 | 4382756864 |
| Pos_00729 | Positive | 7.35295 | 261.1231 | Kobusone | C14H22O2 | 185458256 |
| Pos_00727 | Positive | 5.13845 | 216.0879 | Kinetin | C10H9N5O | 473275040 |
| Pos_00754 | Positive | 7.199917 | 283.0596 | Khellin | C14H12O5 | 128247336 |
| Pos_00594 | Positive | 5.736483 | 214.1077 | Kainic Acid | C10H15NO4 | 322388832 |
| Neg_00305 | Negative | 6.339716 | 447.0961 | Kaempferol-7-O-Glucoside | C21H20O11 | 96766136 |
| Pos_00246 | Positive | 5.959933 | 449.1073 | Kaempferol 3-O-Glucoside | C21H20O11 | 1508446592 |
| Pos_00720 | Positive | 6.1971 | 463.0872 | Kaempferol 3-Glucuronide | C21H18O12 | 6659613184 |
| Neg_00344 | Negative | 6.2279 | 461.0739 | Kaempferol 3-Glucuronide | C21H18O12 | 1494797696 |
| Pos_00006 | Positive | 5.959933 | 287.0551 | Kaempferol | C15H10O6 | 2360512512 |
| Neg_00297 | Negative | 6.956683 | 285.0422 | Kaempferol | C15H10O6 | 1094576768 |
| Neg_00295 | Negative | 7.404083 | 299.0575 | Kaempferide | C16H12O6 | 154296304 |
| Pos_00526 | Positive | 6.127267 | 433.1128 | Isovitexin | C21H20O10 | 244429632 |
| Neg_00160 | Negative | 5.152517 | 167.0344 | Isovanillic Acid | C8H8O4 | 2875503872 |
| Pos_00694 | Positive | 10.06985 | 408.2748 | Isotalatizidine | C23H37NO5 | 30635448 |
| Neg_00018 | Negative | 9.0914 | 315.0522 | Isorhamnetin | C16H12O7 | 90555392 |
| Pos_00035 | Positive | 7.311316 | 139.1118 | Isophorone | C9H14O | 223954192 |
| Pos_00469 | Positive | 8.383317 | 384.2737 | Isomuronic Acid | C21H34O5 | 58168016 |
| Pos_00341 | Positive | 2.642 | 132.102 | Isoleucine | C6H13NO2 | 51212660736 |
| Pos_00518 | Positive | 8.244 | 223.1691 | Isokobusone | C14H22O2 | 310972352 |
| Neg_00334 | Negative | 8.603933 | 269.083 | Isoimperatorin | C16H14O4 | 208864032 |
| Neg_00306 | Negative | 7.3342 | 193.0509 | Isoferulic Acid | C10H10O4 | 264839616 |
| Pos_00612 | Positive | 6.837917 | 257.1497 | Isocurcumenol | C15H22O2 | 157167968 |
| Neg_00186 | Negative | 0.9606333 | 191.0197 | Isocitrate | C6H8O8 | 3038304512 |
| Pos_00101 | Positive | 8.745983 | 233.1531 | Isoalantolactone | C15H20O2 | 402260672 |
| Neg_00206 | Negative | 0.93275 | 267.0733 | Inosine | C10H12N4O5 | 539118528 |
| Pos_00202 | Positive | 5.6251 | 144.0809 | Indole-3-Ethanol | C10H11NO | 1299376256 |
| Pos_00045 | Positive | 6.879783 | 146.0602 | Indole-3-Carboxaldehyde | C9H7NO | 3089411072 |
| Neg_00240 | Negative | 6.900617 | 144.0448 | Indole-3-Carboxaldehyde | C9H7NO | 655003456 |
| Pos_00036 | Positive | 6.503467 | 176.0704 | Indole-3-Acetate | C10H9NO2 | 705220864 |
| Neg_00049 | Negative | 6.52195 | 174.0558 | Indole-3-Acetate | C10H9NO2 | 378752576 |
| Pos_00110 | Positive | 7.06055 | 160.0756 | Indole-3-Acetaldehyde | C10H9NO | 144501920 |
| Pos_00470 | Positive | 6.1412 | 118.0653 | Indole | C8H7N | 708387264 |
| Neg_00216 | Negative | 6.395667 | 116.0496 | Indole | C8H7N | 311586432 |
| Pos_00480 | Positive | 5.096867 | 134.0602 | Indican | C8H7NO | 351848576 |
| Pos_00188 | Positive | 7.3391 | 291.2678 | Icosadienoic Acid | C20H36O2 | 142952144 |
| Pos_00345 | Positive | 2.128967 | 137.0457 | Hypoxanthine | C5H4N4O | 3437950464 |
| Neg_00347 | Negative | 0.8908167 | 135.0288 | Hypoxanthine | C5H4N4O | 4326419968 |
| Pos_00524 | Positive | 4.818867 | 111.0444 | Hydroquinone | C6H6O2 | 236071712 |
| Pos_00742 | Positive | 5.583367 | 166.1227 | Hordenine | C10H15NO | 264932784 |
| Pos_00589 | Positive | 1.491167 | 112.0873 | Histamine | C5H9N3 | 251389424 |
| Pos_00084 | Positive | 3.4742 | 180.0657 | Hippurate | C9H9NO3 | 280346592 |
| Neg_00017 | Negative | 5.305517 | 178.0508 | Hippurate | C9H9NO3 | 111086544 |
| Pos_00767 | Positive | 6.74025 | 309.2023 | Hexadecanedioate | C16H30O4 | 96390664 |
| Pos_00211 | Positive | 11.55513 | 256.2629 | Hexadecanamide | C16H33NO | 77058464 |
| Pos_00464 | Positive | 8.132383 | 473.3253 | Hecogenin Acetate | C29H44O5 | 195246112 |
| Pos_00453 | Positive | 5.694767 | 199.0865 | Harmanine | C12H10N2O | 141356832 |
| Pos_00176 | Positive | 4.83275 | 284.0983 | Guanosine | C10H13N5O5 | 1682378368 |
| Neg_00233 | Negative | 4.83225 | 282.086 | Guanosine | C10H13N5O5 | 185633168 |
| Pos_00214 | Positive | 1.53285 | 152.0565 | Guanine | C5H5N5O | 7340396032 |
| Neg_00258 | Negative | 1.53515 | 150.0415 | Guanine | C5H5N5O | 111808600 |
| Neg_00345 | Negative | 6.05975 | 433.079 | Guajavarin | C20H18O11 | 213359664 |
| Neg_00103 | Negative | 8.96605 | 223.1708 | Goshuyic Acid | C14H24O2 | 206516464 |
| Neg_00293 | Negative | 3.665633 | 362.0522 | Gmp | C10H14N5O8P | 93543872 |
| Neg_00107 | Negative | 7.0826 | 297.0416 | Glyzaglabrin | C16H10O6 | 112443304 |
| Pos_00140 | Positive | 9.652034 | 453.335 | Glycyrrhetinate | C30H46O4 | 86380576 |
| Pos_00220 | Positive | 7.144134 | 447.128 | Glycitin | C22H22O10 | 119790552 |
| Neg_00165 | Negative | 0.8908167 | 245.0449 | Glycerophosphoglycerol | C6H15O8P | 148128768 |
| Neg_00092 | Negative | 1.1997 | 89.02325 | Glyceraldehyde | C3H6O3 | 6103685632 |
| Neg_00226 | Negative | 5.096717 | 131.0342 | Glutarate | C5H8O4 | 1076760832 |
| Pos_00275 | Positive | 0.8939833 | 148.0605 | Glutamate | C5H9NO4 | 235170944 |
| Pos_00610 | Positive | 8.732067 | 455.3146 | Gitogenin | C27H44O4 | 58596260 |
| Pos_00692 | Positive | 8.634533 | 295.19 | Gingerol | C17H26O4 | 974195456 |
| Pos_00750 | Positive | 7.478117 | 177.1273 | Geraniol | C10H18O | 188751216 |
| Pos_00551 | Positive | 6.6148 | 169.1223 | Geranic Acid | C10H16O2 | 165944608 |
| Neg_00013 | Negative | 5.096717 | 401.1101 | Gentiopicroside | C16H20O9 | 107922352 |
| Pos_00449 | Positive | 5.890033 | 166.0861 | Gentiatibetine | C9H11NO2 | 923341888 |
| Pos_00607 | Positive | 5.0273 | 176.0705 | Gentianine | C10H9NO2 | 255443984 |
| Neg_00303 | Negative | 8.770967 | 283.0626 | Genkwanin | C16H12O5 | 272453056 |
| Pos_00186 | Positive | 6.224933 | 433.1125 | Genistein 7-O-Beta-D-Glucoside | C21H20O10 | 286400704 |
| Pos_00011 | Positive | 13.67473 | 271.0598 | Genistein | C15H10O5 | 288149856 |
| Neg_00279 | Negative | 5.4031 | 387.1317 | Geniposide | C17H24O10 | 95223456 |
| Neg_00311 | Negative | 5.961633 | 321.1565 | Gelsemine | C20H22N2O2 | 87995336 |
| Pos_00647 | Positive | 5.54155 | 309.0969 | Gastrodin | C13H18O7 | 251625072 |
| Neg_00172 | Negative | 8.743083 | 517.318 | Ganolucidic Acid C | C30H46O7 | 534251680 |
| Neg_00105 | Negative | 9.2728 | 499.308 | Ganolucidic Acid A | C30H44O6 | 251827920 |
| Pos_00615 | Positive | 11.96212 | 593.2745 | Ganoderic Acid F | C32H42O9 | 142425200 |
| Neg_00106 | Negative | 8.422633 | 515.3041 | Ganoderic Acid Delta | C30H44O7 | 86448296 |
| Neg_00113 | Negative | 9.412066 | 499.3081 | Ganoderic Acid Beta | C30H44O6 | 73256280 |
| Neg_00012 | Negative | 5.013117 | 331.0684 | Gallic Acid Hexoside | C13H16O10 | 226851328 |
| Pos_00528 | Positive | 5.583367 | 171.0289 | Gallate | C7H6O5 | 142915328 |
| Neg_00267 | Negative | 4.169816 | 169.0138 | Gallate | C7H6O5 | 1089069824 |
| Neg_00077 | Negative | 1.61835 | 115.0026 | Fumaric Acid | C4H4O4 | 3658713600 |
| Neg_00349 | Negative | 1.312783 | 115.0026 | Fumarate | C4H4O4 | 1394898560 |
| Neg_00006 | Negative | 5.249933 | 307.0477 | Fulvic Acid | C14H12O8 | 79341592 |
| Pos_00602 | Positive | 6.2389 | 223.06 | Fraxidin | C11H10O5 | 881716288 |
| Neg_00019 | Negative | 5.612167 | 207.0302 | Fraxetin | C10H8O5 | 429863008 |
| Neg_00001 | Negative | 7.404083 | 440.1372 | Folate | C19H19N7O6 | 223065312 |
| Pos_00550 | Positive | 5.6251 | 167.0818 | Fluorene | C13H10 | 356281888 |
| Neg_00166 | Negative | 7.431983 | 307.0734 | Flazine | C17H12N2O4 | 160315968 |
| Pos_00282 | Positive | 5.987884 | 177.0546 | Ferulate | C10H10O4 | 481822208 |
| Neg_00299 | Negative | 9.17505 | 299.0941 | Farrerol | C17H16O5 | 199873904 |
| Neg_00014 | Negative | 10.12115 | 295.2293 | Fa 18 | C18H32O3 | 257169792 |
| Neg_00300 | Negative | 5.905633 | 343.0839 | Eupatilin | C18H16O7 | 147257088 |
| Pos_00508 | Positive | 5.485867 | 141.0548 | Ethyl Maltol | C7H8O3 | 245296112 |
| Pos_00068 | Positive | 5.360867 | 158.0812 | Et Ester-(R)-5-Oxo-2-Pyrrolidinecarboxylic Acid | C7H11NO3 | 697427200 |
| Pos_00471 | Positive | 8.815583 | 271.1689 | Estrone | C18H22O2 | 84939728 |
| Pos_00764 | Positive | 8.815583 | 289.1797 | Estriol | C18H24O3 | 550331328 |
| Pos_00062 | Positive | 7.993117 | 273.1846 | Estradiol-17Beta | C18H24O2 | 326225216 |
| Pos_00751 | Positive | 5.876067 | 179.0342 | Esculetin | C9H6O4 | 1102121728 |
| Neg_00273 | Negative | 5.8777 | 177.0193 | Esculetin | C9H6O4 | 3663231232 |
| Neg_00127 | Negative | 9.42595 | 501.3248 | Esculentic Acid | C30H46O6 | 257080400 |
| Pos_00150 | Positive | 6.419967 | 159.0804 | Epiestradiol | C18H24O2 | 171947840 |
| Pos_00287 | Positive | 4.9856 | 181.097 | Dulcin | C9H12N2O2 | 216035632 |
| Neg_00262 | Negative | 4.40865 | 321.0515 | Dtmp | C10H15N2O8P | 115423448 |
| Pos_00477 | Positive | 10.41842 | 322.2741 | D-Sphingosine | C18H37NO2 | 123895064 |
| Neg_00205 | Negative | 1.0858 | 131.0341 | D-Ribose | C5H10O5 | 220762752 |
| Pos_00396 | Positive | 1.50505 | 130.0864 | D-Pipecolic Acid | C6H11NO2 | 23522920448 |
| Neg_00034 | Negative | 5.138583 | 218.1037 | D-Pantothenic Acid | C9H17NO5 | 280462592 |
| Pos_00683 | Positive | 5.4581 | 154.0863 | Dopamine | C8H11NO2 | 387834176 |
| Pos_00359 | Positive | 0.9078667 | 180.0864 | D-Mannosamine | C6H13NO5 | 591066112 |
| Neg_00289 | Negative | 3.342617 | 103.039 | Dl-Beta-Hydroxybutyric Acid | C4H8O3 | 522074880 |
| Pos_00719 | Positive | 7.79825 | 301.0709 | Diosmetin | C16H12O6 | 1285459200 |
| Pos_00250 | Positive | 13.00502 | 391.2845 | Di-N-Octyl Phthalate | C24H38O4 | 36998364 |
| Pos_00544 | Positive | 1.019183 | 79.02194 | Dimethyl Sulfoxide | C2H6OS | 3562475520 |
| Pos_00318 | Positive | 8.508817 | 181.1224 | Dihydroactinidiolide | C11H16O2 | 6636996096 |
| Neg_00044 | Negative | 8.338883 | 221.0827 | Diethyl Phthalate | C12H14O4 | 121424568 |
| Pos_00051 | Positive | 10.64148 | 279.1591 | Dibutyl Phthalate | C16H22O4 | 828716352 |
| Pos_00484 | Positive | 0.9078667 | 87.0447 | Diacetyl | C4H6O2 | 696339712 |
| Neg_00022 | Negative | 5.3474 | 145.0499 | Diacetyl | C4H6O2 | 224397200 |
| Neg_00248 | Negative | 0.9466833 | 105.0181 | D-Glyceric Acid | C3H6O4 | 682727040 |
| Neg_00265 | Negative | 13.75915 | 193.0351 | D-Glucuronic Acid | C6H10O7 | 301445984 |
| Neg_00048 | Negative | 13.7314 | 179.0561 | D-Glucose | C6H12O6 | 2285338368 |
| Pos_00360 | Positive | 1.033133 | 180.0863 | D-Glucosamine | C6H13NO5 | 813406592 |
| Neg_00090 | Negative | 0.90475 | 195.0508 | D-Gluconic Acid | C6H12O7 | 9756442624 |
| Pos_00271 | Positive | 12.54293 | 357.2786 | Deoxycholic Acid | C24H40O4 | 18593566 |
| Pos_00350 | Positive | 1.53285 | 146.1176 | Deoxycarnitine | C7H15NO2 | 1191568384 |
| Pos_00225 | Positive | 12.7178 | 371.1014 | Decamethylcyclopentasiloxane | C10H30O5Si5 | 13644154 |
| Pos_00697 | Positive | 0.9496334 | 162.0758 | D-2-Aminoadipic Acid | C6H11NO4 | 753706240 |
| Neg_00324 | Negative | 0.9606333 | 133.0132 | D-(+)-Malic Acid | C4H6O5 | 30011942912 |
| Neg_00009 | Negative | 0.90475 | 193.0351 | D-(+)-Galacturonic Acid | C6H10O7 | 703755264 |
| Pos_00699 | Positive | 5.680867 | 309.0968 | D-(-)-Salicin | C13H18O7 | 346721792 |
| Pos_00183 | Positive | 1.53285 | 112.0507 | Cytosine | C4H5N3O | 2923786496 |
| Pos_00705 | Positive | 5.890033 | 213.1023 | Cytisine | C11H14N2O | 172808992 |
| Pos_00175 | Positive | 1.53285 | 244.0925 | Cytidine | C9H13N3O5 | 1501584640 |
| Neg_00288 | Negative | 1.53515 | 242.0788 | Cytidine | C9H13N3O5 | 35714088 |
| Pos_00325 | Positive | 8.006933 | 237.1851 | Curcumol | C15H24O2 | 1510160640 |
| Pos_00725 | Positive | 7.686867 | 235.1694 | Curcumenol | C15H22O2 | 1040820928 |
| Pos_00726 | Positive | 4.999533 | 297.1446 | Cryptotanshinone | C19H20O3 | 211879120 |
| Pos_00291 | Positive | 5.082867 | 233.1497 | Costunolide | C15H20O2 | 271948704 |
| Pos_00656 | Positive | 1.088667 | 274.0917 | Cordycepin | C10H13N5O3 | 5591558144 |
| Pos_00619 | Positive | 6.6287 | 203.0702 | Coniferyl Alcohol | C10H12O3 | 106026888 |
| Pos_00715 | Positive | 8.592533 | 411.2373 | Coleonol | C22H34O7 | 88552448 |
| Pos_00241 | Positive | 6.92155 | 400.1758 | Colchicine | C22H25NO6 | 925225216 |
| Pos_00675 | Positive | 8.508817 | 425.1842 | Clindamycin | C18H33ClN2O5S | 74248216 |
| Neg_00329 | Negative | 4.647867 | 154.0138 | Citrazinic Acid | C6H5NO4 | 450378912 |
| Pos_00753 | Positive | 1.185967 | 215.0163 | Citrate | C6H8O7 | 1638192128 |
| Neg_00179 | Negative | 1.142217 | 191.0197 | Citrate | C6H8O7 | 1.608E+11 |
| Neg_00294 | Negative | 1.185783 | 129.0184 | Citraconic Acid | C5H6O4 | 1932387456 |
| Pos_00448 | Positive | 13.67473 | 165.0545 | Cis-P-Coumaric Acid | C9H8O3 | 125019320 |
| Neg_00274 | Negative | 2.63305 | 173.0086 | Cis-Aconitate | C6H6O6 | 1802113536 |
| Neg_00296 | Negative | 7.809 | 299.0576 | Chrysoeriol | C16H12O6 | 222796160 |
| Pos_00717 | Positive | 8.676333 | 255.0648 | Chrysin | C15H10O4 | 1828113792 |
| Neg_00281 | Negative | 5.5425 | 465.1047 | Chrysanthemin | C21H21O11 | 95951344 |
| Pos_00038 | Positive | 0.8800833 | 104.1074 | Choline | C5H14NO | 48282730496 |
| Pos_00700 | Positive | 9.150167 | 426.3217 | Cholic Acid | C24H40O5 | 71106296 |
| Pos_00695 | Positive | 5.096867 | 209.0922 | Chalcone | C15H12O | 570134400 |
| Neg_00038 | Negative | 5.249933 | 109.0285 | Catechol | C6H6O2 | 2717089792 |
| Pos_00073 | Positive | 0.9218333 | 162.1125 | Carnitine | C7H15NO3 | 1461688320 |
| Pos_00013 | Positive | 5.416467 | 202.0863 | Carbaryl | C12H11NO2 | 356846976 |
| Pos_00541 | Positive | 9.568483 | 306.2061 | Capsaicin | C18H27NO3 | 119583064 |
| Pos_00393 | Positive | 1.56055 | 176.0915 | Calystegine B2 | C7H13NO4 | 443176192 |
| Pos_00087 | Positive | 6.6287 | 163.039 | Caffeicacid | C9H8O4 | 6128434176 |
| Neg_00280 | Negative | 7.096717 | 135.0441 | Caffeic Acid | C9H8O4 | 13745989632 |
| Pos_00095 | Positive | 6.350433 | 163.039 | Caffeate | C9H8O4 | 520009280 |
| Pos_00152 | Positive | 7.770333 | 263.1642 | Brefeldin A, Natural | C16H24O4 | 295822720 |
| Pos_00489 | Positive | 4.916167 | 238.0938 | Biopterin; Aif | C9H11N5O3 | 194207760 |
| Pos_00412 | Positive | 6.90765 | 119.0858 | Beta-Methylstyrene | C9H10 | 655039936 |
| Pos_00511 | Positive | 0.9078667 | 118.0863 | Betaine | C5H11NO2 | 19740377088 |
| Pos_00014 | Positive | 5.708717 | 169.0759 | Beta-Carboline | C11H8N2 | 378430912 |
| Neg_00264 | Negative | 0.8908167 | 215.0331 | Bergapten | C12H8O4 | 744745152 |
| Pos_00388 | Positive | 6.322484 | 119.0494 | Benzofuran | C8H6O | 254724880 |
| Neg_00176 | Negative | 6.241883 | 121.0285 | Benzoate | C7H6O2 | 4103987456 |
| Pos_00387 | Positive | 6.879783 | 118.0653 | Benzeneacetonitrile | C8H7N | 2093232384 |
| Pos_00065 | Positive | 1.740867 | 122.0602 | Benzamide | C7H7NO | 477406656 |
| Pos_00639 | Positive | 5.6251 | 347.1232 | Bavachin | C20H20O4 | 161669824 |
| Pos_00402 | Positive | 5.0273 | 128.0709 | Baikiain | C6H9NO2 | 1409671808 |
| Neg_00043 | Negative | 6.536033 | 445.0786 | Baicalin | C21H18O11 | 655178688 |
| Neg_00196 | Negative | 6.802633 | 187.0973 | Azelaic Acid | C9H16O4 | 9007766528 |
| Pos_00046 | Positive | 7.8261 | 263.1278 | Austricine | C15H18O4 | 337110432 |
| Pos_00613 | Positive | 7.074533 | 239.139 | Atractylone | C15H20O | 194305136 |
| Pos_00649 | Positive | 4.292133 | 253.1181 | Atractylenolide I | C15H18O2 | 693788864 |
| Pos_00635 | Positive | 0.9357833 | 455.1163 | Asperulosidic Acid | C18H24O12 | 617554112 |
| Neg_00256 | Negative | 2.799733 | 175.0247 | Ascorbate | C6H8O6 | 503692064 |
| Pos_00660 | Positive | 8.508817 | 385.1902 | Artesunate | C19H28O8 | 54567728 |
| Pos_00640 | Positive | 1.657617 | 305.1341 | Artemisinin | C15H22O5 | 4756142592 |
| Pos_00628 | Positive | 8.634533 | 335.1821 | Arnicolid C | C19H26O5 | 196077952 |
| Neg_00164 | Negative | 6.32575 | 233.046 | Armillarisin A | C12H10O5 | 116658056 |
| Pos_00083 | Positive | 5.152333 | 175.123 | Arginine | C6H14N4O2 | 121589216 |
| Neg_00316 | Negative | 8.018367 | 371.1519 | Arctigenin | C21H24O6 | 69357280 |
| Neg_00332 | Negative | 1.099667 | 151.0607 | Arabitol(D) | C5H12O5 | 1212617344 |
| Neg_00122 | Negative | 0.8908167 | 165.0401 | Arabinonic Acid | C5H10O6 | 6251436032 |
| Pos_00609 | Positive | 7.464283 | 299.0913 | Apigenin 7,4'-Dimethyl Ether | C17H14O5 | 4908884480 |
| Neg_00085 | Negative | 7.68345 | 269.0464 | Apigenin | C15H10O5 | 3390217472 |
| Pos_00107 | Positive | 6.5452 | 185.081 | Antiarol | C9H12O4 | 1962412416 |
| Pos_00755 | Positive | 9.052567 | 209.0598 | Anthraquinone | C14H8O2 | 61864112 |
| Pos_00371 | Positive | 6.419967 | 120.0447 | Anthranilate | C7H7NO2 | 209375616 |
| Neg_00057 | Negative | 4.86005 | 138.0552 | Anthranilate | C7H7NO2 | 97680720 |
| Pos_00429 | Positive | 7.54765 | 109.0653 | Anisole | C7H8O | 502463200 |
| Neg_00114 | Negative | 6.269733 | 107.0491 | Anisole | C7H8O | 216432688 |
| Pos_00493 | Positive | 5.750517 | 94.06574 | Aniline | C6H7N | 317130176 |
| Pos_00305 | Positive | 5.708717 | 475.1916 | Amygdalin | C20H27NO11 | 166579168 |
| Neg_00348 | Negative | 5.72385 | 456.1532 | Amygdalin | C20H27NO11 | 260960960 |
| Pos_00293 | Positive | 2.309217 | 158.0926 | Amphetamine | C9H13N | 3056979456 |
| Pos_00588 | Positive | 1.768617 | 348.0701 | Amp | C10H14N5O7P | 882216064 |
| Neg_00051 | Negative | 3.092033 | 346.0581 | Amp | C10H14N5O7P | 505010176 |
| Pos_00280 | Positive | 3.335433 | 144.0654 | Aminoadipate | C6H11NO4 | 2566268416 |
| Pos_00148 | Positive | 9.526617 | 337.2375 | Alprostadil | C20H34O5 | 178093616 |
| Pos_00458 | Positive | 9.359433 | 279.2322 | Alpha-Eleostearic Acid | C18H30O2 | 146519264 |
| Pos_00316 | Positive | 0.9078667 | 360.1499 | Alpha,Alpha-Trehalose | C12H22O11 | 279498112 |
| Neg_00174 | Negative | 0.9466833 | 341.1096 | Alpha,Alpha-Trehalose | C12H22O11 | 189803136 |
| Neg_00201 | Negative | 5.02705 | 161.0449 | Allose | C6H12O6 | 1825802496 |
| Pos_00622 | Positive | 5.24965 | 178.0499 | Alliin | C6H11NO3S | 493413792 |
| Pos_00623 | Positive | 5.152333 | 255.1336 | Alantolactone | C15H20O2 | 243055344 |
| Pos_00369 | Positive | 9.150167 | 299.0912 | Afrormosin | C17H14O5 | 238518736 |
| Pos_00285 | Positive | 3.238333 | 330.0598 | Adenosine 2,3-Cyclic Phosphate | C10H12N5O6P | 1708257024 |
| Pos_00348 | Positive | 3.765433 | 330.0598 | Adenosine 2',3'-Cyclic Monophosphate | C10H12N5O6P | 2514293760 |
| Pos_00308 | Positive | 4.818867 | 268.1043 | Adenosine | C10H13N5O4 | 17872805888 |
| Neg_00214 | Negative | 6.732584 | 266.0867 | Adenosine | C10H13N5O4 | 129408968 |
| Pos_00020 | Positive | 1.50505 | 136.0619 | Adenine | C5H5N5 | 1.17354E+11 |
| Neg_00181 | Negative | 1.521233 | 134.0464 | Adenine | C5H5N5 | 6670611456 |
| Pos_00259 | Positive | 5.13845 | 146.1176 | Acetylcholine | C7H16NO2+ | 389618176 |
| Pos_00456 | Positive | 3.2106 | 86.06076 | Acetone Cyanohydrin | C4H7NO | 14544550912 |
| Neg_00138 | Negative | 1.142217 | 85.0282 | Acetoacetaldehyde | C4H6O2 | 800512320 |
| Pos_00047 | Positive | 5.54155 | 152.0707 | Acetaminophen | C8H9NO2 | 326661248 |
| Pos_00128 | Positive | 11.54103 | 637.303 | Ac1L1X1Z | C23H46N6O13 | 6728246 |
| Pos_00076 | Positive | 7.283517 | 247.1328 | Abscisic Acid | C15H20O4 | 625956352 |
| Neg_00210 | Negative | 7.306267 | 263.1299 | Abscisic Acid | C15H20O4 | 311035136 |
| Neg_00015 | Negative | 6.802633 | 171.1024 | 9-Oxononanoic Acid | C9H16O3 | 532857408 |
| Pos_00372 | Positive | 11.69907 | 282.2787 | 9-Octadecenamide | C18H35NO | 119204064 |
| Pos_00661 | Positive | 6.322484 | 361.1388 | 9-{[(2E)-3,7-Dimethylocta-2,6-Dien-1-Yl]Oxy}-7H-Furo[3,2-G]Chromen-7-One | C21H22O4 | 107414800 |
| Neg_00116 | Negative | 9.74575 | 315.2549 | 9,10-Dihydroxystearic Acid | C18H36O4 | 1037701120 |
| Pos_00193 | Positive | 8.954833 | 277.2162 | 9(S)-Hot | C18H30O3 | 5393332736 |
| Pos_00733 | Positive | 8.620584 | 479.1709 | 8-Hydroxy-3-Methoxy-11-Oxo-1-Pentanoyl-6-Pentyl-11H-Dibenzo[B,E][1,4]Dioxepine-7-Carboxylic Acid | C25H28O8 | 56327220 |
| Pos_00711 | Positive | 7.561633 | 301.1069 | 8-(2,3-Dihydroxy-3-Methylbutyl)-7-Methoxychromen-2-One | C15H18O5 | 77749526528 |
| Pos_00732 | Positive | 6.6844 | 315.0858 | 7-Methylcapillarisin | C17H14O6 | 401647616 |
| Neg_00350 | Negative | 7.3342 | 313.0737 | 7-Methylcapillarisin | C17H14O6 | 96338360 |
| Pos_00034 | Positive | 9.930717 | 219.1742 | 7-Isopropenyl-1,4A-Dimethyl-3,4,5,6,7,8-Hexahydro-2-Naphthalenone | C15H22O | 326919680 |
| Neg_00252 | Negative | 6.05975 | 177.0187 | 7,8-Dihydroxycoumarin | C9H6O4 | 565086016 |
| Pos_00593 | Positive | 10.19527 | 481.2948 | 6-Oxopristimerol | C30H40O5 | 31140610 |
| Neg_00111 | Negative | 8.854584 | 309.2088 | 6-Methylgingediol | C18H30O4 | 2052850304 |
| Pos_00674 | Positive | 9.275917 | 415.2112 | 6-[3-[(3,4-Dimethoxyphenyl)Methyl]-4-Methoxy-2-(Methoxymethyl)Butyl]-4-Methoxy-1,3-Benzodioxole | C24H32O7 | 385413760 |
| Pos_00113 | Positive | 1.352417 | 130.0502 | 5-Oxoproline | C5H7NO3 | 19275169792 |
| Neg_00212 | Negative | 1.354383 | 128.0344 | 5-Oxoproline | C5H7NO3 | 22396233728 |
| Pos_00459 | Positive | 8.383317 | 313.107 | 5-Hydroxy-4',7-Dimethoxy-6-Methylflavone | C18H16O5 | 63566140 |
| Pos_00463 | Positive | 8.606533 | 283.097 | 5,7-Dimethoxyflavone | C17H14O4 | 230602576 |
| Pos_00758 | Positive | 5.221867 | 207.0651 | 5,7-Dimethoxy-2H-Chromen-2-One | C11H10O4 | 166106656 |
| Pos_00535 | Positive | 7.074533 | 287.0909 | 5,7-Dihydroxy-6-Methoxy-2-Phenyl-3,4-Dihydro-2H-1-Benzopyran-4-One | C16H14O5 | 376768480 |
| Pos_00534 | Positive | 5.61115 | 595.1644 | 5,7-Dihydroxy-2-(4-Hydroxyphenyl)-6,8-Bis[3,4,5-Trihydroxy-6-(Hydroxymethyl)Oxan-2-Yl]Chromen-4-One | C27H30O15 | 606511872 |
| Pos_00604 | Positive | 7.519834 | 331.0815 | 5,7,4'-Trihydroxy-3',6-Dimethoxyflavone | C17H14O7 | 445822016 |
| Pos_00731 | Positive | 8.397284 | 237.0759 | 5,6,7-Trimethoxycoumarin | C12H12O5 | 283924288 |
| Pos_00210 | Positive | 4.93005 | 144.0478 | 5-(2-Hydroxyethyl)-4-Methylthiazole | C6H9NOS | 2069299968 |
| Pos_00395 | Positive | 5.096867 | 106.0654 | 4-Vinylpyridine | C7H7N | 228965488 |
| Pos_00475 | Positive | 9.275917 | 167.1064 | 4-Tert-Butylcatechol | C10H14O2 | 673780224 |
| Pos_00495 | Positive | 5.680867 | 174.0552 | 4-Quinolinecarboxylic Acid | C10H7NO2 | 826897664 |
| Neg_00236 | Negative | 4.901883 | 115.0391 | 4-Oxopentanoic Acid | C5H8O3 | 221112688 |
| Pos_00474 | Positive | 2.03195 | 140.0342 | 4-Nitrophenol | C6H5NO3 | 20963403776 |
| Neg_00234 | Negative | 7.2364 | 138.0187 | 4-Nitrophenol | C6H5NO3 | 1139217664 |
| Pos_00596 | Positive | 7.199917 | 222.1126 | 4-Methoxy-6-Methyl-2H,5H,6H,7H,8H-[1,3]Dioxolo[4,5-G]Isoquinoline | C12H15NO3 | 204344240 |
| Neg_00302 | Negative | 7.431983 | 267.0671 | 4'-Me Ether-4',7-Dihydroxyflavone | C16H12O4 | 207305168 |
| Pos_00312 | Positive | 13.41232 | 136.1119 | 4-Isopropylaniline | C9H13N | 275475296 |
| Pos_00689 | Positive | 3.044183 | 139.0391 | 4-Hydroxybenzoate | C7H6O3 | 431821280 |
| Pos_00332 | Positive | 6.224933 | 123.0444 | 4-Hydroxybenzaldehyde | C7H6O2 | 801696448 |
| Pos_00506 | Positive | 1.75475 | 110.0604 | 4-Hydroxyaniline | C6H7NO | 10576780288 |
| Pos_00097 | Positive | 6.475616 | 153.0546 | 4-Hydroxy-3-Methoxy-Benzaldehyde | C8H8O3 | 549887424 |
| Pos_00331 | Positive | 5.54155 | 190.0499 | 4-Hydroxy-2-Quinolinecarboxylic Acid | C10H7NO3 | 1335903872 |
| Neg_00061 | Negative | 5.5425 | 188.0353 | 4-Hydroxy-2-Quinolinecarboxylic Acid | C10H7NO3 | 225176688 |
| Pos_00358 | Positive | 1.491167 | 146.0925 | 4-Guanidinobutanoate | C5H11N3O2 | 3685777408 |
| Neg_00130 | Negative | 0.9606333 | 119.0338 | 4-Deoxyerythronic Acid | C4H8O4 | 155668688 |
| Pos_00629 | Positive | 7.436383 | 423.1066 | 4'-Demethylpodophyllotoxin | C21H20O8 | 664839936 |
| Pos_00272 | Positive | 6.322484 | 165.055 | 4-Coumarate | C9H8O3 | 2131390720 |
| Pos_00442 | Positive | 6.503467 | 152.0706 | 4-Aminophenylacetic Acid | C8H9NO2 | 367835872 |
| Pos_00367 | Positive | 0.9078667 | 104.071 | 4-Aminobutanoate | C4H9NO2 | 2846914560 |
| Neg_00223 | Negative | 4.577783 | 102.055 | 4-Aminobutanoate | C4H9NO2 | 127818656 |
| Pos_00716 | Positive | 8.480917 | 285.0761 | 4',7-Dihydroxy-5-Methoxyisoflavone | C16H12O5 | 11059627008 |
| Pos_00374 | Positive | 5.54155 | 162.0552 | 4,6-Dihydroxyquinoline | C9H7NO2 | 221802992 |
| Pos_00631 | Positive | 8.49485 | 315.0858 | 4',5-Dihydroxy-3,7-Dimethoxyflavone | C17H14O6 | 3847234560 |
| Pos_00015 | Positive | 6.92155 | 165.0912 | 4-(4-Hydroxyphenyl)Butan-2-One | C10H12O2 | 14042801152 |
| Pos_00116 | Positive | 5.987884 | 151.0754 | 4-(2-Hydroxyethyl)-2-Methoxyphenol | C9H12O3 | 279052928 |
| Pos_00261 | Positive | 0.9496334 | 96.04482 | 3-Pyridinol | C5H5NO | 7419269632 |
| Pos_00422 | Positive | 6.823983 | 161.0963 | 3-Methyl-4-Phenyl-3-Buten-2-One | C11H12O | 310510016 |
| Neg_00151 | Negative | 2.07755 | 138.0187 | 3-Hydroxypicolinic Acid | C6H5NO3 | 2829859840 |
| Neg_00096 | Negative | 11.35617 | 271.2288 | 3-Hydroxyhexadecanoic Acid | C16H32O3 | 183413952 |
| Neg_00254 | Negative | 5.4031 | 123.0443 | 3-Hydroxybenzyl Alcohol | C7H8O2 | 1025729024 |
| Neg_00008 | Negative | 5.6818 | 137.0233 | 3-Hydroxybenzoate | C7H6O3 | 19814940672 |
| Pos_00496 | Positive | 0.9635333 | 154.0499 | 3-Hydroxyanthranilate | C7H7NO3 | 1018475072 |
| Neg_00062 | Negative | 5.76585 | 152.0349 | 3-Hydroxyanthranilate | C7H7NO3 | 886718144 |
| Pos_00636 | Positive | 7.561633 | 323.088 | 3-Hydroxy-9,10-Dimethoxypterocarpan | C17H16O5 | 1337677312 |
| Pos_00012 | Positive | 7.172033 | 138.0914 | 3-Dimethylaminophenol | C8H11NO | 113224184 |
| Pos_00405 | Positive | 1.685367 | 102.0561 | 3-Azetidinecarboxylic Acid | C4H7NO2 | 1366203264 |
| Neg_00084 | Negative | 5.66795 | 113.0597 | 3511 - 40.0 Ev | C6H10O2 | 110850560 |
| Pos_00761 | Positive | 8.49485 | 221.1535 | 3,7-Dimethyl-6-Octenyl Acetate | C12H22O2 | 213119424 |
| Pos_00185 | Positive | 3.01645 | 185.0447 | 3,5-Dihydroxy-4-Methoxybenzoic Acid | C8H8O5 | 300682880 |
| Pos_00658 | Positive | 1.962633 | 275.1235 | 3',5-Dihydroxy-3,4'-Dimethoxydihydrostilbene | C16H18O4 | 313664320 |
| Pos_00512 | Positive | 4.763317 | 346.0539 | 3',5'-Cyclic Gmp | C10H12N5O7P | 491502272 |
| Neg_00153 | Negative | 5.4588 | 149.0239 | 3,4-Methylenedioxybenzaldehyde | C8H6O3 | 287060640 |
| Neg_00155 | Negative | 5.138583 | 151.0392 | 3,4-Dihydroxyphenylacetaldehyde | C8H8O3 | 1257452288 |
| Pos_00688 | Positive | 1.865617 | 198.076 | 3,4-Dihydroxy-L-Phenylalanine | C9H11NO4 | 351909280 |
| Neg_00088 | Negative | 5.905633 | 196.0617 | 3,4-Dihydroxy-L-Phenylalanine | C9H11NO4 | 222852640 |
| Pos_00111 | Positive | 5.638983 | 155.034 | 3,4-Dihydroxybenzoate | C7H6O4 | 120650960 |
| Neg_00047 | Negative | 5.249933 | 153.0188 | 3,4-Dihydroxybenzoate | C7H6O4 | 15611806720 |
| Pos_00621 | Positive | 3.54355 | 185.0448 | 3,4,5-Trihydroxybenzoic Acid Methyl Ester | C8H8O5 | 646296384 |
| Pos_00042 | Positive | 6.6427 | 477.1034 | 3,4,5-Trihydroxy-6-{[5-Hydroxy-2-(3-Hydroxy-4-Methoxyphenyl)-4-Oxo-4H-Chromen-7-Yl]Oxy}Oxane-2-Carboxylic Acid | C22H20O12 | 544208704 |
| Neg_00250 | Negative | 5.430883 | 181.0503 | 3-(4-Hydroxyphenyl)Lactate | C9H10O4 | 2416464896 |
| Pos_00317 | Positive | 7.979217 | 163.039 | 3 Hydroxycoumarin | C9H6O3 | 473702816 |
| Pos_00375 | Positive | 2.309217 | 166.0725 | 2-Pyrazinoylguanidine | C6H7N5O | 1323907328 |
| Neg_00209 | Negative | 1.044133 | 145.0134 | 2-Oxoglutarate | C5H6O5 | 312147616 |
| Pos_00423 | Positive | 4.389117 | 88.11264 | 2-Methylbutylamine | C5H13N | 688037632 |
| Neg_00133 | Negative | 6.32575 | 119.0493 | 2-Methylbenzaldehyde | C8H8O | 2141289984 |
| Pos_00618 | Positive | 5.54155 | 361.1422 | 2-Methoxy-3-(3-Methyl-2-Butenyl)-4,4'-Dihydroxychalcone | C21H22O4 | 416543520 |
| Neg_00228 | Negative | 5.76585 | 136.0394 | 2-Hydroxybenzamide | C7H7NO2 | 432252864 |
| Neg_00117 | Negative | 5.863767 | 135.0445 | 2'-Hydroxyacetophenone | C8H8O2 | 2847353344 |
| Neg_00073 | Negative | 6.185767 | 131.0705 | 2-Hydroxy-4-Methylpentanoic Acid | C6H12O3 | 1727948288 |
| Pos_00438 | Positive | 6.43395 | 137.0598 | 2-Hydroxy-4-Methylbenzaldehyde | C8H8O2 | 10355458048 |
| Pos_00069 | Positive | 0.6989667 | 169.0457 | 2-Hydroxy-4-Methoxybenzoic Acid | C8H8O4 | 3698524416 |
| Neg_00333 | Negative | 5.389167 | 165.019 | 2H-1,3-Benzodioxole-5-Carboxylic Acid | C8H6O4 | 278341408 |
| Neg_00309 | Negative | 1.142217 | 111.0078 | 2-Furoate | C5H4O3 | 11405786112 |
| Pos_00426 | Positive | 1.060833 | 99.04444 | 2-Furanmethanol | C5H6O2 | 448590528 |
| Pos_00365 | Positive | 4.818867 | 252.1089 | 2'-Deoxyadenosine | C10H13N5O3 | 571329216 |
| Pos_00441 | Positive | 1.43575 | 83.04974 | 2-Cyclopenten-1-One | C5H6O | 248148576 |
| Pos_00334 | Positive | 5.72265 | 110.0603 | 2-Aminophenol | C6H7NO | 380712512 |
| Neg_00246 | Negative | 1.437767 | 116.0342 | 2-Acetamidoacetic Acid | C4H7NO3 | 204814384 |
| Pos_00018 | Positive | 8.954833 | 207.1741 | 2,6-Di-Tert-Butylphenol | C14H22O | 145768880 |
| Pos_00027 | Positive | 8.676333 | 221.1535 | 2,6-Di-Tert-Butylcyclohexa-2,5-Diene-1,4-Dione | C14H20O2 | 551374336 |
| Pos_00663 | Positive | 12.03422 | 108.0813 | 2,6-Dimethylpyridine | C7H9N | 165519296 |
| Pos_00123 | Positive | 6.935534 | 195.1014 | 2,6-Dimethoxy-4-(Prop-2-En-1-Yl)Phenol | C11H14O3 | 829222720 |
| Neg_00237 | Negative | 6.297733 | 153.0189 | 2,6-Dihydroxybenzoate | C7H6O4 | 3583082752 |
| Neg_00227 | Negative | 5.76585 | 153.0189 | 2,5-Dihydroxybenzoate | C7H6O4 | 3346784256 |
| Pos_00074 | Positive | 5.890033 | 153.0546 | 2',4'-Dihydroxyacetophenone | C8H8O3 | 532428768 |
| Neg_00270 | Negative | 13.68972 | 153.0188 | 2,3-Dihydroxybenzoate | C7H6O4 | 1390321664 |
| Neg_00157 | Negative | 5.905633 | 151.0396 | 2',3'-Dihydroxyacetophenone | C8H8O3 | 2917465856 |
| Neg_00268 | Negative | 5.235967 | 255.0518 | 2,3-Dihydroxy-2-[(4-Hydroxyphenyl)Methyl]Butanedioic Acid | C11H12O7 | 232276672 |
| Pos_00532 | Positive | 7.36685 | 213.0758 | 2,3-Dihydroxy-1-(4-Hydroxy-3-Methoxyphenyl)Propan-1-One | C10H12O5 | 862470656 |
| Pos_00407 | Positive | 5.764483 | 121.0652 | 2,3-Dihydrobenzofuran | C8H8O | 1082082432 |
| Neg_00093 | Negative | 3.763433 | 328.0467 | 2',3'-Cyclic Amp | C10H12N5O6P | 1701857280 |
| Neg_00069 | Negative | 8.24145 | 245.046 | 2,3',4,6-Tetrahydroxybenzophenone | C13H10O5 | 89595392 |
| Pos_00050 | Positive | 5.4581 | 156.1382 | 2,2,6,6-Tetramethylpiperidin-4-One | C9H17NO | 260094368 |
| Pos_00603 | Positive | 9.038683 | 433.149 | 2-(3,4-Dimethoxyphenyl)-3,5,6,7,8-Pentamethoxy-4H-Chromen-4-One | C22H24O9 | 79258096 |
| Neg_00351 | Negative | 5.83575 | 421.1664 | 2-(2,4-Dihydroxyphenyl)-5,7-Dihydroxy-3,8-Bis(3-Methylbut-2-En-1-Yl)-4H-Chromen-4-One | C25H26O6 | 108086680 |
| Pos_00381 | Positive | 5.513783 | 114.0917 | 1-Piperidinecarboxaldehyde | C6H11NO | 913312320 |
| Pos_00024 | Positive | 6.782017 | 163.1116 | 1-Phenylpentan-1-One | C11H14O | 547009856 |
| Neg_00100 | Negative | 5.570384 | 108.0445 | 1-Methyl-2-Pyrrolecarboxaldehyde | C6H7NO | 109294096 |
| Pos_00346 | Positive | 5.736483 | 231.1125 | 1-Methyl-1H,2H,3H,4H,9H-Pyrido[3,4-B]Indole-3-Carboxylic Acid | C13H14N2O2 | 296365824 |
| Pos_00400 | Positive | 5.013417 | 96.04483 | 1H-Pyrrole-2-Carboxaldehyde | C5H5NO | 845938880 |
| Neg_00097 | Negative | 2.07755 | 94.02867 | 1H-Pyrrole-2-Carboxaldehyde | C5H5NO | 1019867840 |
| Pos_00494 | Positive | 5.277433 | 134.0602 | 1H-Indol-3-Yloxidanesulfonic Acid | C8H7NO4S | 302268320 |
| Pos_00347 | Positive | 5.6251 | 217.0969 | 1H,2H,3H,4H,9H-Pyrido[3,4-B]Indole-3-Carboxylic Acid | C12H12N2O2 | 2098221568 |
| Pos_00580 | Positive | 10.32085 | 471.3461 | 18Alpha-Glycyrrhetinic Acid | C30H46O4 | 84552088 |
| Pos_00549 | Positive | 13.52602 | 338.3416 | 13(Z)-Docosenamide | C22H43NO | 821215168 |
| Neg_00126 | Negative | 4.92965 | 325.1093 | 1,5-Bis(4-Hydroxy-3-Methoxyphenyl)-1,4-Pentadien-3-One | C19H18O5 | 73096384 |
| Neg_00072 | Negative | 8.380834 | 281.1404 | 1,5,9-Trihydroxy-5,7,7-Trimethyl-4,5A,6,8,8A,9-Hexahydro-1H-Azuleno[5,6-C]Furan-3-One | C15H22O5 | 94887648 |
| Pos_00693 | Positive | 6.0715 | 159.0442 | 1,4-Naphthoquinone | C10H6O2 | 221755584 |
| Pos_00581 | Positive | 8.913116 | 253.1801 | 1,4-Dihydroxy-1,4-Dimethyl-7-(Propan-2-Ylidene)-Decahydroazulen-6-One | C15H24O3 | 238926880 |
| Pos_00673 | Positive | 10.13962 | 522.3555 | 1,2-Dierucoyl-Sn-Glycero-3-Phosphocholine | C26H52NO7P | 26502684 |
| Pos_00507 | Positive | 5.1246 | 127.0392 | 1,2,3-Trihydroxybenzene | C6H6O3 | 909537664 |
| Neg_00070 | Negative | 6.03165 | 357.0632 | 1-(3,4-Dihydroxyphenyl)-6,7-Dihydroxy-1,2-Dihydronaphthalene-2,3-Dicarboxylic Acid | C18H14O8 | 282006272 |
| Pos_00679 | Positive | 6.0297 | 224.128 | {2-[Hydroxy(4-Oxocyclohexa-2,5-Dien-1-Ylidene)Methoxy]Ethyl}Trimethylazanium | C12H18NO3 | 417791840 |
| Pos_00708 | Positive | 8.634533 | 277.18 | [6]-Shogaol | C17H24O3 | 1101261440 |
| Pos_00089 | Positive | 8.006933 | 277.1778 | [6]-Gingerol | C17H26O4 | 288906944 |
| Neg_00184 | Negative | 1.5629 | 89.02324 | (S)-Lactate | C3H6O3 | 10000217088 |
| Pos_00168 | Positive | 9.707666 | 317.1716 | (S)-[6]Gingerol | C17H26O4 | 134815328 |
| Neg_00327 | Negative | 5.80775 | 151.0392 | (R)-Mandelate | C8H8O3 | 776829568 |
| Neg_00056 | Negative | 1.298817 | 135.0288 | (R)-(+)-2-Hydroxysuccinic Acid\|D-(+)-Malic Acid\|(2R)-2-Hydroxybutanedioic Acid\|D-Hydroxybutanedioic Acid | C4H6O5 | 226721664 |
| Pos_00446 | Positive | 6.949383 | 163.0756 | (E)-Methyl Ester 3-Phenyl-2-Propenoic Acid | C10H10O2 | 1304299136 |
| Pos_00304 | Positive | 3.2106 | 344.134 | (E)-3-[2-[(2S,3R,4S,5S,6R)-3,4,5-Trihydroxy-6-(Hydroxymethyl)Oxan-2-Yl]Oxyphenyl]Prop-2-Enoic Acid | C15H18O8 | 189409856 |
| Pos_00016 | Positive | 8.927033 | 147.0806 | (3E)-4-Phenylbut-3-En-2-One | C10H10O | 1033426112 |
| Pos_00537 | Positive | 6.5174 | 447.0921 | (2S,3S,4S,5R,6S)-3,4,5-Trihydroxy-6-[5-Hydroxy-2-(4-Hydroxyphenyl)-4-Oxochromen-7-Yl]Oxyoxane-2-Carboxylic Acid | C21H18O11 | 2686247424 |
| Neg_00217 | Negative | 5.933583 | 283.084 | (2S,3S,4S,5R,6S)-3,4,5-Trihydroxy-6-(4-Methylphenoxy)Oxane-2-Carboxylic Acid | C13H16O7 | 126115176 |
| Pos_00052 | Positive | 5.277433 | 413.1425 | (2S,3R)-2-[({9-[(2R,3R,4S,5R)-3,4-Dihydroxy-5-(Hydroxymethyl)Oxolan-2-Yl]-9H-Purin-6-Yl}Carbamoyl)Amino]-3-Hydroxybutanoic Acid | C15H20N6O8 | 83407184 |
| Neg_00055 | Negative | 6.52195 | 361.0952 | (2S)-3-(3,4-Dihydroxyphenyl)-2-[(E)-3-(3,4-Dihydroxyphenyl)Prop-2-Enoyl]Oxypropanoic Acid\|Rosemary Acid\|Rosmarinic Acid\|Rosmarinate\|(R)-O-(3,4-Dihydroxycinnamoyl)-3-(3,4- Dihydroxyphenyl)Lactic Acid\|7 | C18H16O8 | 134943376 |
| Pos_00245 | Positive | 5.513783 | 295.1287 | (2S)-2-Amino-4-{[(1S)-1-Carboxy-2-Phenylethyl]Carbamoyl}Butanoic Acid | C14H18N2O5 | 123217528 |
| Pos_00264 | Positive | 9.9028 | 331.2845 | (2S)-2,3-Dihydroxypropyl Hexadecanoate | C19H38O4 | 40567656 |
| Pos_00701 | Positive | 5.0273 | 289.0913 | (2-Methyl-4-Oxopyran-3-Yl) Beta-D-Glucopyranoside | C12H16O8 | 292495584 |
| Pos_00769 | Positive | 5.61115 | 245.1858 | (2E,6E)-Farnesol | C15H26O | 514020864 |
| Pos_00117 | Positive | 5.6251 | 113.0601 | (2E,4E)-Hexa-2,4-Dienoic Acid | C6H8O2 | 460022496 |
| Pos_00730 | Positive | 6.419967 | 330.1336 | (2E)-N-[2-Hydroxy-2-(4-Hydroxyphenyl)Ethyl]-3-(4-Hydroxy-3-Methoxyphenyl)Prop-2-Enamide | C18H19NO5 | 103166936 |
| Pos_00149 | Positive | 7.631267 | 191.0703 | (2E)-3-(3,4-Dimethoxyphenyl)Prop-2-Enoic Acid | C11H12O4 | 587487872 |
| Pos_00238 | Positive | 7.631267 | 221.0809 | (2E)-3-(3,4,5-Trimethoxyphenyl)Prop-2-Enoic Acid | C12H14O5 | 545121216 |
| Pos_00370 | Positive | 8.885283 | 285.1114 | (2E)-1-(2-Hydroxy-4,6-Dimethoxyphenyl)-3-Phenylprop-2-En-1-One | C17H16O4 | 147734880 |
| Pos_00253 | Positive | 11.2709 | 830.5698 | (2-{[(2R)-2,3-Bis[(5Z,8Z,11Z,14Z)-Icosa-5,8,11,14-Tetraenoyloxy]Propyl Phosphonato]Oxy}Ethyl)Trimethylazanium | C48H80NO8P | 77983168 |
| Pos_00172 | Positive | 8.467033 | 221.1901 | (1R,4R,6R,10S)-4,12,12-Trimethyl-9-Methylene-5-Oxatricyclo[8.2.0.0~4,6~]Dodecane | C15H24O | 701293376 |
| Pos_00133 | Positive | 8.982767 | 257.1898 | (+)-Bakuchiol | C18H24O | 200639184 |
| Pos_00444 | Positive | 5.890033 | 153.1274 | (-)-Trans-Carveol | C10H16O | 605108480 |
| Pos_00120 | Positive | 7.3391 | 151.112 | (-)-Myrtenal | C10H14O | 557379520 |
| Neg_00067 | Negative | 5.80775 | 289.073 | (-)-Epicatechin | C15H14O6 | 138105248 |
| Pos_00002 | Positive | 7.90965 | 237.1851 | (-)-Curcumol | C15H24O2 | 265489920 |
| Pos_00488 | Positive | 0.9496334 | 160.0968 | (-)-Betonicine | C7H13NO3 | 644155520 |

Note: ESI, electrospray ionization; RT, retention time (min); m/z, mass-to-charge ratio.

Video S1. Movie of the molecular dynamics’ trajectory.

Note: The movie shows the diffusion of the scutellarein from the initial docking-predicted binding position. The scutellarein-XDH complex was stable over the entire simulation. XDH, xanthine dehydrogenase.

References

[1] J.K. Kim, W.J. Kim, J.M. Hyun, J.S. Lee, J.G. Kwon, C. Seo, M.J. Song, C.W. Choi, S.S. Hong, K. Park, P. Kim, H. Sung, J.K. Lee, Y. Choi, Salvia plebeia extract inhibits xanthine oxidase activity in vitro and reduces serum uric acid in an animal model of hyperuricemia, Planta Med. 83 (2017) 1335-1341. https://doi.org/ 10.1055/s-0043-111012.

[2] Y. Liu, X.-H. Liu, S. Zhou, H. Gao, G.-L. Li, W.-J. Guo, X.-Y. Fang, W. Wang, Perillanolides A and B, new monoterpene glycosides from the leaves of Perilla frutescens, Rev. Bras. Farmacogn.-Braz. J. Pharmacogn. 27 (2017) 564-568. https://doi.org/ 10.1016/j.bjp.2017.06.003.

[3] M.J. Sanz, M.L. Ferrandiz, M. Cejudo, M.C. Terencio, B. Gil, G. Bustos, A. Ubeda, R. Gunasegaran, M.J. Alcaraz, Influence of a series of natural flavonoids on free radical generating systems and oxidative stress, Xenobiotica. 24 (1994) 689-699. https://doi.org/ 10.3109/00498259409043270.
